# Elucidating the effect of spatial localization on metabolic systems

**DOI:** 10.1098/rsif.2025.1328

**Published:** 2026-10-28

**Authors:** Abdulrahman Almutairi, J. Krishnan

**Affiliations:** ^1^ Department of Chemical Engineering, Sargent Centre for Process Systems Engineering, Imperial College London, London, UK; ^2^ Institute for Systems and Synthetic Biology, Imperial College London, South Kensington Campus, London, UK

**Keywords:** spatial localization, enzymes, motifs, trade-offs, compartment, reaction–diffusion

## Abstract

Spatial biology, both natural and engineered, is a rapidly developing area, propelled by the advent of new tools and technologies. At the intracellular level, this has recently involved the use of advanced techniques in spatial imaging, measurement and data processing, in systems biology and the creation of different types of artificial compartments in synthetic biology. However, there are still major gaps in our understanding of the effect of spatial organization on intracellular biomolecular networks. In this study, we focus on metabolic systems and combine bottom-up studies of basic building blocks and top-down studies of concrete exemplar cases to understand the effect of spatial localization/compartmentalization of species. We develop a systems framework with multiple strands. (i) Assessing the effect of localization of enzymes and substrates on a variety of metabolic motifs, revealing both structural invariants (shown analytically) and multiple unintuitive effects, specifically traced to localization (by analytically ruling out their presence in well-mixed systems). (ii) Assessing and revealing the hidden impact of spatial localization on standard metabolic control coefficients, while also introducing and analysing spatial control coefficients. (iii) Analysing how localization can be used in engineering metabolic networks, uncovering various kinds of trade-offs in the process. (iv) Examining the effect of localization in concrete exemplar cases. In so doing, we develop contrasts with analysis of systems which treat them as well mixed. Our analysis combining top-down approaches and bottom-up approaches from multiple perspectives transparently reveals a number of basic as well as subtle ways in which compartmentalization and spatial localization can impact metabolic networks. The results and the methods of analysis have important implications for systems and synthetic biology in a variety of settings.

## 1. Introduction

Spatial organization is a basic and pervasive theme occurring across complex systems spanning a broad range of contexts from the physical sciences to the social sciences. In the life sciences, in general, and cellular biology in particular, it has been historically simultaneously widely recognized and widely ignored. However, the past decade and a half has seen a steadily increasing interest in this theme, propelled by studies in a range of systems spanning bacteria [1–5] and eukaryotes [6–10], new techniques for spatial imaging and measurement [11–16], a rapidly emerging set of technologies for engineering compartmentalization in synthetic biology [17–21], and the discovery of membraneless compartments [22–26]. In the last few years, the development and use of spatial omics technologies have accelerated the study of the role of spatial organization at both sub-cellular and tissue levels, in a wide range of contexts.

Metabolic systems have been a central focal point in basic biology, biotechnology, systems and synthetic biology [27–30]. Investigations of metabolic systems have successfully incorporated modelling and systems frameworks based on kinetic modelling and particularly flux-balance analysis [27–32], complementing experimental work in a variety of contexts. This has been used for a range of goals, from understanding systems behaviour, making predictions which could be tested, as a basis for optimizing yield of metabolites for biotechnology, for the investigation of metabolic disorders [33–35], and for applications in synthetic biology. In some of these cases, modelling and computation are used with other systems tools such as optimization.

Different aspects of spatial organization have been experimentally investigated in metabolic systems, in both systems and synthetic biology. From the synthetic perspective, these include investigations ranging from the creation of artificial compartments to establishing localized patterns of species in metabolic and biochemical networks [7,36–39]. In addition, the considerable emerging technology in synthetic biology provides further experimental tools for creating different compartmental structures, which can be used as a tool for engineering/rewiring/manipulating metabolic networks [40,41]. From the systems biology perspective, the use of omics and imaging technologies allows us to map and quantify metabolites and proteins at both single cell and tissue levels, and the use of single-cell multi-omics and spatial multi-omics technologies provides important insights into the spatio-temporal behaviour of proteins at both cellular and subcellular levels [42–44]. This opens up new avenues to understand and characterize metabolic disorders, including cardiovascular disease and non-alcoholic fatty liver disease [45,46].

Although there have been significant experimental strides as mentioned above along with recent data-driven investigations using omics technologies, there have been relatively few systematic studies of the impact of spatial organization on metabolic and related biochemical systems. Different studies have probed basic aspects [47] or assessed the effect of spatial distribution of enzymes in optimizing systems [48]. There are also studies of such systems using compartmental ordinary differential equation (ODE) models [49,50]; however, as noted in a previous study, compartmental models suffer from important limitations which emerge from comparing them side-by-side with explicit spatial models [51]. In particular, a careful systematic systems-based dissection of the interplay between species compartmentalization/localization patterns and the behaviour of metabolic systems/networks is missing. In this paper, we create a bottom-up theoretical and systems framework for unravelling different facets of the interplay of spatial organization and metabolic networks. We focus on basic building blocks of larger metabolic networks and study simpler network motifs. This allows us to carefully pin down and understand key aspects of the interplay in a transparent and generalizable manner. We use kinetic and explicit spatial modelling to create a framework for addressing the following questions: (i) How does spatial localization of different species (enzymes, substrates) affect systems behaviour? How does it impact typical observables (e.g. spatial average or compartmental average concentrations of species)? These would be experimental readouts of interest, whether or not spatial organization is the explicit focal point in a biological investigation. (ii) How does localization impact metabolic systems with feedback? (iii) When spatial organization is present, how can we effectively quantify the sensitivity of species concentrations or fluxes to changes in concentrations or spatial factors? (iv) What are the various trade-offs associated with localizing species, and designing compartmentalization, while attempting to engineer systems behaviour? We complement these investigations with a focused top-down analysis of an exemplar case of glycolysis in *Saccharomyces cerevisiae* [52], which highlights and reinforces many conclusions from studies on motifs while also revealing new insights.

We employ computational work, along with analytical work: the latter providing key structural insights of the spatial systems on the one hand, while also tracing other effects specifically to spatial localization, by analytically showing their absence in well-mixed systems. By establishing contrasts with well-mixed systems and their analysis (studying the effect of localization vis-à-vis no localization, or studying the effects of hidden localization, not explicitly accounted for and studied in a lumped systems framework), we are able to reveal the essentially new complexities and dimensions introduced by localization. We also discuss in which respect quantifying sensitivity to spatial factors differs essentially from sensitivity to kinetic factors. Taken together, the study provides conceptual and foundational insights for spatial biology and a platform for both designing spatial organization in synthetic biology, and uncovering spatial organization in systems biology using data-driven approaches.

## 2. Methods

### 2.1. Goal of the study

Our goal is to develop a systems framework to carefully elucidate the interplay between spatial localization and metabolic systems. In so doing, we aim to provide insights in different contexts: systems biology, synthetic biology and cell-free systems. As a consequence, we focus on the most basic and recurrent aspects of metabolic systems and networks and on spatial localization which apply across this spectrum. On the network side, we combine extensive analysis of basic building blocks of networks (network motifs) along with top-down analysis of exemplar cases. For spatial organization, we focus on localization of enzymes and substrates. A schematic summary of the approach and focal point of the study is presented in figure 1.

**Figure 1.**
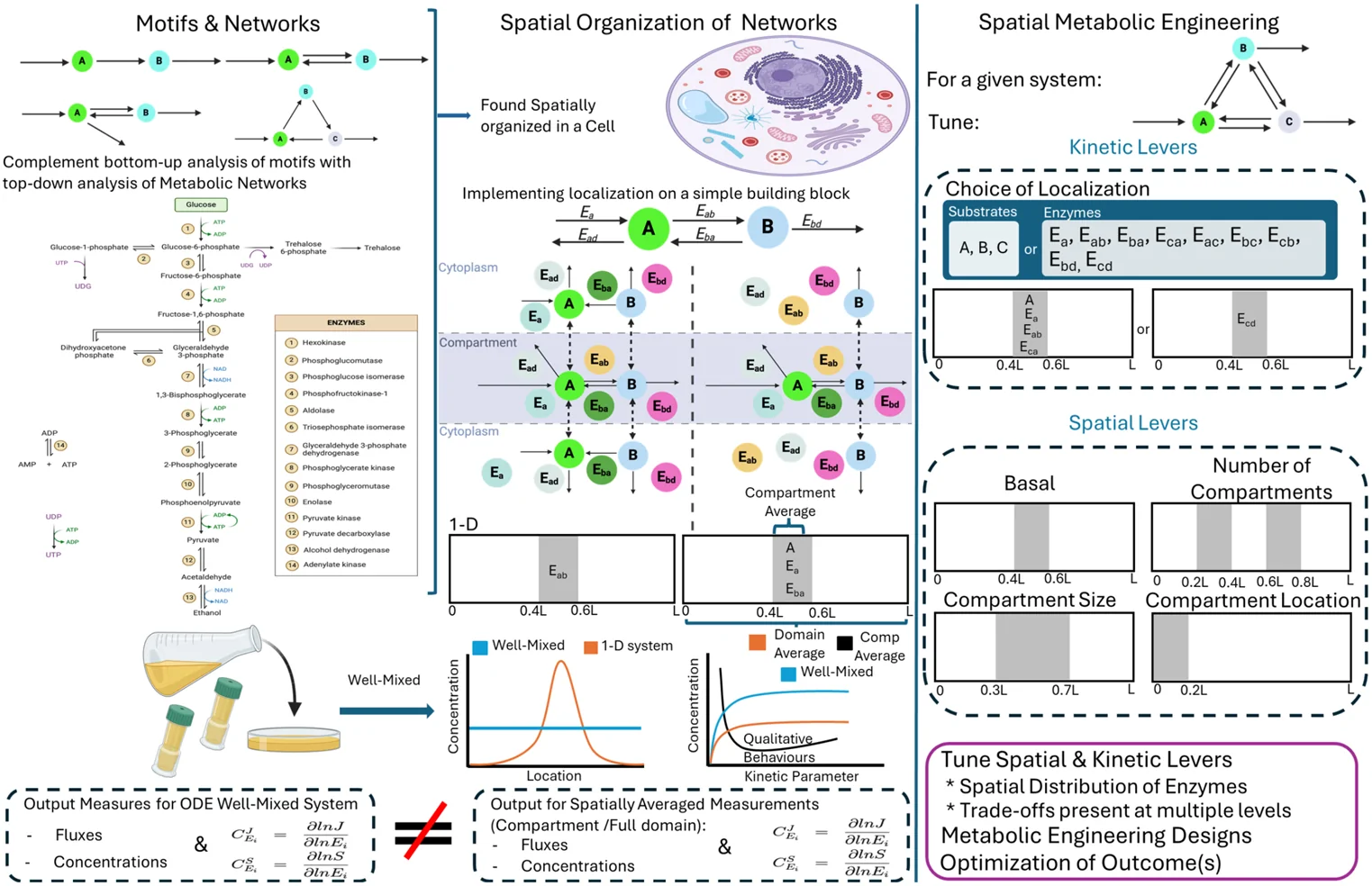
Schematic summary of the focus of the paper. Left column: Metabolic systems are analysed at different levels combining bottom-up study of basic motifs (a selection of motifs depicted) and a top-down analysis of a concrete exemplar case (glycolysis: network depicted). In the case where systems are well mixed, these are described by kinetic ODE models. Key outputs from these models are steady-state concentrations of species, fluxes and various control coefficients depicting dimensionless scaled (logarithmic) sensitivities of concentrations/fluxes to enzyme concentrations. Middle column: A simplified schematic indicating spatial organization in a cell and that it is far from a well-mixed system. The implementation of localization in simple building blocks is demonstrated in the context of a specific two-node motif, shown. A schematic depiction of localization in this motif of enzymes (responsible for A to B conversion) and substrates (substrate A) are shown side by side in terms of the network. The 1D spatial domain, along with the location of the (shaded) compartment where species are localized, is shown below this, indicating which species are localized. Sample schematic spatial profiles are depicted, along with curves depicting the dependence of key output measures (spatial averaged concentrations) on parameters. We see that different measures may have qualitatively different dependence on parameters, and these may not agree quantitatively and qualitatively with results obtained by ignoring spatial localization (ODE model, well-mixed systems). Key output measures are spatial averaged concentrations or fluxes (averaged over either the full spatial domain or specific compartments), and associated control coefficients, and these in general differ, quantitatively and even qualitatively from results obtained from ODE models. Right column: Depiction of basic steps in spatial metabolic engineering. For a given network, a choice of relevant species (enzymes or possibly substrates) to localize, on the one hand (kinetic levers) complemented by designing characteristics of compartments: size, location and number (spatial levers). By considering different options of kinetic and spatial levers, different kinds of designs may be obtained which could be used to improve and optimize systems behaviour [78].

### 2.2. Motivation for the approach

Combining bottom-up and top-down analyses provides both a methodological approach and insights across systems biology, synthetic biology and cell-free systems. In addition to the relevance of bottom-up approaches in synthetic biology (discussed below), the analysis we perform here naturally emerges if we aim to elucidate the effect of spatial localization in a concrete system in systems biology (such as the one we study). In this instance, aiming to get clear-cut cause-and-effect relationships and trace the origins of behaviour leads us naturally to such bottom-up analyses. The consideration of different motifs, the effect of enzyme–substrate feedback and the assessment and introduction of metabolic control coefficients stems from this.

### 2.3. Choice of networks: motifs

Metabolic networks are diverse, unstructured and contain many components. Our goal is to obtain clear-cut transparent and generalizable insights regarding basic aspects of the impact of spatial localization on metabolic systems/networks. Our analysis of networks involves a combination of bottom-up and top-down approaches. We first consider a variety of two-node and three-node motifs. In each of these cases, the nodes represent substrate variables. These are subject to interconversion, as well as influx and degradation/efflux (henceforth, we will use the terms efflux and degradation interchangeably). We consider a variety of two-node motifs with variations incorporating reversible/irreversible interconversion and degradation/efflux of either one or both species. Although all of these features could be subsumed within an overarching motif, it is useful to study these as separate motifs to facilitate transparent insights connecting motif structure to behaviour. In a similar way, we consider a variety of three-node motifs, which vary depending on the reversibility/irreversibility of interconversion between each pair of species, as well as which substrate species can be subject to efflux (we consider either one or two different species capable of efflux). Thus, our selection of motifs incorporates a range of network structures, which form the building blocks of metabolic networks: linear motifs, branched motifs and cyclic motifs.

In all these cases, the nodes represent the substrate species, whereas the arrows represent conversion mediated by enzymes. For the most part, unless otherwise mentioned, the enzyme concentrations are assumed to be fixed. The kinetic description of these reaction networks involves mass action kinetic descriptions of the basic steps. We employ mass action kinetic descriptions as this is sufficient for understanding the most basic and pervasive aspects of the interplay of spatial localization and metabolic networks. Furthermore, by adopting these descriptions rather than Michaelis–Menten kinetics, we reduce the number of kinetic parameters. Many of the insights continue to hold good even if Michaelis–Menten kinetic descriptions are used, something which is reinforced in our top-down study (which uses Michaelis–Menten descriptions). Additionally, we also examine motifs with substrate–enzyme feedback (e.g. substrate inhibition) to probe the effects of spatial organization there.

### 2.4. Choice of networks: exemplar system

This study is complemented by a focused top-down study of a specific network of the glycolytic pathway. Here, we employ a model calibrated against experimental data taken from the literature, incorporating a number of species, and the associated kinetic descriptions (typically Michaelis–Menten kinetics). In addition to having a number of biochemical steps, this system also has multiple distinctive features not seen in the simpler models/motifs (e.g. description of energetics, presence of bifunctional enzymes, etc.).

### 2.5. Spatial model

Our focus is on the most basic recurrent aspects of spatial localization in natural, synthetic and cell-free contexts. Therefore, we focus on localization of the key constituents of metabolic systems: enzymes and substrates, in spatial sub-domains of the full domain. We employ a spatially explicit partial differential equation (PDE) model in 1D describing the reaction kinetics and diffusion of the species (see figure 1, middle column). A restriction to one spatial dimension is sufficient as a starting point for the analysis here, since it already reveals the basic aspects of spatial localization. For simplicity, for the most part, we regard enzymes as non-diffusible and treat enzyme concentrations as parameters. When an enzyme is localized, we simply restrict its presence to the specific sub-domain. When we impose localization of substrates, we restrict the presence of substrates to a specific sub-domain of the overall spatial domain. There are two points to note. (i) Localization of substrates necessarily implies localization of enzymes responsible for producing these substrates (unless the reactant substrate happens to be localized, which is typically not the case). (ii) The substrate is allowed to diffuse in its localized region. In some instances to make comparisons to the case of no localization, we consider enzyme localization, which keeps the total amount of enzyme in the domain fixed (i.e. the local concentration of enzyme is increased in inverse proportion to the size of the localized compartment). In these instances, this feature is explicitly highlighted. We also emphasize: (i) we do not provide a mechanistically explicit description of what causes localization (e.g. localizing proteins, cytoskeleton, etc.), but instead focus on the *effects* of localization and (ii) we do not explicitly incorporate the membrane of the compartments, noting that the basic qualitative effects we reveal can be seen in both cases.

### 2.6. Output measures

In our analysis, we typically focus on spatially averaged concentrations of species, or spatially averaged fluxes of reactions, where the spatial averaging may be done either over the full spatial domain or over a compartment. These are natural output measures of interest (whether or not spatial aspects are the focal point in an investigation). When there is no spatial localization (well-mixed systems), these reduce to species concentrations and fluxes as seen in kinetic ODE models of the system. In some cases, we compute associated metabolic control coefficients to reveal new insights and facilitate comparison with results of ODE models.

### 2.7. Parameters and the focus of the analysis

The PDE models for motifs are dimensionless and involve both kinetic and spatial parameters (size of compartment, diffusivity of species, etc.). We choose representative values for both kinetic and spatial parameters in the motifs (also see appendices A and B). We first highlight a few points regarding the results we present, and how we analyse them. (i) The results presented are qualitative and semi-quantitative on the interplay of spatial localization and the kinetics. Thus, our focus is less on the actual numbers and more on notable key underlying trends and qualitative features. These features are seen in fairly broad parametric regions in a given motif, and are thus seen in multiple parameter sets. Furthermore, they also recur in other motifs, thus indicating that their occurrence is robust even to changes in the network. (ii) In some cases, the results arise from a key structural feature (also established analytically), whereas in other cases, it arises from a basic interplay of factors which recurs in different instances, and we transparently reveal its source. In some instances, our results reveal the presence of a surprising and unexpected basic behaviour, and analytical work reveals that is traceable solely to spatial localization. (iii) Many of our results focus on basic contrasts in behaviour either between two motifs, or a given motif with two different localization scenarios, or a contrast between the spatial system and the well-mixed system. In the former two cases, we draw basic conclusions and insights from well-defined contrasting cases (where all common parameters are the same). In the third case, we show analytically that a certain behaviour (even of spatial averages) cannot be seen in the well-mixed kinetic system, irrespective of any parameters. (iv) Our results were obtained by studying a variety of network motifs, spatial localization scenarios and parameters. We present a selection of these results striking a balance between highlighting striking results and an exhaustive presentation of all possibilities.

For our study of motifs presented here, we make certain basic parameter choices (though we have observed such behaviour in other regimes as well). We keep the rate constants for all interconversion reactions present, fixed at the same value. This allows us to make sensible contrasts between motifs. The only other way an effective interconversion rate is altered is either by spatial localization or by feedback (studied in the sub-section on feedback) and this is assessed and analysed directly as part of the analysis. The inflow rate constant is also held fixed. Typically, the only kinetic rate constant varied is the outflow rate constant. A broader analysis of the effect of kinetic parameters and species diffusivity underpins our study. In the exemplar case, parameters are taken from the literature in a model calibrated against experimental data.

## 3. Results

We first discuss our analysis of spatial localization on basic network structures: two-node and three-node motifs. Following this, we examine a selection of cases involving feedback between substrate and enzyme. We then revisit localization in basic motifs from a different focal point: that of metabolic control coefficients. We then extend our analysis from the perspective of spatial engineering. We complement these results with the analysis of a concrete exemplar case: glycolysis.

### 3.1. Examination of basic motifs

We first examine basic two-node and three-node motifs. In the most basic instance, there is no feedback between the substrate and enzyme. Our focus is on the effect of localization of either a specific enzyme or a specific substrate in a specified sub-domain. For the results here, we consider a sub-domain of size one-fifth of the full domain, centred at the middle of the domain. Other cases involving localization of multiple substrates or multiple enzymes, or localization of a species at multiple sub-domains will be presented in a subsequent sub-section in the context of engineering localization.

We focus on the spatially averaged concentration of particular species or the spatially averaged flux of particular reactions. We consider two kinds of averages: (i) average over the full spatial domain and (ii) average over the compartment. A basic example case (of a three-node motif with two outflows; see supplementary material, figure S1) reveals that spatial localization affects the average concentrations and fluxes, both averaged over the full domain and over the compartmental sub-domain. Our analysis hereafter aims to dig deeper and reveal basic and subtle features associated with localization. To that end, we present a succession of results from specific motifs.

#### 3.1.1. Structural features of the network underpin insulation of spatial averages of species concentrations (and fluxes) from effects of kinetic parameters

The first two cases present certain structural features (i.e. parameter independent), which arise when there is only one outflow species. We find in figure 2a that localization of an enzyme other than the one associated with outflow/degradation results in the spatially averaged concentration of the outflow variable (B) over the full domain being constant, as the kinetic parameter associated with the A to B reaction is varied. This feature is independent of the diffusivity and localization features (e.g. size of localization). The spatial average depends on only two kinetic parameters: the production rate of A and the efflux rate constant of B (and in this instance, the extent of localization). On the other hand, if the enzyme associated with the efflux of the (sole) outflow variable (B) is localized, then the spatial average of that species over the compartment (associated with the localization) is fixed, whereas the spatial average over the full domain is not, as seen in figure 2b. Again, this feature is independent of interconversion kinetic parameters, diffusivities and compartment size/location. In both instances, while the result was shown for a two-node motif with one outflow, they are valid for any basic two-node or three-node motif (or even a larger motif) with a single outflow, where an internal kinetic parameter is varied (this is shown analytically in appendix B).

**Figure 2.**
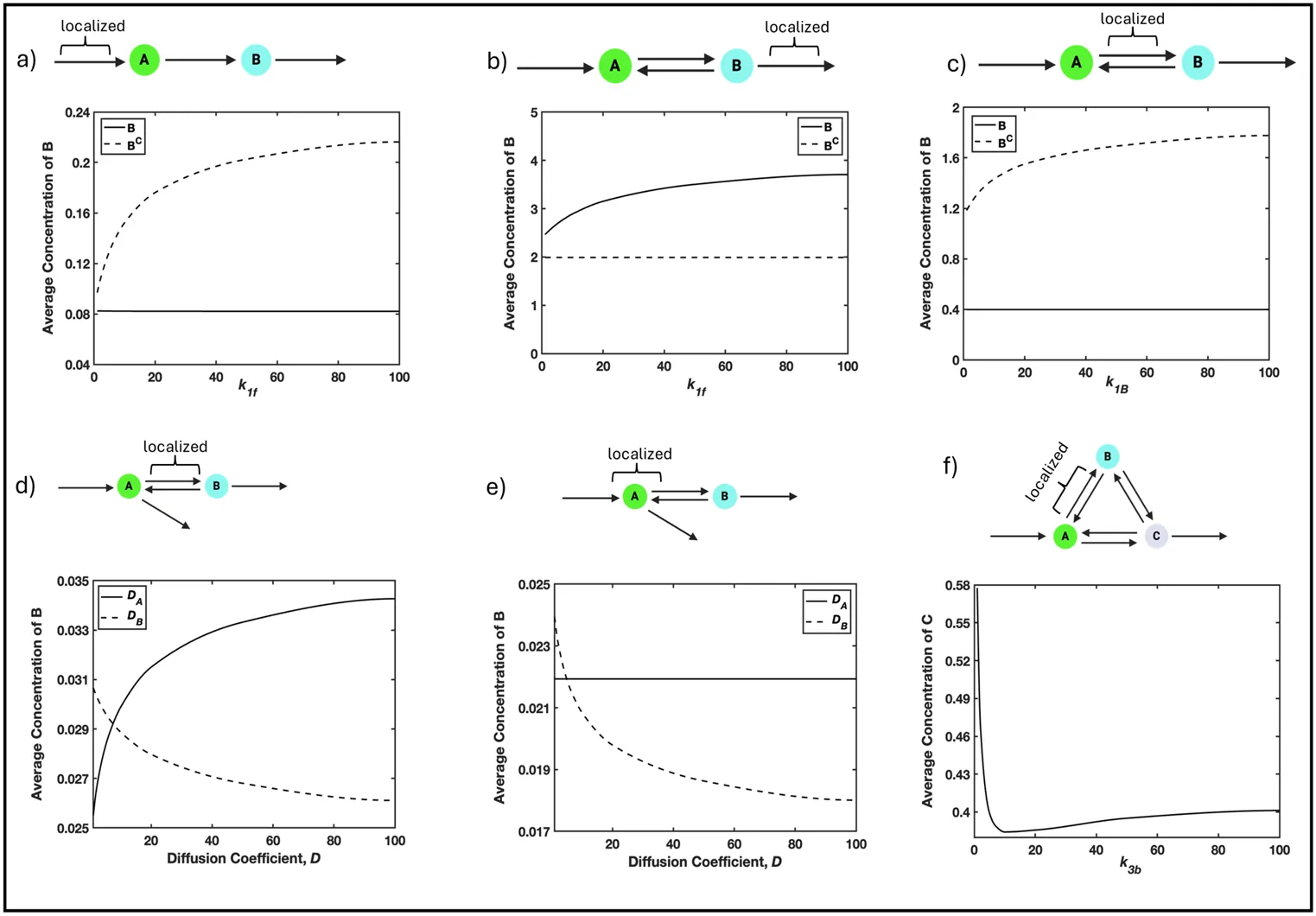
Behaviour of basic two-node and three-node motifs with localization of enzymes. The superscript c indicates a compartmental average [79]. In all cases, the relevant motif being investigated is depicted alongside the relevant plot. (a) A two-node, one-inflow one-outflow system, localized input to the system. The average concentration of B over the full domain (denoted B) and over the compartment (denoted $B^{c}$) is plotted for different values of the kinetic parameter for the A to B reaction. (b) The effect of varying the same parameter in a reversible two-node one-outflow motif where the localized species is the efflux enzyme of B. The average concentration of B over the full domain and over the compartment is plotted. (c) A reversible two-node motif with one outflow with localization imposed on the enzyme mediating the A to B reaction. The average concentration of B in the compartment is plotted for different values for the B to A reaction constant. (d) A one inflow two outflow two-node motif with reversible interconversion. The localization is imposed on the enzyme mediating the A to B reaction. The steady state of the average concentration of B is plotted for different diffusivities of A (solid) and B (dashed). (e) Similar plot and system to (d) but substrate A is the localized entity. (f) A three-node motif with one inflow and one outflow with a localization imposed on the enzyme mediating the A to B reaction. The compartmental average concentration of C is plotted for different values of the rate constant of the A to C reaction.

#### 3.1.2. Spatial localization can introduce unintuitive effects and kinetic dependencies which may not be inferred from kinetics alone

The next result (figure 2c) highlights how intuition from kinetics alone may be misleading. It presents an instance of an unintuitive result, wherein, for instance, increasing levels of enzyme responsible for the conversion of one species to another actually results in an increased average concentration of the first species over the sub-domain (though not over the full domain). This can be seen in a broad range of parameters in the basic case shown (two nodes, one outflow), where the enzyme responsible for conversion from species A to B is localized, and the enzyme level for the conversion from B to A is increased. However, a similar unintuitive result can be seen in the same motif, where the enzyme responsible for the B to A interconversion is localized, and the enzyme for the A to B conversion is increased, increasing the concentration of A. It can also be seen in three-node motifs with two outflows, where either an interconverting enzyme or the efflux enzyme is localized. Here, it is seen for parameter values which correspond to substantial perturbations of the two-node motif, and thus the behaviour is not a trivial echo of behaviour of the two-node motif. The fact that this unintuitive result is observed in different systems speaks to the essential robustness of the result. These results arise as a consequence of spatial localization: in appendix B, we show that the same kinetic systems without spatial localization (i.e. well mixed) are incapable of exhibiting this behaviour for any choice of kinetic parameters.

#### 3.1.3. Effect of species diffusivity, and dependence on choice of localized species

Figure 2d,e presents cases of the role of diffusivity, an invisible factor in purely kinetic studies of metabolic systems. Both cases consider the two-node motif with two outflows. We examine the effect of increasing the diffusivities of both A and B. In the first case, figure 2d, if the enzyme mediating the A to B reaction is localized, increasing the diffusivity of A leads to a progressively increasing spatial average of B, while varying the diffusivity of B has an opposite effect. On the other hand, if the substrate A is itself localized (figure 2e), increasing the diffusivity of A had a different effect on the output, keeping it practically fixed. This shows how the impact of species diffusivity on a motif depends on the identity of the species, and also on which species is localized.

#### 3.1.4. Species localization can introduce non-monotonic parametric dependence

We consider a three-node motif which is reversible with one outflow, and choose the enzyme associated with A to B conversion to be localized. We vary the concentration of enzyme associated with the A to C conversion and show that this affects the compartmental average of species C in a biphasic manner, exhibiting a clear minimum (figure 2f). Further explorations building on this reveal that the same behaviour can be observed if (i) an additional outflow is introduced or (ii) reversibility in interconversion is allowed only between the A and B reactions. In this latter case, the concentration of the enzyme associated with the B to A conversion is varied as a parameter. In appendix B, we show that such biphasic dependences are absent in corresponding well-mixed systems (irrespective of kinetic parameters). Biphasic responses represent an in-built competing effect, and we see how localization, even in very simple motifs, introduces the possibility of this, when it did not exist otherwise.

This unintuitive biphasic behaviour is expected to be present in neighbouring parameter regions from basic continuity arguments. We probed this further by varying three different interconversion rate constants over a significant range (including a fivefold difference in both directions) and found that the biphasic response was still present (in some cases accentuated or softened, but always present): see supplementary material, figure S2.

Our results highlight non-trivial qualitative behaviour, which emerges from the most basic considerations of spatial localization in basic motifs. One set of results arises primarily from the structure of the network (rather than parametric effects): an example is the maintenance of certain spatial averages, independent of many parameters, a structural invariant (also see figure S1 in the supplementary material). A consequence of the structural underpinning of the results is that they generalize to similar motifs with a larger number of nodes. The spatial average of the efflux is also fixed here, independent of interconversion rate constants. We note in passing that if the efflux is described by Michaelis–Menten kinetics (rather than mass action kinetics), then the structural results correspond to the maintenance of the spatially averaged efflux rates. Other results (biphasic dependence of output on kinetic parameters, unintuitive kinetic dependencies) occur as a result of localization and do not occur in well-mixed systems. The effect of changing diffusivity of a species needs to be assessed through the lens of the full reaction–diffusion network.

### 3.2. Basic motifs with feedback

The basic motifs considered previously did not contain any feedback regulation. The kinetic ODE models of such systems are characterized by a single stable steady state. We now consider two examples of feedback, wherein a substrate is able to indirectly promote either its own production or its removal.

#### 3.2.1. Two-node irreversible motif with one outflow

We first consider the case of a two-node motif (with no conversion from B to A) with one outflow (the species B), where B indirectly positively impacts the conversion from A to B (see appendix A). In a well-mixed system, depending on the strength of the feedback, the system is capable of exhibiting a steady state or sustained oscillations.

Studying the effect of spatial localization brings in new features into play: both the localization (and which species is localized) and the diffusivity of species. We first examined the effect of diffusivity, without the localization: this amounts to having a localization compartment corresponding to the full domain.

**Figure 3.**
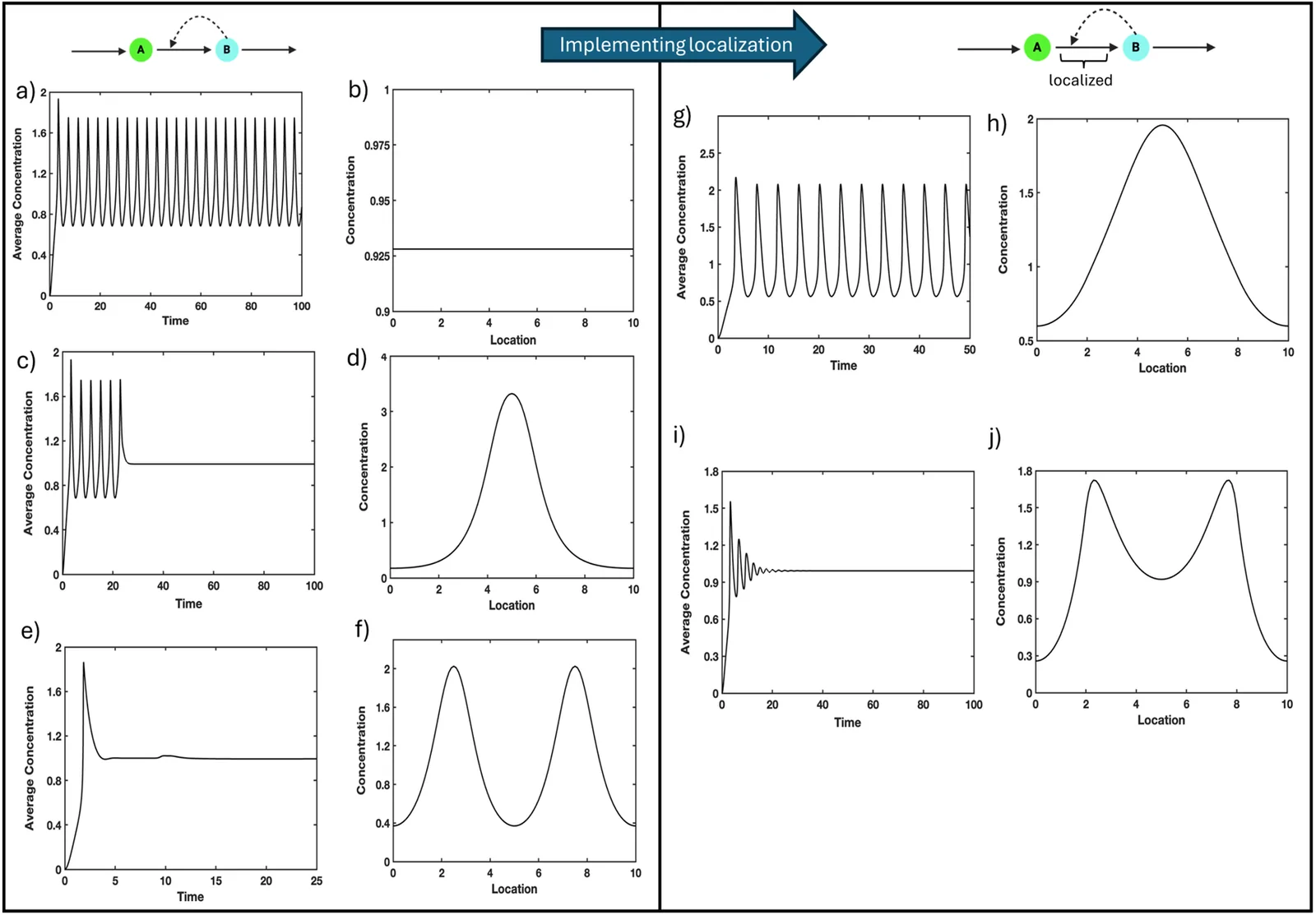
Different spatio-temporal behaviours for a two-node motif with feedback for different localizations and species diffusivities [80]. (a) The temporal behaviour of the spatial average concentration of B over the full domain for equal diffusivities of A and B and no localization. (b) The spatial profile of B at the final time point. This indicates that the system exhibits spatially homogeneous oscillations. Panels (c,d) consider the same previous system, with only one change: differing diffusivities of A and B. Here, a spatially inhomogeneous steady state is observed, even with no localization of any species imposed. Panels (e,f) depict the same system as in (c,d) but for the case of a larger feedback parameter. This leads to an inhomogeneous steady state with different characteristics. Panels (g,h) depict imposing a localization of the interconversion enzyme in a 60% compartment size for the case of equal diffusivities of A and B. Here, spatially inhomogeneous oscillations are observed (as seen in the spatial profile at the final time point, h). Finally, panels (i,j) examine the same case as (g,h) but for lower (equal) diffusivities. This change of diffusivities results in a multi-peaked inhomogeneous steady state.

#### 3.2.2. The emergence of spatial organization in the absence of imposed localization

We first consider equal diffusivities of both species (see figure 3a,b). We observed sustained spatially homogeneous oscillations which correspond to the behaviour of the kinetic ODE model. We then consider a case where the diffusivity of A is significantly greater than that of B. Here, we find a steady state which is spatially heterogeneous which is bell-shaped and symmetric about the midpoint (see figure 3c,d). Since there is no external spatial organization or localization imposed in this case, we conclude that this is an emergent pattern arising from the system interaction. In fact, this exhibits all the hallmarks of a Turing pattern (note that the basal state with equal diffusivity was that of spatially homogeneous oscillations, but the exact same outcome can be observed when we consider a kinetic regime, where the basal state was a homogeneous steady state). Since the nonlinearity is a key driver for the emergence of such a pattern, we then explore the impact of the feedback constant in this case. A 10-fold increase in feedback revealed that while the corresponding ODE behaviour was similar, the system again evolved to a spatially heterogeneous pattern, highlighted in figure 3e,f, which had a higher number of peaks. Incidentally, a similar effect can be observed by keeping the feedback constant fixed and varying the diffusivity of species A in a more moderate range. The two-node irreversible motif with one outflow is the simplest metabolic motif capable of a Turing pattern. Although feedback is a critical ingredient (from results of the previous sub-section), we see that the full-domain spatial average of the emergent pattern (of B) is independent of the feedback.

#### 3.2.3. Imposing localization can introduce multiple distinct behaviour

We explore the impact of localization, building on this. We have examined the impact of localization of both enzymes (interconversion enzyme, as well as efflux enzyme of B) and substrates (localizing species A) in this system, and will show the results for localization of interconversion enzyme, as the essentially new behaviour is observed here. To explore this systematically, we vary the extent of localization of the species ranging from 100% (no localization) to 20% of the full domain. We first examine the effect of spatial localization imposed on the motif when A and B have equal diffusivities and then consider different diffusivities. From a detailed analysis of these different cases, we obtain multiple distinct behaviours: (i) in figure 3g,h, where the interconverting enzyme is localized (60% of the domain) and the diffusivities of A and B are the same, we see a heterogeneous oscillatory pattern, with a spatial profile depicted at one instant of time; (ii) damped oscillations with a spatially heterogeneous profile essentially inherited from the localization (again equal diffusivities, but of lower magnitude compared to the previous case), see figure 3i,j; (iii) a highly dynamic spatio-temporal pattern, shown in figure 4a–d. This was observed in the case where the diffusivities of A and B are different, depicted in the instance of localization in 80% of the domain. Three snapshots of the spatial profile at different time instants are shown in figure 4b–d, showing transitions between symmetric and asymmetric double-peaked profiles. Interestingly, the spatial average concentration of B appears flat over broad ranges of time, punctuated by sudden bursts (see figure 4a and inset). (iv) An oscillatory system which involves a change of oscillation amplitude—starting with oscillations of a particular amplitude and the transiting to oscillations of a different amplitude (figure 4e–g). Figure 4e depicts the amplitude of oscillations for the spatial-averaged concentration of B, clearly demonstrating the change in the oscillation amplitude. Representative spatial profiles at instances in the higher amplitude and lower amplitude oscillation are shown in figure 4f,g. This is obtained for the case of unequal diffusivities of A and B and localization being 60% of the domain. Overall, we see that the interplay of localization and oscillatory kinetic behaviour can have different manifestations, and a systematic analysis can reveal the transitions between these distinct behaviours.

**Figure 4.**
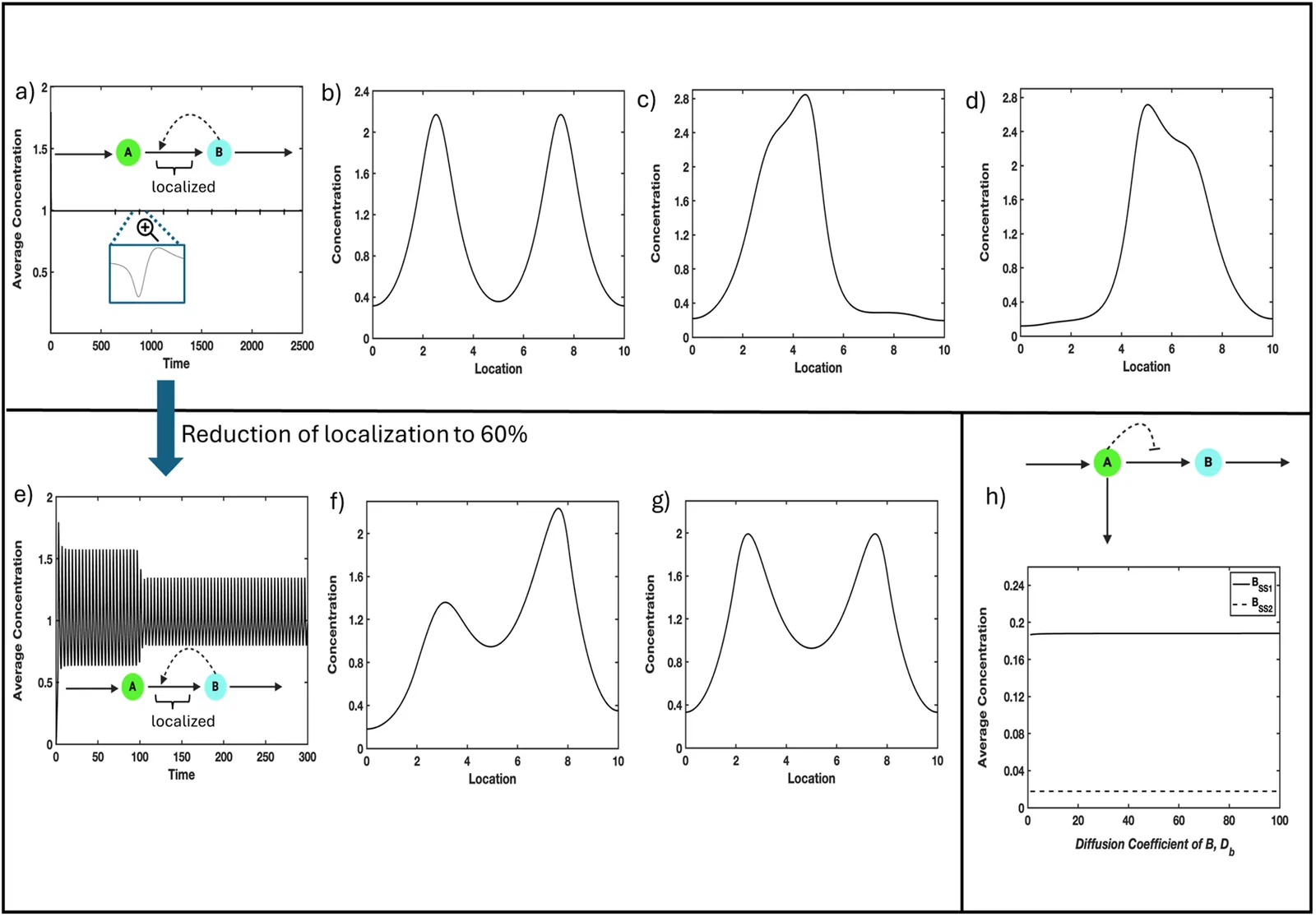
Different spatio-temporal behaviour for two different two-node motifs with feedback shown in (a,h) [81]. (a–g) Two-node motif with B promoting its production from A. (h) Two-node motif with A inhibiting its conversion to B. (a) The temporal behaviour of the average concentration of B over the full domain for the localization of the interconversion enzyme in 80% of the domain with highly contrasting diffusivities. The behaviour is constant for the most part, but punctuated by bursts (see inset). (b–d) The spatial profile of B at three different time instants, the final time (b) and two intermediate times (corresponding to bursts in (a)), (c,d) showing asymmetric behaviour about the middle of the domain switching from one asymmetry to the other. Panels (e–g) depict oscillations for a similar case as before, but for the case of a 60% localization with a lower diffusion coefficient of A (however still with contrasting diffusivities) and a lower feedback parameter. The spatial profile of B here is shown for two different time instants in (f,g). (h) The second feedback motif with localization of A in 20% of the domain showing two different steady states for the average concentration of B over the full domain.

#### 3.2.4. Two-node motif with species inhibiting its conversion

We now turn to a case where A inhibits its interconversion to B (see figure 4h). Here, A is converted to B irreversibly, but A and B can efflux. Here, altering the feedback constant can shift the system from having one stable steady state to two in the ODE. We first consider the case that the localized species is localized over the full domain. Here, with a wide range of computational studies, we did not encounter a heterogeneous spatial pattern even with significantly varying diffusivities.

We examined the role of both substrate and enzyme localization here. In the case of substrate localization (of species A, figure 4h), we can see multiple heterogeneous steady states (where A is homogeneous in its domain of localization, but B is not: this is possible since B is not converted to A). These different steady states are reached by varying the initial conditions. Localizing interconversion enzyme will result in multiple heterogeneous steady states for A and B. While localizing interconversion enzymes in certain instances (20% localization size), we computationally found only one steady state. However, increasing the enzyme concentration to keep the total amount fixed allowed us to access two heterogeneous stable steady states.

A single nonlinear interaction (feedback) can enable a range of qualitative behaviour/transitions. However, the nature of the feedback and identity of the species mediating the feedback are critical. In a two-node motif with irreversible interconversion and one outflow, A regulating its conversion to B (activating or inhibiting) does not generate complex behaviour like Turing patterns (the A system is decoupled from B here), while B regulating the same reaction can. The structural results of the previous section apply here as well, representing constraints on spatial and spatio-temporal patterns (see analysis in appendix B). For instance, a Turing pattern in a motif with one outflow species will have the spatial average of the species fixed (independent of feedback/interconversion). If spatial oscillations ensue (i.e. periodic in time but spatially inhomogeneous), the time average of the spatial average of the outflow species remains fixed (as can be inferred in figure 4h for both oscillations).

### 3.3. Control coefficients

A basic and widespread tool used for analysing, manipulating and engineering metabolic networks is the computation of control coefficients, which represent scaled logarithmic sensitivities of metabolite concentrations and fluxes to concentrations of enzymes associated with different reactions.

In the context of spatial organization and localization, we extend this approach from two essential perspectives: (i) the computation of control coefficients of spatially averaged fluxes or concentrations to enzyme concentrations (note that enzyme concentrations are constant in the region where they exist). This represents analogues of the standard kinetic control coefficients and is identical to them, in the absence of spatial organization (ii) the computation of control coefficients/sensitivities with respect to compartmental features, in particular the location (of the midpoint) of the compartment and the size of the compartment.

#### 3.3.1. Analogues of standard kinetic control coefficients

We first consider the analogues of the standard kinetic control coefficients, when localization is present. This addresses the question: what is the sensitivity of fluxes and metabolite concentrations, spatially averaged either over the full domain or a compartment, to enzyme concentrations? If localization is present, but not accounted for, and measurements are made by averaging over the full domain (e.g. lysing the cell) or in a specific spatial region, these are the control coefficients associated with the measurable outputs. Note that the region can be arbitrary (it does not have to the same region where localization is imposed), and by choosing a small enough size of the region, we can obtain the (scaled) sensitivity of concentration or flux at a particular location to changes to enzyme concentration. As part of the analysis, we also compute the sensitivity of species to diffusivities as well.

#### 3.3.2. Sensitivity to compartmental characteristics

When an enzyme is localized, we can consider control coefficients associated with the compartmental characteristics: either compartmental size (of a given compartment) or compartment (midpoint) location. In the case of compartment size, we can define a logarithmic sensitivity analogous to kinetic control coefficients. On the other hand, in the case of location, we need an appropriate scaled sensitivity measure. Here, a standard logarithmic sensitivity is not appropriate since the location variable may be positive or negative. Furthermore, any sensitivity must be invariant to shifts of the spatial coordinate system. Thus, here the control coefficient is defined as the derivative of the logarithm of the relevant output to a scaled dimensionless location (see appendix A).

When we examine sensitivity to compartment size, we consider two possibilities: (i) the enzyme concentration is fixed or (ii) the total enzyme amount is fixed, implying that the enzyme concentration changes in inverse proportion to the size. The latter sensitivity can be obtained from the former sensitivity coefficient and the kinetic sensitivity coefficient (see appendix A).

We have examined a range of two-node motifs, with or without reversibility in the interconversion, with one or two outflows (i.e. both A and B have outflow). We have also examined a selection of three-node motifs with one or two outflows. We present a selection of some basic results here. Localization of different enzymes involved either in interconversion or in outflow has been considered.

**Figure 5.**
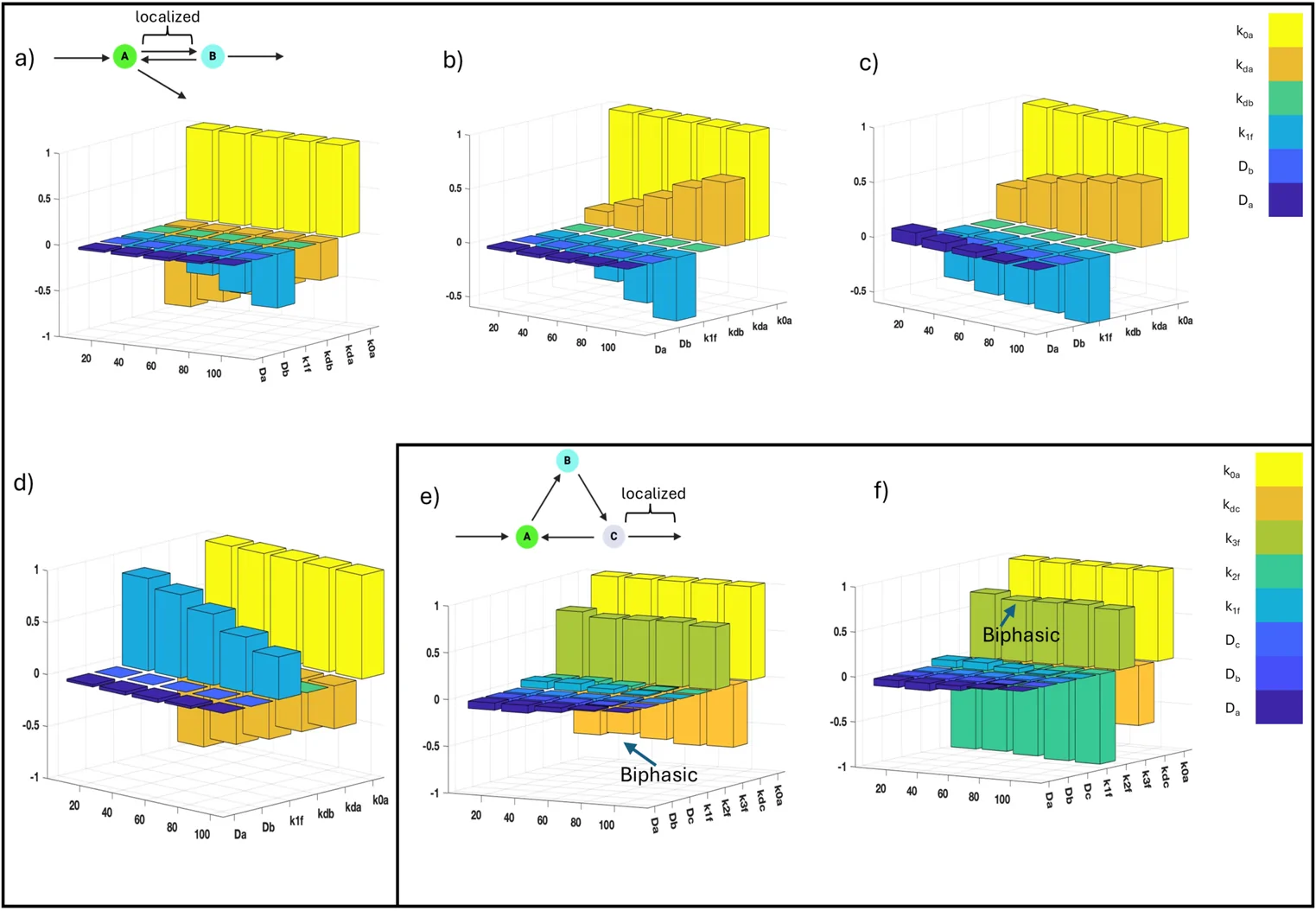
Control coefficients for fluxes and concentrations in systems with spatial localization. Panels (a–d) represent a two-node one inflow two outflow system with localized interconversion, while (e,f) represent a one input one output cyclic three-node system with localized outflow. All six panels are 3D plots which show the sensitivity of the given flux or concentration to all the kinetic parameters and the diffusion coefficients for different compartment sizes. Panel (a) depicts the full domain-averaged concentration control coefficient of species A. Panel (b) shows the full domain-averaged flux control coefficient for the flux associated with the efflux of A. Panel (c) is similar to (b) but evaluates the compartment-averaged flux control coefficient. (d) Full domain-averaged flux control coefficient of the A to B reaction. (e) Full domain-averaged flux control coefficient for the B to C reaction of the three-node system. (f) Full domain-averaged concentration control coefficient for B [82].

Figure 5 considers the sensitivities of spatially averaged fluxes or concentrations to kinetic constants (a proxy for associated enzyme concentrations) in both two-node (figure 5a–d) and three-node (figure 5e,f) motifs. The concentrations and fluxes are averaged either over the full domain or over the specific compartment (region of localization). In a single plot, we have shown the control coefficients and how they vary with the size of the localization of the entity being localized. A 100% localization corresponds to the case of no localization, in which case, the control coefficients become the standard control coefficients of the kinetic model. Figures S3– S5 in the supplementary material present additional associated data, including 2D projections.

#### 3.3.3. Species localization can have a significant impact on kinetic control coefficients

Figure 5 examines the case where the extent of localization is varied keeping the enzyme concentration fixed. Here by examining the various results, a few basic insights emerge. (i) Firstly, the magnitude of the control coefficients can depend on the size of the localization, something seen in essentially every case. (ii) Interestingly, in some cases, the relative ordering of the magnitude of the control coefficients is altered (e.g. see the relative magnitude of the light blue bars and orange bars in Figure 5a). This implies that which is the more sensitive rate constant (or associated enzyme) can depend on the size of the localization. (iii) In many instances, the control coefficient is a monotonic function of localization size. In some cases, for instance, in figure 5e, we find that a flux control coefficient can actually vary biphasically with specific enzyme/kinetic factors (orange bar), indicating that there are intermediate localization sizes, which exhibit the highest sensitivity for this parameter. In a few cases, the dependence of sensitivity coefficient on the size of localization is very weak. The biphasic behaviour observed in the flux control coefficient continued to hold good when other parameters were varied.

When we change the localization size keeping total amount of enzyme (localized) fixed, all the above features can still be observed. This only reinforces the types of impact spatial localization can have on control coefficients, and demonstrates that this can occur with or without a change in total enzyme amount.

In many instances in analysing metabolism, purely kinetic or flux-based analysis is used, citing the fact that spatially resolved data may not be available. However, as our results show, the standard control coefficients exhibit important and sometimes unexpected dependence on the size of localization even if implicit, and consequently suggest that in these instances, the use of these control coefficients must be made with caution.

#### 3.3.4. Sensitivity to compartmental size and location

We now turn to control coefficients directly associated with localization characteristics. To do this, we focus on a two-node motif with one outflow and irreversible interconversion, and consider one compartment and examine the sensitivity to two characteristics: (i) the size of the compartment (figure 6, table 1, scenarios 3–5) and (ii) the location, which is represented by the coordinate of the midpoint of the compartment (figure 6, table 1, scenarios 1 and 2). In each scenario of table 1, we assess sensitivities of different measures: either domain-averaged or compartment-averaged concentration or flux. This is repeated in table 2 and supplementary material, figure S6, to span a range of two-node motifs, to examine the effect of the kinetic network structure.

**Figure 6.**
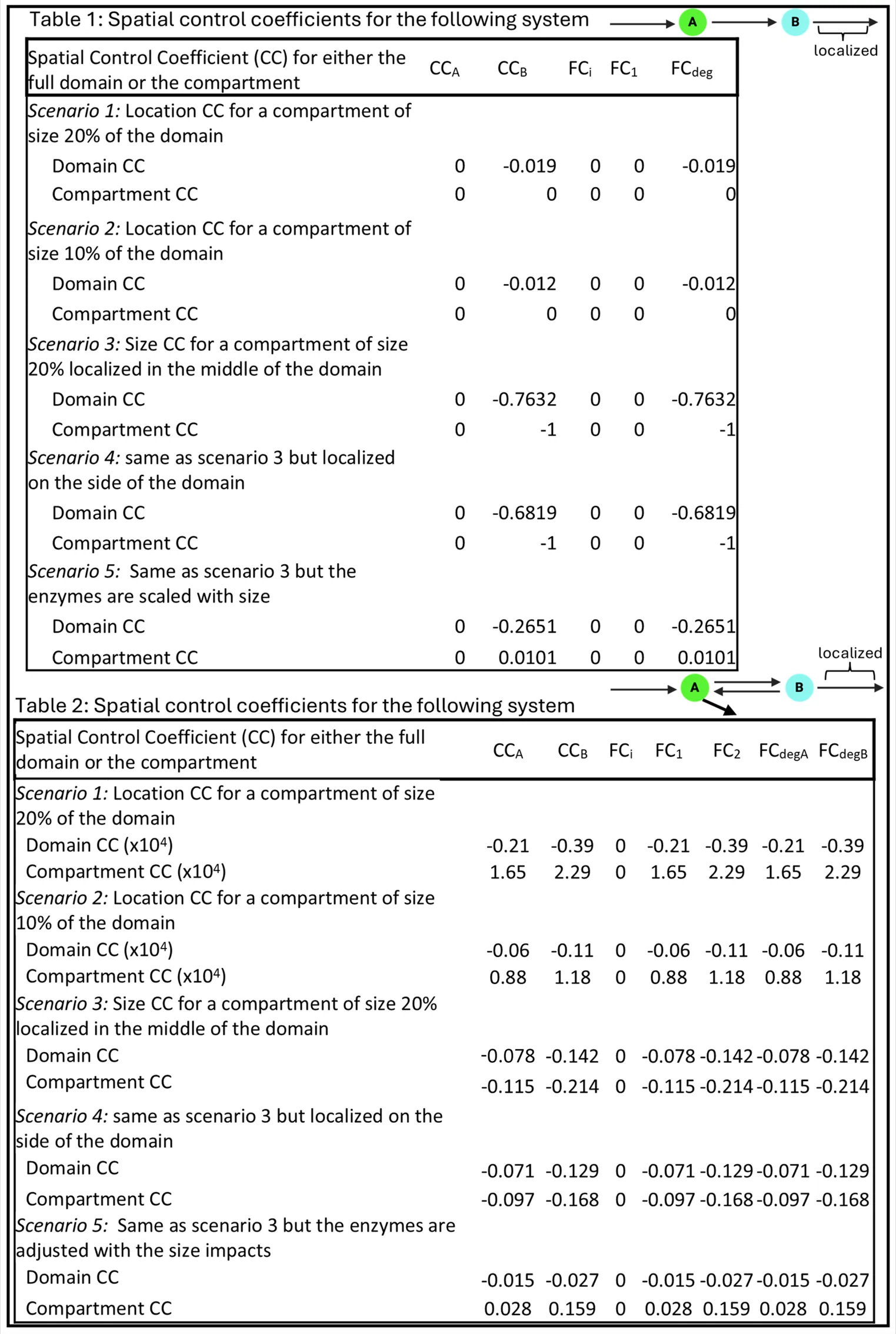
Tables 1 and 2 in the figure present the spatial control coefficients for the location and the size of compartment for two-node one inflow one outflow irreversible motif and two-node one inflow two outflow reversible motif, respectively (see text for details). In each table, five different scenarios are presented. Two of them (1, 2) show the location control coefficient for two different sizes of the compartment (note the different scales for these respective entries in tables 1 and 2). The subsequent three (3–5) depict different size control coefficients. Scenario (3) is based on a compartment centred in the middle of the spatial domain. Scenario (4) is based on a compartment asymmetrically placed with respect to the centre of the domain. Scenario (5) evaluates the control coefficient for the case of scaling the concentration of enzymes inversely with the size of the compartment to keep total amount fixed. For each scenario, two rows depict the cases where the output measure (whether concentration or flux) is averaged either over the full spatial domain (Domain CC), or over the compartment (Compartment CC). The different columns denote the specific output (concentrations of specific species or fluxes of particular reactions): $CC_{A},CC_{B}$ denote the control coefficients for concentrations of A and B, while $FC_{i},FC_{1},FC_{\deg}$ denote coefficients associated with the influx to A, the A to B conversion and the efflux of B, respectively. Additionally, $FC2,FC_{degA},FC_{degB}$ in table 2 denote coefficients associated with flux from B to A, efflux of A and efflux of B, respectively [83].

When we consider the size of the compartment, we find that certain fluxes and concentrations (either averaged over the full domain or a single compartment) exhibit clear and in some cases significant sensitivity with respect to size (figure 6, table 1, scenarios 3 and 4, columns 2 and 5). Furthermore, if the total enzyme amount is kept fixed, then the size control coefficient can even change sign (figure 6, table 1, scenario 5 when compared with scenario 3, column 2). On the other hand, we found that the control coefficient with respect to location was generally small (figure 6, table 1, scenarios 1 and 2; table 2, scenarios 1 and 2 (note the different scale here)). We note in these results that certain control coefficients are identically zero, and discuss this below.

#### 3.3.5. The kinetic network structure leaves a specific imprint on the spatial control coefficients

We dissect these aspects in more detail. The results in figure 6, table 2, are shown for a two-node motif with one inflow and two outflows and reversible interconversion. As expected, all the control coefficients are generally non-zero, except those associated with the influx (column 3). The latter result is because influx does not depend on localization characteristics here. In order to systematically understand how these control coefficients depend on the motif characteristics, we consider a sequence of two-node motifs (with one inflow) with increasing complexity: (i) irreversible interconversion between A and B, and only one outflow: B (shown in figure 6, table 1); (ii) reversible interconversion between A and B and one outflow; (iii) irreversible interconversion between A and B and two outflows (see figure S6 in the supplementary material for cases (ii) and (iii)); and (iv) reversible interconversion between A and B and two outflows (shown in figure 6, table 2). In each case, we examined the localization of the efflux of B. Examining each of these cases pinpoints special values of specific control coefficients.

We start with the one outflow case with irreversible interconversion between A and B. Table 1 in figure 6 reveals some characteristic features. We find that control coefficients associated with the concentration of A (column 1) or the flux from A to B (column 4) are zero whether with respect to localization size or location. This can be explained analytically. Examining the equation for A, we find that the steady state of A is spatially uniform and not affected by the efflux of B. Hence, these various control coefficients are zero (some of these control coefficients become non-zero when the A to B reaction is localized, because localization features impact A). The control coefficients associated with the compartment average of B (with respect to location of compartment) are also zero (column 2). This is because at steady state, net inflow to the system equals net outflow. A consequence of this is that the average concentration of B in the compartment is fixed. Now if the size of the compartment is changed, for the very same reason, the average concentration of B in the compartment varies in inverse proportion to the compartment size, resulting in a size control coefficient of −1 (column 2). Since the total inflow equals the flux from A to B, the latter is independent of the compartment size and location, and this results in zero control coefficients for this flux (see column 4). By progressively reducing the restrictions on the motif and considering other scenarios, we find that individual zero control coefficients can become non-zero (shown in figure S6 in the supplementary material). This allows us to systematically bridge the divide between figure 6 (tables 1 and 2). By studying all these cases, we obtain insights into how network structure impacts spatial control coefficients.

#### 3.3.6. Effect of one compartmental characteristic on sensitivity with respect to the other

We also examined how each spatial control coefficient depends on the other spatial factor. We found that the size control coefficient had a weak dependence on location, as seen in figure 6 (table 1, scenarios 3 and 4). The location control coefficients showed some dependence on size only when the localization size was small (figure 6, table 1, scenarios 1 and 2).

The (analogues of) kinetic control coefficients can be strongly altered by the species being localized and the extent of localization. It can even change the identity of the most sensitive reaction rate constant/enzyme. This is true both when the enzyme concentration in the compartment is kept fixed and when the total enzyme amount is fixed. When the total amount of enzyme is kept fixed, the associated size control coefficient can change sign indicating that there is an intermediate localization size, which represents a maximum/minimum for the output under consideration (also see analysis in appendix B). The control coefficients can be used to estimate the sensitivity of changing more than one factor simultaneously (e.g. size and location of a compartment, or the size of multiple compartments). Finally, our structured approach to assessing control coefficients proceeding from simpler to more complex motifs allows us to bridge the divide between purely structural insights, and purely control coefficient-based analysis.

### 3.4. Spatial localization from an engineering perspective

One of the key focal points in the analysis of metabolic networks is that of engineering. Here, we revisit metabolic networks with spatial localization from this perspective. Engineering of these systems is pursued with a typical goal of maximizing or minimizing metabolite concentrations or fluxes. At the outset, when one considers spatial localization in metabolic networks, there are different aspects which could be the focal point for engineering: the choice of the network itself (for instance, from a suite of candidate networks), the choice of species to localize and the compartmental features (size, location, number). All of these features could, in principle, be subject to a simultaneous variation for the purposes of optimizing outcomes. In this instance, we will focus on a restricted but more natural set of factors and how they are varied. To start with, the network is fixed: we will analyse a sample set of networks (one two-node and two three-node motifs). We will focus on a particular sequence in the engineering: first, the most promising species (substrates or enzymes) are selected for localization. This is based, for instance, on a nominal localization scenario with specific compartmental characteristics. Following this, the compartmental characteristics can be varied. We consider three network motifs with two outflows: a two-node reversible motif and two three-node motifs (one irreversible and one with reversible interconversion between species).

#### 3.4.1. Effect of choice of localized species

We first start by evaluating the impact of localization of different enzymes. For specificity, we localize the enzyme in a compartment whose size is 20% the size of the full domain, centred at the middle. Figure S7 (tables S3– S5) in the supplementary material demonstrates the impact of localization of each enzyme in each of these networks. The tables have two axes: one axis (vertical) corresponds to different choices of the enzymes which are localized, and the other (horizontal) axis corresponds to different potential output measures: the spatially averaged concentration of different species, either averaged over the compartment (the localization compartment) or over the full spatial domain. Table S3 in the supplementary material already reveals multiple salient points: localizing the efflux enzyme of species A is the only case which leads to an increase in all measures of the output. Localizing the efflux enzyme of B exhibits an overall different pattern of impact when compared to localizing the degrading enzyme of A. Furthermore, localizing each of the enzymes associated with interconversion of A and B results in an increase in only one measure (the spatial averaged concentration of the species on which the enzyme is acting), while leading to a reduction in the other three output measures.

#### 3.4.2. Trade-offs in localizing species

Tables S4 and S5 in the supplementary material show many instances where localizing a species leads to trade-offs: either some species’ average concentrations increase and others decrease (typically observed) or the same species domain average and compartment averaged concentration exhibit opposite trends. Thus, a simultaneous increase of different combinations of species concentrations is not achieved in these cases. Furthermore, some combinations such as a reduction of A and B and an increase in C (domain averaged concentrations) are not found in any of the cases in table S4 in the supplementary material. On the other hand, localization can be a fundamental enabler: for instance, in table S4 in the supplementary material, localizing the enzyme responsible for interconversion of A to B leads to an increase in all measures, something nominally not possible by a perturbation of any of the enzyme concentrations in well-mixed systems.

#### 3.4.3. Manipulating and designing compartmental characteristics

Figure S7 (tables S6– S9) in the supplementary material explores the potential impact of varying compartmental characteristics for the two-node network (tables S6 and S7) and the three-node reversible network (tables S8 and S9) considered above (note that in tables S8 and S9, only a selection of enzymes is depicted for conciseness, and that the horizontal and vertical axes are swapped). Two characteristics of compartments are considered (i) a change of location and (ii) splitting a compartment into two and placing them symmetrically about the middle of the domain. A few points can be immediately observed. Firstly, none of the cases studied (for the nominal parameter set) resulted in an increase in all measures of the output when enzymes are localized (this is true for all enzymes, including those not depicted). The simultaneous localization of substrates B and C in table S8 in the supplementary material could however achieve this. Secondly, while some measures increase and others decrease, the exact pattern (of which measures increase and which decrease) significantly varies, depending on which species is being localized. Changing the location affects the compartment average and domain average of each species in opposite ways in the two-node motif (supplementary material, table S6). Splitting the compartments has, in almost all instances, the exact opposite effect as changing the compartment location (supplementary material, tables S6 and S7). Similar effects are seen in a number of instances in the three-node motif (supplementary material, tables S8 and S9).

#### 3.4.4. The effect of splitting a compartment into multiple compartments depends on sizes and locations of compartments

Figure S7a in the supplementary material considers the impact of multiple localizations on a two-node motif in the following way. Four different cases are considered together: (i) the baseline compartment structure; (ii) breaking the compartment into two halves and placing it symmetrically about the middle of the domain; (iii) using two compartments of the same size as the original compartment, with the original total amount of enzyme equally distributed between the two compartments; and (iv) the same case as (iii) but with an unequal enzyme distribution. As can be seen from the plot, all the cases differ from one another, and in fact all cases (ii–iv) result in a smaller measure of output when compared to (i). This shows how splitting a compartment (or even using more compartments), as well as altering the distribution of enzymes between compartments can significantly impact the outcome.

#### 3.4.5. Trade-offs and their classification

Our results in this sub-section combined with earlier results point to the presence of multiple trade-offs associated with localization. In each instance, we consider the variation of a parameter/factor. The trade-offs can be classified along two axes: one which indicates whether the trade-off is reflected in either one output or two outputs. The other is the nature of the parameter/factor, whether kinetic or spatial. For each combination of instances, it is possible to obtain a trade-off, which was not present in the well-mixed kinetic system with mass-action kinetics (irrespective of parameters). This sharply delineates the introduction of a trade-off associated with spatial localization. The fact that varying a kinetic parameter could give rise to a biphasic effect on certain outputs (see figure 2f) is an example of a single output trade-off associated with a single kinetic parameter. We establish that this trade-off (and biphasic dependence) does not exist in the well-mixed kinetics system (see appendix B). The fact that varying the size of a compartment keeping the total enzyme amount fixed results in a biphasic dependence on a single output (see §3.3) is an example of a single output trade-off associated with a spatial factor. There are multiple examples of the variation of a kinetic factor giving rise to combinations of variations of two outputs which may not be desired. One example is the unintuitive behaviour discussed in §3.1) (where we discuss that this is absent in the well-mixed kinetic system). Another instance of this is seen in a three-node motif (supplementary material, figure S1b), where the variation of compartment and spatial average of a species vary in opposite ways when a kinetic parameter is varied. An example of a spatial factor giving rise to trade-offs in two outputs is seen in the effect of introduction of localization on two different output measures: the compartment average and spatial average of a species (see supplementary material, figure S7, tables S3– S5). Another is seen in table 2 (scenario 5). We see that the domain and compartment averages of some species vary in different ways. These cases represent a trade-off if it is desired to increase (or decrease) both measures of output simultaneously. This trade-off absent is in the well-mixed system, as both measures behave identically there. Finally, some of the above examples also demonstrate how some basic kinetic constraints may be circumvented by spatial localization: for example, unintuitive dependence on kinetic parameters (§3.1) or having one measure of concentration (either compartment average or domain average) behaving opposite to how that particular species concentration varies in response the parameter in the purely kinetic system.

### 3.5. Exemplar case

In this sub-section, we complement our earlier bottom-up analysis of network motifs with a top-down analysis of a concrete exemplar case of glycolysis. The associated network is depicted in figure 1. For this purpose, we employ a model in the literature [52] of the relevant pathway, which is calibrated against experimental data. This ODE model of the kinetics has been expanded into a PDE model, and our focus is on spatial localization of enzymes. We again use a 1D spatial model for simplicity and incorporate diffusion of different species. Our analysis focuses on assessing the effect of localization of a selection of different enzymes, one at a time. This is built upon by examining the effect of the localization of two enzymes.

#### 3.5.1. Different effects of localization emerge from localizing different enzymes

We first focus on spatial localization of a key enzyme in the main pathway: PFK. The localization occurs in a compartment in the middle of the domain (whose size is 20% of the size of the domain). A basic insight which emerges is the reduction of steady-state concentration of all the species downstream. This is in line with trends seen in simpler motifs earlier. The impact of species diffusivities was assessed by varying the diffusivities of the species (all assumed equal for simplicity), shown in figure 7a,b. A general observation is the increase in spatial average concentration of these downstream metabolites, though there is a saturation effect for higher diffusivities. Incidentally, this is true of the (spatially averaged) concentrations in the compartments as well.

**Figure 7.**
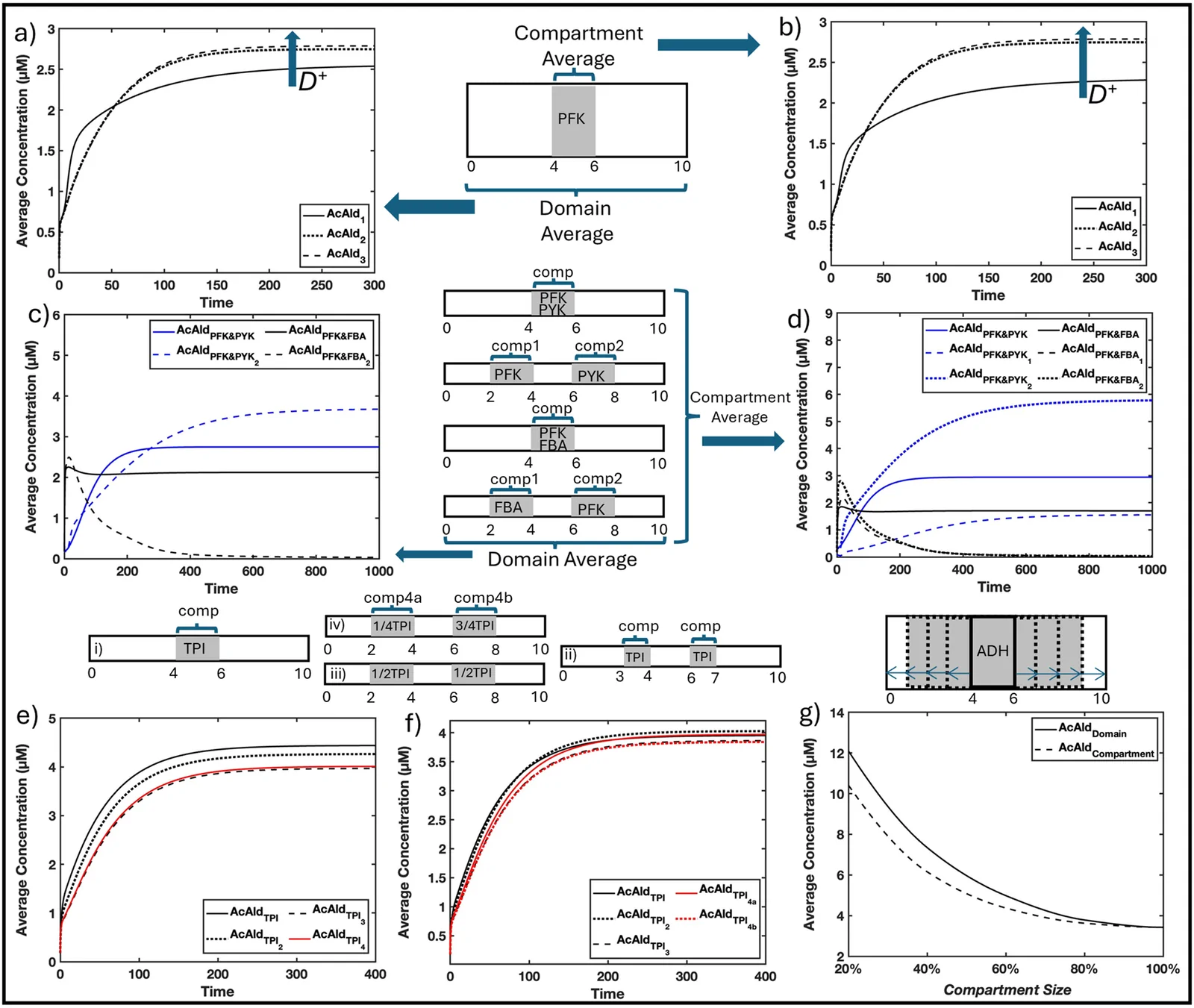
Evaluation of the concentration of acetaldehyde, AcAld, spatially averaged over the full domain and the compartment for different localization scenarios. Panels (a,b) evaluate the effect of the diffusion coefficient of the species (assumed equal) on the temporal behaviour and steady state for the case of PFK localization. Panel (a) represents the domain-averaged concentration of AcAld, while (b) represents the compartment-averaged concentration of AcAld. Panels (c,d) highlight the behaviour of the domain-averaged and compartment-averaged concentration, respectively, for two different cases of localizing two enzymes in the system (PFK, PYK) and (PFK, FBA). In the domain average, a subscript 2 indicates that the enzymes are localized in different compartments. In the compartment averages, the numeral subscripts (either 1 or 2) indicate which compartment (average) is evaluated in the case the enzymes are localized in two compartments. A schematic depiction of the compartments is presented in between (c) and (d). (e,f) Effects of different concentration distributions of TPI localization on the spatial average concentrations over the domain and the compartment, respectively. The cases are as follows (and subscripts in the legends correspond to these scenarios). (1) TPI basal in the middle with a size of 20% of the domain. (2) TPI localized in two compartments half the size of the original, placed symmetrically in the domain each with the same enzyme concentration of the basal (this fixes the total amount of enzyme). (3) TPI localized in two compartments that are of the same size as the original, placed symmetrically in the domain, however each with half the concentration of TPI compared to the basal case (the total amount of enzyme is fixed). (4) Same as (3) but the amounts of enzymes are distributed differently. Compartment 1 contains 25% of the total amount of TPI, identified in (f) with subscript a. The remaining 75% is the second compartment, identified in (f) with subscript b. The subscripts a and b are indicated in the legends for the compartment averages in scenario 4 (in scenario 3, the compartmental averages are the same, and represented by a single curve). Panel (g) presents the impact of the compartment size on the domain and the compartment average when ADH is the localized enzyme.

We then examine another enzyme (ADH) in a similar fashion. ADH represents the efflux enzyme of the output. As expected, localization of this enzyme increases the steady-state output (figure 7g), and this is reflected in the increase in spatial averaged output concentration, with reduction in the compartment size (this is true for both spatial averaging over the full spatial domain and spatial averaging over the compartment). This behaviour mirrors results seen in simpler motifs previously. Interestingly, when evaluating the impact of changing the size of the compartment, even scaling the enzyme concentration, so as to keep the total amount of enzyme fixed, the spatially averaged steady-state output changes as depicted in figure 7g.

A distinct feature of this system, not encountered previously, is the presence of enzymes which are bifunctional. We therefore examine the effect of spatial localization of the bifunctional enzyme TPI (figure 7e,f). As in the previous sub-section, we consider four cases: (i) the baseline compartment structure; (ii) breaking the compartment into two halves and placing it symmetrically about the middle; (iii) using two compartments of the same size as the original compartment, with the original total amount of enzyme equally distributed between the two compartments; and (iv) the same case as (iii) but with an unequal distribution.

We find that the baseline compartmentalization/localization increases the spatial average of the output, relative to no localization figure 7. All cases (ii–iv) result in a smaller measure of output when compared to (i). This shows how splitting a compartment, as well as altering the distribution of enzymes between compartments, can significantly impact the outcome in this concrete case mirroring results seen previously in the context of motifs. We note that the compartment average does not follow the same relative ordering of output concentrations: case (ii) involving splitting the compartment results in the highest compartment average, followed by the case of asymmetric distribution of enzymes (for the compartment with less enzyme), both of which are higher than the basal localization in scenario (i).

#### 3.5.2. Localization of multiple enzymes

We now consider the case of multiple enzymes localized in one or two compartments. We examine the localization of two enzymes, PFK and PYK, which are enzymes on the same central branch. We first localize both enzymes in a single compartment. While PFK localization alone reduces the steady-state average output (relative to no localization), localization of both enzymes in fact increases the steady-state average output, indicating that PYK localization dominates the effect of PFK localization and completely reverses the localization trend (not shown). This was also observed by localizing the enzymes separately in different compartments. We find that the domain average concentration of output is higher when the enzymes are in separate compartments, as opposed to the same compartment. By contrast, the compartment that localizes PYK generated the highest compartmental average (figure 7c,d).

We examine localization of two enzymes, PFK and FBA, that regulate consecutive reactions. Here, we consider two cases: (i) they are both co-localized in the same compartment and (ii) they are localized in two different compartments of the same size as the original but placed symmetrically about the middle of the domain, similar to what was done in the previous case (shown in figure 7c,d). When the enzymes are in the same compartment, the steady-state average output is reduced, relative to the case of only PFK localization (and based on the above results, of the case of no localization). This indicates that PFK and FBA localization affects the system in a similar way. Additionally, the system becomes spatially uniform outside the compartment, a behaviour seen in the simpler systems (not shown). When the enzymes are localized in two different locations, the steady-state output becomes much smaller indicating a significant attenuation of the output (dashed dark line in figure 7c).

## 4. Discussion

There has been a significant shift in interest in spatial organization in cell biology, from a peripheral and largely ignored topic, to a subject where interest is building rapidly. The interest in spatial biology spans systems and synthetic biology, and has been catalysed by various tools (imaging, spatial proteomics) [53–55], creation of synthetic compartments in synthetic biology [56–60], and the advent of membraneless compartments [61–63]. Notwithstanding this significant increase in interest, there are basic issues regarding spatial organization, which are not well understood. In this paper, we took basic steps towards addressing the basic question: how does spatial organization (in particular localization) impact metabolic systems? While there are experimental studies focusing on building artificial compartments in specific metabolic systems [56–60] and other studies focusing on certain aspects of spatial organization at the cellular and sub-cellular level in specific contexts [64–71], a broad-based understanding of this issue is largely missing.

We note, at the outset, that aiming to address aspects of this question involves recognizing the multiplicity of systems, the different ways in which localization is present, the key focal points for data analysis and the variety of contexts where this is relevant. This informs the approach we take. Addressing this question involves examining details and features of networks on the one hand, and assessing the effect of spatial factors on the other, while isolating recurrent aspects of their interplay. We develop a multi-pronged approach along and across different axes. (i) Systems: spanning basic two-node and three-node motifs, motifs with feedback and concrete examples. (ii) Focal points of analysis: spatially averaged concentrations (and fluxes) and qualitative dependence on parameters on the one hand, and control coefficients on the other. (iii) Contexts: spanning spatial engineering in synthetic biology, and investigating concrete exemplars (glycolysis) in systems biology. The two- and three-node motifs are building blocks of more complex systems and were described using mass-action descriptions of enzymatic reactions (see appendix A), ensuring that basic trends uncovered are probably to be broadly observed. A detailed study of motifs provides transparent insights which transcend an individual context and can be used for understanding and engineering specific systems. This is complemented by a detailed model calibrated against experimental data. This dual approach allows us to isolate and pin down key drivers of behaviour, engage with the details of concrete systems and provide insights spanning systems biology, synthetic biology and cell-free systems.

In contrast to signalling, the focus in studying metabolic networks is less on qualitative and more on quantitative aspects. This is because the propensity for and observance of rich qualitative behaviour is generally less than in signalling networks. Our approach is characterized by the feature that it repeatedly seeks the ‘qualitative in the quantitative’, aiming to uncover qualitative features in quantitative results: notable trends, alteration in kinetic trends owing to localization, unexpected features owing to changes in spatial factors and unexpected contrasts with the same kinetic system under well-mixed conditions (i.e. ODE models). The same approach extends to our analysis of metabolic control coefficients. In so doing, we reveal insights and trends which are unexpected and are not restricted to a particular choice of parameters. The fact that some of these behavioural trends are echoed across motifs and in the detailed exemplar case speaks to the robustness and utility of the conclusions.

### 4.1. Exploration of basic motifs reveals qualitative features associated with spatial localization

We uncovered various qualitative trends when spatial localization (of either enzymes or substrates) was present. In some motifs, with localization of certain species, spatial averages (either over full domain or compartment) of select species and fluxes may be fixed, independent of most kinetic parameters and spatial parameters. This points to a key structural feature in the model (the feature is independent of parameters) and can be explained analytically. We find multiple effects which cannot be seen in well-mixed systems of the same kinetics (irrespective of parameters): (i) unintuitive effects of varying enzymes on species concentrations and (ii) non-monotonic dependence of certain outputs (spatial averages of concentrations) on kinetic parameters. The effect of varying the diffusivity of a species in a motif, with given kinetic parameters, can strongly depend on which other species are localized: changing the identity of the localized species can result in qualitatively different trends. This underscores the need to assess the effect of species diffusivity through the lens of the network of which it is a part.

### 4.2. Networks with feedback

Even in the absence of localization, a spatially heterogeneous steady state can be obtained. This occurs when the diffusivity of substrates is sufficiently different and is characteristic of a Turing instability [72]. Altering the feedback strength can alter the characteristics of the heterogeneous steady states. The two-node irreversible feedback motif with one outflow is the simplest metabolic motif, which can exhibit a Turing pattern. In this two-node motif, there are structural restrictions on which species can mediate feedback to realize this. Further, the pattern which emerges is constrained (in spatial average) to be independent of feedback. Exploring the effect of localization of both substrates and enzymes here reveals a range of behaviour from oscillations reflecting the spatial characteristic of localization, to highly dynamic spatio-temporal oscillations involving a switching between different spatial characteristics. In other feedback motifs, localization can result in the possibility of multiple heterogeneous steady states with the possibility of switching between steady states.

### 4.3. Control coefficients

Metabolic control coefficients (sensitivities of fluxes or species concentrations, to concentrations of enzymes or kinetic parameters) are used to study metabolic networks and also to determine which enzymes to target to best improve outputs. In considering spatial localization, we focus on two aspects. The first is the computation of ‘standard’ metabolic control coefficients when spatial localization of enzymes is present. Thus, we compute the sensitivity of spatially averaged (either over a compartment or the full domain) concentrations or fluxes to changes in kinetic parameters or enzyme concentrations. We find that localization can alter both absolute and relative magnitudes of the control coefficients. The control coefficients can depend on the degree of localization in a non-monotonic manner. Thus, actual control coefficients in a system with spatial localization may vary significantly (and exhibit very different trends) from those computed from an ODE model. We then introduce control coefficients which quantify the sensitivity of (spatially averaged) fluxes and species concentrations to both sizes and positions of compartments/localization and investigated these coefficients. We consider two scenarios, for varying localization, one where enzyme concentration is fixed, and the other where enzyme amount is fixed, and observe broadly similar results in both instances. We find in some instances that the size control coefficient can even change sign: this occurs when the size of the compartment is varied keeping the total amount of localized enzyme fixed. This indicates that there may be optimal compartment sizes for certain output measures. The sensitivity measure for the location of a compartment differs from the usual logarithmic sensitivity, as the logarithmic sensitivity is not translationally invariant or generally well defined.

### 4.4. Engineering localization

Using two- and three-node motifs as illustrative cases, we explored in sequence (i) the impact of the choice of species to be localized, (ii) the impact of compartment characteristics, and (iii) splitting compartments and distribution of enzymes in multiple compartments. These studies reveal a number of insights. For instance, the localization of certain enzymes could affect different desirable outputs in a similar way, while the localization of other enzymes can result in a trade-off, impacting some outputs favourably and others unfavourably. In some cases, localization could impact two different spatial averages of a species (for instance in a compartment, and over the full domain) in opposite ways. The effect of the location of the compartment, the splitting of a compartment into two compartments (placed at different locations, symmetrically) and distribution of enzymes between compartments could qualitatively depend on which species is being localized.

We synthesize some basic insights from the study and then discuss the relevance of the results from three perspectives: methodologies for investigating spatial organization in metabolic networks, elucidating the role of spatial organization and engineering spatial organization.

### 4.5. Basic insights

By focusing on simple systems, described with mass-action kinetics, we have already uncovered different kinds of signatures associated with spatial localization: key structural signatures, unintuitive qualitative behaviour and alterations to the sensitivities. This already indicates that spatial localization introduces different dimensions to the kinetics, and its effect cannot simply be subsumed within an existing kinetic structure by an alteration of parameters. It also means that a qualitative mismatch between data obtained experimentally (for instance, by lysing cells and measuring spatial averaged output) and results expected from kinetic considerations alone may be owing to spatial localization rather than additional kinetic factors. This would suggest the need for focused spatially resolved studies. If spatial organization is a key contributor, then trying to account for the behaviour via kinetic model structures (even using inference or machine learning) can be fundamentally limiting. A very basic insight associated with systems with spatial organization is that spatial averaging before modelling and analysing the system is not the same as modelling the (spatial) systems, and averaging the results following that. This point recurs repeatedly. The localization of even a single species can introduce multiple trade-offs: kinetic trade-offs (absent in the kinetic model) and trade-offs associated with spatial factors. The presence of trade-offs points to ways of optimizing the system in light of this.

### 4.6. Methods for investigating spatial metabolic systems

Spatial organization is always present in metabolic systems. Even if spatial organization is not a focal point and if only non-spatial data are collected (e.g. spatial averaged or local concentrations), it can have a significant impact behind the scenes. Completely ignoring it runs the risk of making wrong attributions in non-spatial models, and this can result in incorrect conclusions and predictions, when the model is used extrapolatively. Although compartmental models can be useful, they have important limitations [51]. This is true of any compartmental or ODE model, including ones obtained from machine learning. A spatially explicit model accounting for size and location of compartments as well as transport (both within and in between compartments) is needed. This also means that a kinetic description (rather than a flux balance model) is ideally needed. Analogues of standard control coefficients for local or spatially averaged concentrations or fluxes can be calculated and used in conjunction with spatial control coefficients.

Our approach combines bottom-up and top-down analysis, providing complementary insights and helps to build a bridge from the simpler to the more complex systems. It can thus help bridge the gap between correlation and causation in specific systems, explicitly revealing a range of causational effects including unexpected ones. With an increasing interest in spatial organization and the variety of data driven and computational approaches and tools therein, there is still the need to achieve a transparent understanding of the key effects of spatial localization. Our approach creates a basic platform to provide this. The use of basic simplifications (consideration of motifs, simple patterns of localization in 1D) is a key to transparent understanding.

Investigating spatial localization in large metabolic systems can be computationally onerous given the large number of species and parameters. Our analysis suggests that for small-to-moderate metabolic networks (including synthetic networks), the analysis may be directly possible, given the small number of compartments: essentially the problem can be formulated as one of interacting networks located in different compartments, and a bottom-up analysis provides a basis for analysing this. A related approach is to focus on smaller sub-networks in a given network and build on that analysis. For larger systems, constraining system parameters and details based on experiments would be a useful way to start. On the spatial side, all our analysis uses 1D models, as this already contains the primary effects of localization of enzymes or substrates. Computational analysis in more realistic 2D geometries is facilitated by tools such as Virtual Cell, and the analysis above serves as a foundation for this.

### 4.7. Elucidating and understanding the effect of spatial organization in metabolic systems

Metabolic systems are large and unstructured. On the face of it, aiming to understand the role of spatial organization, going beyond predictions, appears formidably difficult. Further complicating the picture is that in contrast to signalling networks, metabolic networks are generally less prone (and have been less observed) to exhibiting a range of complex nonlinear dynamic behaviour. In signalling systems, the range of recurrent qualitative behaviour serves as a basic focal point for understanding different aspects of signalling (temporal, spatial [73] stochastic). Our analysis of basic motifs with and without feedback already provided a number of basic and key qualitative insights into the effect of localization. Then we examine a concrete exemplar system (glycolysis), which uses a model calibrated from experimental data in the literature [52]. We examined the effect of imposing spatial localization of different enzymes: PFK, ADH and TPI (a bifunctional enzyme); this provides insights and testable predictions into the effect of localization of these enzymes. We were also able to assess the extent of localization, the location of localization, the impact of localizing more than one enzyme and the impact of localizing an enzyme in more than one location (varying the distribution of enzyme between these locations). Assessing the effect of spatial organization here reveals a number of effects, which echo results seen in simpler motifs. It is interesting to contrast the new ingredients of the detailed model: a larger number of species, more complex description of the kinetics, the possibility of enzyme inhibition, an explicit description of energetics, bifunctional enzymes etc. In spite of all this, many basic effects continue to be observed. This suggests that as far as spatial localization goes, some of the basic effects are traceable to basic (even structural) considerations and may be observed in other biochemical networks too. There could be behaviour seen in the detailed network which does not echo behaviour in a motif (although we have not seen a striking example of that). On the other hand, there are behaviours seen in simpler motifs which may not be seen in a given detailed network for reasons ranging from additional complexity to ingredients, organization and parameter regime.

### 4.8. Engineering metabolic networks

A burgeoning technology for engineering localization and compartmentalization and the creation of a variety of compartmental structures provides many opportunities for spatially engineering metabolic networks. The creation of synthetic compartments in metabolic systems [74] was an early example of this. We consider two types of engineering problems: the first is a conventional engineering of metabolic networks, where spatial localization is present. Our analysis of metabolic control coefficients in this instance indicates that by ignoring spatial organization, incorrect conclusions may be made regarding key systems drivers, bottlenecks, optimal enzymes to manipulate. Thus, engineering approaches ignoring this could result in sub-optimal approaches (for instance, targeting an enzyme which is not the best choice). The second kind of engineering is spatial engineering of localization or compartmentalization of species. Given the significant strides in the creation of different compartments, networks of compartments etc., this is very feasible. In order to engineer localization to improve specific outputs (fluxes/concentrations), there are a number of things to be varied: the choice of species to be localized, the locations, sizes and number of compartments and how they are distributed between them. While a best choice can be arrived at in principle by considering a range of possibilities for each of these factors simultaneously, we approached the analysis sequentially mirroring a basic engineering approach. We thus consider the choice of species, followed by the (total) size of compartments followed by the number and location of compartments. Progressing in this way already provides transparent insights into the interplay between these factors, and provides practical guidance for engineering. We reveal how spatial localization can circumvent basic kinetic restrictions, pointing to its appeal. The analysis also reveals trade-offs associated with spatial localization which can occur in different ways. (i) The manipulation of a fixed factor can have a non-monotonic impact on an output measure of choice indicative of in-built competing effects. (ii) The manipulation of a factor can impact different output measures in different ways qualitatively, having a desirable impact on some and an undesirable effect on others. We find examples of each of the two classes of trade-offs, for cases where the parameter/factor can be either a kinetic factor or a spatial factor (e.g. compartment size). In each of these cases, we can establish that the trade-off arises as a consequence of spatial localization and would be absent in well-mixed systems. Thus, localization can also induce kinetic trade-offs. More generally, the presence of nested and interacting trade-offs in the introduction and manipulation of spatial localization is a key outcome of our analysis and strongly suggests the possibility of optimally engineering these various factors to maximize desired outcomes. The dynamic manipulation of localization is another aspect which can build on the study here.

### 4.9. Higher dimensions

Our analysis throughout has used 1D spatial models. Since describing spatial localization in 1D already involves explicit descriptions of localization and transport, many basic aspects of the interplay of kinetics and diffusion are already seen here. Some of the results such as the structural invariants directly generalize to higher dimensions (in a geometry with no-flux boundary conditions). This is because the essentially same analysis directly carries through there (incidentally, these results also generalize to cases where the diffusion coefficient may not be constant). This is shown in the appendices. In other cases, the 1D provides insights which are relevant in higher dimensions (in the simplest case where the geometry is quasi-1D to start with and basic perturbations thereof). Naturally, the consideration of higher dimensions also brings up different aspects: different spatial and spatio-temporal behaviour (in systems with feedback), the effect of compartment shape and location in the broader domain. Similarly, engineering considerations such as splitting or altering location of compartments are more complicated in higher dimensions owing to the need to engage with shape, size and location of those compartments in the ambient domain. It is conceivable that additional new aspects will emerge in those cases. However, it is also clear that to engage with those aspects systematically and in a structured fashion, the 1D case provides a platform and launchpad for exploring distinctive aspects which emerge in higher dimensions and different geometries.

All in all, the analysis of localization in metabolic systems, spanning top-down and bottom-up approaches, provides a number of insights relevant to understanding metabolic systems in cells and engineering them in natural and artificial cells and cell-free environments.

## Supplementary Material



## Data Availability

All results obtained are from models presented in detail in the text, and all the data are provided in the text or supplementary material. Additional code is included as supplementary material. Sample codes for different parts of the paper are presented in figures 1, 2, 5 and 7. Supplementary material is available online [75].

## Appendix A: Methodology

A significant part of the study focused on employing a bottom-up approach to investigate the effect of spatial organization, in particular spatial localization on metabolic networks. This started with the exploration of two-node and three-node motifs. Here, a variety of two-node motifs and three-node motifs are examined, accounting for different degrees of reversibility or irreversibility, and different degrees of outflow. The impact of localization of both enzymes and substrates was examined. This was built upon by examining the effect of feedback (specifically substrate–enzyme feedback) which gives rise to significant nonlinearity, and results in oscillations and bistability even in well-mixed systems. The analysis is expanded to account for metabolic control analysis, to assess the sensitivity of key output to both enzymes and spatial factors (compartment size, location). The impact of both localization whether implicit or explicit is evaluated carefully. This is then expanded to explore the consequences for spatial engineering of these systems. Finally, these bottom-up studies are complemented by a top-down study of an exemplar case (glycolysis). Details associated with the relevant methodology are presented below.

All the computational work was done on MATLAB. The resulting model equations are a coupled system of reaction–diffusion equations, with the localization of specific enzymes (or possibly certain substrates). The PDEs are discretized by employing a standard finite difference discretization of the PDEs in a 1D spatial domain. A spatial domain in 1D is suitable as a starting point for the range and type of analysis which we wish to perform, and the basic insights which we wish to draw. Many of the basic results also apply to 2D (in particular structural results carry through directly, and other insights have echoes in 2D). Higher dimensions can present additional aspects, and in fact our study provides a platform for exploring many 2D aspects in detail subsequently. Typically, a spatial grid of 200 points is used. This results in a set of ODEs which was solved using the ode15s solver with absolute tolerance and relative tolerance of $10^{-8}$ and $10^{-9}$, respectively. For all simulations, a no-flux boundary condition was applied at the boundaries. Two kinds of localization are considered: enzyme localization and substrate localization. We predominantly study enzyme localization. All the models used in the bottom-up studies involve mass-action descriptions of the kinetics. Thus, the enzyme amount is a parameter, and enzyme localization is implemented simply by restricting the location of the enzyme in space. In some instances, the enzyme concentration in the localized region is elevated in inverse proportion to the size of the localization, to ensure the total amount of enzyme is the same as the unlocalized case. When substrate localization is implemented, this includes the localization of enzymes which produce it. Here, the substrate is contained in a localized region. It is allowed to diffuse, and a no-flux boundary condition is applied at the boundary of the localized region (this thus includes the special case of a non-diffusible substrate uniformly present initially in this compartment, as a special case). The other species are allowed to freely diffuse across this localized region, including in particular across its boundaries. For simplicity, we treat the boundaries of this compartment as simply as possible, and do not explicitly incorporate the effect of any bounding membrane in affecting the flux. Those aspects have a modulatory effect, which can easily be understood and incorporated subsequently, if needed.

The model is written in dimensionless terms. Throughout the study, the total domain size was kept constant at a length of 10. However, other spatial factors, such as compartment size, compartment location, diffusivities and even the choice species to be localized, were examined. The sensitivities of the kinetic parameters and the enzymatic amounts were also evaluated for a range of values. Additionally, bifurcation analysis was performed on the kinetic ODE description for the cases with enzyme–substrate feedback to discover the expected behaviours which emerge from the feedback. This provides insights into key parameters and their ranges, which are useful for exploring the PDE models and the effects of localization subsequently. The computation of metabolic control coefficients (e.g. sensitivities of key outputs such as spatially averaged concentrations and fluxes to enzyme concentrations) was performed by using differences to approximate the relevant derivatives. Here, COPASI [76] was utilized as a tool for cross-validation, in the spatially homogeneous (non-localized) case: in COPASI, the model structure is provided along with parameters, and the model generation as well as differencing is done automatically. Thus, the cross-validation by COPASI serves to confirm that the model was correctly implemented in MATLAB and also that differencing was done to a sufficient accuracy to obtain the control coefficient. In the exemplar case, a calibrated model from the literature was used. Here, the model was downloaded and accessed using COPASI and was used for extensively cross-validating the MATLAB model which was created. This was done by considering the spatially homogeneous case (no localization) in which the model reduced to the kinetic model, which was cross-validated using COPASI.

### A.1. Biochemical building blocks

The biochemical building blocks we employ as the basis for our study are a combination of two-node and three-node motifs. Here, the nodes represent the species, and the edges represent the enzyme-mediated reactions. Motifs have been used as a point of departure for different kinds of analysis in systems and synthetic biology. In the current instance, it allows us to focus on key aspects of the interplay between spatial localization and the reaction network. The motifs we consider here all involve both inflows (typically one inflow, for simplicity) and one or two outflows.

#### A.1.1. Two-node systems

In the two-node system, the most general description follows that presented in figure S8 in the supplementary material. This shows two influxes and two effluxes (one of each, for each of the two nodes), along with two interconversion reactions between the two nodes, combined together to generate six different reactions that may take place in the system. Two different kinds of localization can be imposed on the system. The first is the localization of a substrate, which would of necessity require localizing all of the enzymes responsible for its generation, as shown in the right panel of figure S8 in the supplementary material. As mentioned above, when substrate localization is considered, any localized species is allowed to diffuse freely in the compartment (sub-domain) with no-flux boundary conditions imposed for this species at the edges of the compartment. Other substrates are allowed to diffuse freely across the full spatial domain. The second type of localization is the localization of enzymes which is shown in the left panel of figure S8 in the supplementary material. In this description, only a specific reaction is localized; hence, both substrate species can diffuse freely throughout the domain.

Note that in our models, the enzymes are assumed to be non-diffusible. Furthermore, for simplicity, all enzyme-mediated reactions are described using mass-action kinetics. Employing such a description allows us to isolate the broadest and most basic aspects of the impact of spatial localization. This implies that the enzyme total amounts can be treated as parameters, and localization of enzymes amounts to localization of the parameters.

For each of the two different localization scenarios, a number of cases were evaluated. These cases pertain to two aspects: kinetic and spatial. For example, for a given species localized (enzyme or substrate), every possible sub-network (which arises from eliminating one or more reactions) of the parent full two-node or three-node network was considered, as long as it was consistent with the particular localization scenario. Each of these sub-networks constitute simplifications of the original parent network and examining these together allows us to pinpoint the effect of localization and how it depends on each of the reactions. On the other hand, every possible substrate and enzymatic localization for each different reaction network/sub-network was evaluated. In doing this, if any system that had generated a similar description as another already examined, owing to symmetry, it was overlooked. For example, if a system with an inflow to A and an outflow from B was studied for a localization of the substrate A, then a system with an inflow in B and an outflow from A for a localization of B was discarded as it is the exact system with a different labelling. The following is the general model description used:

$$\begin{array}{rcl} \frac{\partial A}{\partial t} & = & k_{0a}E_{a}-k_{1f}E_{ab}A+k_{1b}E_{ba}B-k_{da}E_{ad}A+D_{A}\frac{\partial^{2}A}{\partial x^{2}},\\ \frac{\partial B}{\partial t} & = & k_{0b}E_{b}+k_{1f}E_{ab}A-k_{1b}E_{ba}B-k_{db}E_{bd}B+D_{B}\frac{\partial^{2}B}{\partial x^{2}}, \end{array}$$

where $k_{0a}$ and $E_{a}$ represent the rate constant and enzyme for the generation of a species A, and $k_{0b}$ and $E_{b}$ represent the rate constant and enzyme for the generation of a species B. $k_{1f}$ and $E_{ab}$ denote the rate constant and enzyme concentration associated with the A to B conversion, while $k_{1b}$ and $E_{ba}$ denote the rate constant and enzyme concentration associated with the B to A conversion. $D_{A}$ and $D_{B}$ denote the diffusion coefficients (assumed constant) associated with species A and B, respectively. *x* denotes the spatial coordinate.

Spatial localization/compartmentalization was implemented in these reaction–diffusion models. The localized species, be it the substrate or the enzyme, was then localized in a compartment whose size is 20% of the total spatial domain. The location of the compartment was centred in the middle of the domain. Subsequently, the location of the compartment was also varied. Furthermore, the size of the compartment was varied from 10% to 90% of the full domain (when the compartment was centred in the middle of the spatial domain). Additionally, for each different compartmental scenario, the sensitivity of a single parameter was evaluated.

In the instances when the size of the compartment was varied, two different scenarios relating to the enzymes were evaluated. In one scenario, the concentration of the enzyme was fixed, implying that the total amount of enzyme in the system varies with the size of the compartment. In the second scenario, the total amount of the enzyme is fixed, implying that the concentration of the enzyme varies in inverse proportion to the compartment size.

Building on these studies, a limited set of studies of two localizations was performed. On the one hand, the localization of two different enzymes was examined. On the other hand, localization (either of the same enzyme or different enzymes) was performed. Here, the effect of splitting a compartment was also evaluated, by examining two compartments half the size of the original compartment (placed symmetrically about the original compartment) and compared them with the single one. Additionally, we also explored different asymmetric distributions of a given enzyme between two compartments, for fixed total amounts of enzymes.

#### A.1.2. Three-node systems

Progressing from a two-node to a three-node description created additional permutations. As part of this, it was possible to isolate different structural categories: these include a linear reaction circuit, a branched reaction circuit and a cyclic reaction circuit. In each of these cases, different degrees of reversibility of the associated reactions between substrates can be examined. Unlike the two-node case, not every combination in every possible configuration was simulated, owing to the combinatorially larger number of possibilities. Instead, based on the analysis of two-node systems, we focused on a selection from all possible three-node systems, which seemed to be of importance and which were essentially different from one another. We also examined each of the three structural categories mentioned above, and examined a selection of cases in each of those categories. Given the central importance of degradation/outflow, we examined cases of both one outflow and two outflows. We focused on the case of one inflow only. The selection of scenarios we considered are shown in figure S9 in the supplementary material.

The following is the general model description for the three-node motif (completely reversible, allowing for inflow and outflow of all variables):

$$\begin{array}{rcl} \frac{\partial A}{\partial t} & = & k_{0a}E_{a}-k_{1f}E_{ab}A+k_{1b}E_{ba}B-k_{3b}E_{ac}A+k_{3f}E_{ca}C-k_{da}E_{da}A+D_{A}\frac{\partial^{2}A}{\partial x^{2}},\\ \frac{\partial B}{\partial t} & = & k_{0b}E_{b}+k_{1f}E_{ab}A-k_{1b}E_{ba}B-k_{2f}E_{bc}B+k_{2b}E_{cb}C-k_{db}E_{db}B+D_{B}\frac{\partial^{2}B}{\partial x^{2}},\\ \frac{\partial C}{\partial t} & = & k_{0C}E_{c}+k_{2f}E_{bc}B-k_{2b}E_{cb}C+k_{3b}E_{ac}A-k_{3f}E_{ca}C-k_{dc}E_{dc}C+D_{C}\frac{\partial^{2}C}{\partial x^{2}}. \end{array}$$

The terminology is similar to that of the two-node motif. Simply eliminating some terms (by setting associated rate constants to zero) would result in the model of the specific motif being assessed. All of these systems were analysed similar to the basal spatial case of the two-node system. Generally, the linear motif structure is similar to that of a two-node motif. The focus was more on the other motifs. Additionally, in cyclic motifs, we examined multiple species localization, as well as multiple compartments.

### A.2. Nonlinear systems

The study was expanded to evaluate nonlinear systems, the nonlinearity arising from the substrate enzyme feedback. These systems are capable of exhibiting non-trivial dynamical behaviour, even in well-mixed systems. The focus is on exploring the impact of spatial localization on these systems. Three systems were identified, two of which show oscillations and one that shows bistability. These behaviours were initially examined in ODEs and assessed using bifurcation analysis. The bifurcation analysis was conducted in MATCONT. All the three systems are two-node systems that are highly nonlinear. The models and the parameters for the bistable case and one of the oscillation cases (1) are adopted from [27]. The model for the oscillation case (2) was a refinement/augmentation of the model of the oscillations (case 1) so that autocatalysis was not the only route for A to B conversion. The parameters for oscillations in this case were identified using bifurcation analysis. The systems are summarized below: figure S10 in the supplementary material shows the reaction schematic. The mathematical models for each of the three systems are listed below.

Bistability case:

$$\begin{array}{rcl} \frac{\partial A}{\partial t} & = & k_{0a}E_{a}-\frac{k_{1f}E_{ab}A}{1+(k_{fb}A{)}^{4}}-k_{da}E_{da}A+D_{A}\frac{\partial^{2}A}{\partial x^{2}},\\ \frac{\partial B}{\partial t} & = & \frac{k_{1f}E_{ab}A}{1+(k_{fb}A{)}^{4}}-k_{db}E_{db}B+D_{B}\frac{\partial^{2}B}{\partial x^{2}}. \end{array}$$

Oscillations case 1:

$$\begin{array}{rcl} \frac{\partial A}{\partial t} & = & k_{0a}E_{a}-k_{1f}E_{ab}AB^{4}+D_{A}\frac{\partial^{2}A}{\partial x^{2}},\\ \frac{\partial B}{\partial t} & = & k_{1f}E_{ab}AB^{4}-k_{db}E_{db}B+D_{B}\frac{\partial^{2}B}{\partial x^{2}}. \end{array}$$

Oscillations case 2:

$$\begin{array}{rcl} \frac{\partial A}{\partial t} & = & k_{0a}E_{a}-k_{1f}E_{ab}A(1+k_{fb}B^{5})+D_{A}\frac{\partial^{2}A}{\partial x^{2}},\\ \frac{\partial B}{\partial t} & = & k_{1f}E_{ab}A(1+k_{fb}B^{5})-k_{db}E_{db}B+D_{B}\frac{\partial^{2}B}{\partial x^{2}}. \end{array}$$

All three systems were evaluated under various possible localization mechanisms. During this analysis, different compartment sizes were evaluated, ranging from 10% to 90% of the compartment size. Additionally, the effect of varying the diffusion coefficient of both species was evaluated, and careful consideration was taken to evaluate equal and contrasting diffusivities. Additionally, the parametric sensitivities were analysed for different compartment sizes.

#### A.2.1. Parameters used in motifs

All the above motifs involve a number of kinetic parameters. In considering spatial localization, other parameters such as diffusivities and compartmental sizes are required. In explaining the choice of parameters, we first reiterate the goal of the study which is to understand the interplay of spatial localization and metabolic networks. Our study was underpinned by the study of a number of two- and three-node motifs. These could be regarded as special cases of overarching two-node and three-node motifs, but we preferred to analyse these motifs separately. In a given motif, we chose nominal rate constants for interconversion reactions, which were equal to one another and studied the impact of spatial localization. These interconversion rate constants were kept constant across all motifs. Typically, the only kinetic rate constant which was varied was the outflow rate constant. This was the starting point for the analysis we perform.

This was repeated for other parameter sets. Out of such analysis emerged recurrent basic effects of spatial localization. These are presented in the text for a sample nominal parameter choice, but have been observed for both multiple parameters sets and multiple motifs. As an example, the biphasic behaviour in figure 2f was observed when multiple different kinetic parameters were perturbed significantly (see figure S2 in the supplementary material). In the context of feedback motif, feedback was a key parameter. It was varied using bifurcation analysis to assess what transitions it engendered. This was then directly used in the analysis. Importantly, it was possible in many instances to transparently understand the source of the behaviour. In terms of spatial parameters, again, in the above analysis, we used sample diffusivities of species which were comparable to one another. Again the basic results can be observed in multiple parameter sets, including variation of diffusivities. Basic insights regarding the effects of diffusvities are mentioned in §2.1. In the case of generation of Turing patterns, a sufficient contrast in diffusivities was needed, and this was explicitly probed. In terms of localization characteristics, the standard baseline employed in the paper is that of a compartment whose size was one-fifth the size of the full domain. Here again, the effect of the size of localization was probed (in motifs with feedback, metabolic control coefficients and spatial engineering). Finally some basic analysis regarding the effect of compartment location was also performed in §§2.3 and 2.4.

#### A.2.2. Parameters and the analysis performed

We summarize a few points about of parameters in relation to the different studies performed in different sections of the paper. The results on basic motifs show a combination of structural results (these are independent of parameters, and in fact continue to hold good even with significant model perturbations such as non-constant diffusivity). Alongside this, we show the existence of other behaviours which are not seen in the kinetic model and specifically arise from localization. Here, we note a few points. (i) We show that behaviour such as the biphasic response is observed even when other auxiliary parameters are perturbed significantly (supplementary material, figure S2). (ii) Behaviour seen in two-node motifs is also seen in three-node motifs where the parameters of the augmentation are not small: in other words, this represents a significant perturbation (not just parametric, but structural as well) and the behaviour can still be observed. Incidentally, echoes of different behaviour from motifs are also seen in the exemplar case. In the focus on feedback, there are a small number of parameters, which ultimately govern key behaviour: contrast of diffusivities allowing for Turing patterns (well studied in the literature) and the effect of feedback, which we directly probe. In the case of metabolic control coefficients, while control coefficients naturally depend on parameters, our goal is to demonstrate what can happen to this as a consequence of spatial perturbation. In our exploration underpinning this study, we encountered such behaviour in multiple parameter sets (as an example, a biphasic dependence of control coefficient on localization could be observed even with a twofold increase in a nominal parameter). In the case of spatial control coefficients, we have demonstrated the effects of different perturbations ourselves: (i) the consideration of four two-node motifs (which could be regarded as structural and parametric perturbations of a single motif) and (ii) the consideration of variation of size of compartment or even location. Our goal here was to focus on basic and qualitative effects, and we do so, on the basis of a comparison between these cases. The study of engineering localization is performed in the spirit of a proof of concept, where we start with a nominal set of parameters and consider the different typical steps which can be performed. Our results here are a combination of illustration of the existence of trade-offs, and some sample effects which can happen when compartment characteristics are altered. For the concrete exemplar case (see below), parameters are used directly from the literature, which in turn has been calibrated against experimental data.

### A.3. Metabolic control analysis

Metabolic control analysis has been a cornerstone in the analysis of metabolic systems using ODE models. Here, we examine two aspects of this when we deal with spatial systems with localization of enzymes. The first aspect is defining analogues of the standard metabolic control coefficients, which represent the sensitivity of specific concentrations or fluxes to varying enzyme concentrations. In our case, the enzymes are either localized in a sub-domain or present in the entire spatial domain. We evaluate specific observable outputs: the spatially averaged concentrations or fluxes, where the averaging may be done over a compartment or over the full spatial domain. Thus, we are assessing the sensitivity of key spatially averaged measures to changes to enzyme concentrations. When there is no localization present and the system is spatially homogeneous, these control coefficients reduce to the standard metabolic control coefficients (and we are able to cross-check our computations with that from COPASI). In each case, the flux and concentration control coefficients (for both the average over the full domain and the average of the compartment) were evaluated using equations (A 1) and (A 2), shown below:

(A 1)CEiJ=∂ln⁡J∂ln⁡Ei,(A 2)CEiS=∂ln⁡S∂ln⁡Ei,

where $C_{E_{i}}^{J}$ represents the flux control coefficient, which corresponds to the degree of control $E_{i}$ has on flux, *J*. Similarly, $C_{E_{i}}^{S}$ is the concentration control coefficient.

In these equations, the concentrations and fluxes correspond to the relevant spatially averaged concentrations and fluxes (either averaged over the full domain or the compartment). In addition to these kinetic control coefficients, three additional control coefficients were generated. These three are all spatial in nature. One is in relation to the diffusivity of the species, while the others depend on the compartment characteristics, size and location.

The first spatial control coefficient is in relation to the diffusivity. It was generated to evaluate the degree of control that the diffusivity has on the flux and the concentrations. These are shown in equations (A 3) and (A 4):

(A 3)CDiJ=∂ln⁡J∂ln⁡Di,(A 4)CDiS=∂ln⁡S∂ln⁡Di.

The other (explicitly spatial) control coefficients are to evaluate the sensitivity of the relevant spatially averaged output to the size of the compartment and the location. In the 1D PDE description, the size can be characterized by the length of the compartment. With regard to location, the location was defined by the spatial coordinate of the centre of the compartment below. These are shown in equations (A 5)–(A 8):

(A 5)CcgiJ=∂J∂cgilJ,(A 6)CcgiS=∂S∂cgilS,(A 7)CliJ=∂lnJ∂lnli,(A 8)CliS=∂lnS∂lnli,

where *cg* denotes centre and *l* stands for length (size) of the full domain.

One important difference should be noted here. While the control coefficient with respect to size of the compartment has the form similar to other control coefficients, that is not the case for the sensitivity with respect to the location. In fact in the latter case, the multiplying factor associated with the derivative involves the size of the full domain (or another suitable measure such as the size of the compartment). This is owing to multiple reasons. Firstly, a spatial coordinate may be positive or negative and the logarithm of such a value may not be defined (as a suitable real number). Secondly, a homogeneous spatial shift of the full system altering its spatial coordinates should not alter the control coefficients. Thus a ‘standard’ control coefficient definition cannot be used here.

We note in passing that while we have defined the size sensitivity in usual logarithmic terms, it is possible to define it in non-logarithmic terms (e.g. using the length of the full domain as a multiplier of the standard derivative, rather than the length of the compartment).

The MCA analysis was conducted on all of the possible one inflow one outflow and one input two outflows systems for the two-node motifs described in appendix A.1.1. Additionally, it was conducted on a one inflow one outflow cyclic three-node system, as well as on a variation of that system which included an additional backward reactions between A and B nodes. The same two networks were then evaluated in the instance of one inflow and two outflows. In the three-node cases, the outflow was either through C or B and C. All inflows in every motif were through A.

All the kinetic control coefficients and the diffusion control coefficients were analysed with different compartment sizes. The sizes looked at were 20%, 40%, 60% and 80% of the domain size. Additionally, two cases were evaluated under this. The first is keeping the total amount of enzymes fixed. The second is keeping the concentration of the enzymes fixed.

All the spatial control coefficients were analysed in two settings. Firstly, this was done by keeping the other spatial compartment characteristic (size/location) fixed at nominal basal values. Following this, the effect of varying the other factor on the sensitivity with respect to the factor being considered was evaluated. Thus, we evaluate the size control coefficient for different locations of the compartment and the location control coefficient for different sizes. As mentioned earlier when sizes are altered, two different scenarios were considered: fixed enzyme amount and fixed enzyme concentration.

### A.4. Exemplar case study

We complement our bottom-up study of motifs with a top-down study of a concrete system: yeast glycolysis. For this purpose, we employ a model created by Smallbone *et al.*, based on a characterization of the various enzymes involved. A schematic of the underlying network analysed by Smallbone *et al.* is presented in figure S11 in the supplementary material. This network was analysed and expanded upon in a subsequent study into a model, and we will use this model as a starting point for our analysis. It is worth pointing out that the model incorporates many additional ingredients: Michaelis–Menten kinetics, bifunctional enzymes, feedback and explicit description of the energetics, which are not present in the simpler motifs.

Smallbone *et al.* had built different iterations utilizing experimentally derived data to evaluate possible models. The final model, iteration 18, was selected as the basis for this study [52]. (i) The model is taken as is with all parameter values and expanded to a 1D PDE model, and can be found in the supplementary material. (ii) The mathematical model was coded in MATLAB, incorporating diffusive transport and enzyme localization. (iii) The original model is available in SBML as part of the paper [52], of the original model. It was utilized in COPASI to compare and cross-validate the spatial model we coded in MATLAB. In the PDE model created, in the absence of any localization, the system is described by the kinetics. This has been extensively cross-checked under a number of conditions and parameter values with the original model, and found to be in complete agreement. (iv) The complete MATLAB model is available as a code attached to this submission.

The exemplar case was evaluated for different enzymatic localizations, different compartmental characteristics, for multiple compartment and multiple enzyme localizations. This was done allowing for two different possibilities of fixing the concentration or the total amount of the enzyme. Furthermore, the effect of distributing a fixed amount of enzyme between two distinct compartments was evaluated for multi-compartment cases.

The system contained 23 variables, which required a diffusion coefficient for each one. This large number of parametric space becomes challenging to navigate. However, we assigned an equal diffusion coefficient of 10 $\mu {\text{m}}^{2}\;{\text{s}}^{-1}$ for all species, which is comparable to what is typically found for an intracellular protein [77]. Additionally, a few other cases were examined, either by evaluating the model at high or low diffusion regimes (for all species), changing the diffusivity of a specific number of species relating to a selected reaction or by specifying the molecules associated with energetics (AMP, ATP, NAD, etc.) at a higher diffusion coefficient than the rest to generate a contrasting effect. We were still able to isolate many broad trends associated with the localization of enzymes.

## Appendix B: Analysis

### B.1. Analytical results

In this section, we summarize analytical results which were referred to in the main text. This is organized in the following order. (i) Firstly, we present structural results associated with motifs involving one or two outflows. (ii) Then we present results from analysing the kinetics of specific motifs, to indicate that behaviour, which was observed with spatial localization, would not be observed in the system if spatial localization was absent. (iii) Finally, we present some basic insights associated with control coefficients.

Before we proceed, we make a comment about the notation in what follows. The results which follow from the analysis of the model are structural in nature. For simplicity, we will subsume all enzyme concentrations in associated kinetic parameters. We will also use a simpler more transparent parametric notation, for analytical purposes.

For a two-node system, the parameters are as follows: $k_{fa}$, influx rate constant; $k_{1f},k_{1r}$, rate constant for conversion from A to B and vice versa; $k_{da},k_{db}$, efflux rate constants for A and B; $D_{A},D_{B}$, diffusivities of A and B. Depending on the specific motif, some of these constants may be zero.

The equations for the two node motif are

$$\begin{array}{rcl} \frac{\partial A}{\partial t} & = & k_{fa}-k_{1f}A+k_{1r}B-k_{da}A+D_{A}\frac{\partial^{2}A}{\partial x^{2}},\\ \frac{\partial B}{\partial t} & = & k_{1f}A-k_{1r}B-k_{db}B+D_{B}\frac{\partial^{2}B}{\partial x^{2}}. \end{array}$$

In the three-node motif, the parameters are: $k_{fa}$, influx rate constant; $k_{da},k_{db},k_{dc}$, efflux rate constants for A, B and C; $D_{A},D_{B},D_{C}$, diffusivities of A, B and C. The interconversion rate constants use two subscripts, the first denoting the reactant and the second the product. Thus, $k_{ca}$ is the rate constant for conversion from C to A. The equations for the three node motif are given by

$$\begin{array}{rcl} \frac{\partial A}{\partial t} & = & k_{fa}-k_{ab}A-k_{ac}A+k_{ba}B+k_{ca}C-k_{da}A+D_{A}\frac{\partial^{2}A}{\partial x^{2}},\\ \frac{\partial B}{\partial t} & = & -k_{bc}B-k_{ba}B+k_{cb}C+k_{ab}A-k_{db}B+D_{B}\frac{\partial^{2}B}{\partial x^{2}},\\ \frac{\partial C}{\partial t} & = & -k_{ca}C-k_{cb}C+k_{ac}A+k_{bc}B--k_{dc}C+D_{C}\frac{\partial^{2}C}{\partial x^{2}}. \end{array}$$

#### B.1.1. Structural results for biochemical motifs with one and two outflows

We first consider a two-node motif with one inflow (to A) and one outflow (through B), a motif which was studied in §2.1. Without loss of generality, we assume that the interconversion between A and B is reversible. Both A and B can diffuse.

We allow for the possibility of localization of either of the interconversion reactions. Adding the equations for A and B results in the equation

$$\begin{array}{rcl} \frac{\partial (A+B)}{\partial t} & = & k_{fa}-k_{db}B=D_{A}\frac{\partial^{2}A}{\partial x^{2}}+D_{B}\frac{\partial^{2}B}{\partial x^{2}}. \end{array}$$

An immediate consequence of this is the following. At steady state, the left-hand side is zero. Averaging over space from (*x* = 0) to (*x* = *L*) (i.e. integrating from 0 to *L* and dividing by *L*) results in both diffusion terms being eliminated (owing to no-flux boundary conditions at the two boundaries). This results in a simple equation:

$$\begin{array}{rcl} \left\langle B\right\rangle & = & k_{fa}/k_{db}. \end{array}$$

Here, ⟨B⟩ denotes the spatial average of B. In other words, the spatial average of B is fixed irrespective of any kinetic parameters or localization associated with the interconversion.

Incidentally even if localization is applied to the input, the fact that the spatial average of B is independent of interconversion rate constants is seen (as discussed in §2.1)

This easily generalizes to the case where there may be outflow through both A and B. In that instance, adding the equations for A and B results in

$$\begin{array}{rcl} \frac{\partial (A+B)}{\partial t} & = & k_{fa}-k_{da}A-k_{db}B+D_{A}\frac{\partial^{2}A}{\partial x^{2}}+D_{B}\frac{\partial^{2}B}{\partial x^{2}}. \end{array}$$

Just as before, assuming steady state and spatially averaging both sides of the equation between 0 and *L* results in

$$\begin{array}{rcl} k_{da}\langle A\rangle +k_{db}\langle B\rangle & = & k_{fa}. \end{array}$$

In other words, in this instance, a linear combination of the spatial average of A and the spatial average of B is fixed, irrespective of interconversion parameters and localization of interconversion reactions.

A few comments can be made about these results. (i) Having more than one inflow does not affect this result, and simply results in an additive constant term in the right-hand side of the equations above. (ii) The results generalize to motifs with more than two nodes. In each case, by adding all the equations, all interconversion reaction terms cancel out leaving only inflow, outflow and diffusion terms. The diffusion terms are eliminated by averaging resulting in equations analogous to those obtained. (iii) If more outflow variables are involved, that is simply reflected in the equation which emerges after averaging: a linear combination of the spatial average of all outflow species concentrations is fixed at a level which is independent of interconversion kinetic parameters and localization therein.

We now examine a case of the two-node motif with one outflow, where the outflow reaction is localized. Again, adding the equations for A and B results in interconversion terms cancelling out and this results in

$$\begin{array}{rcl} \frac{\partial (A+B)}{\partial t} & = & k_{fa}-k_{db}(x)B+D_{A}\frac{\partial^{2}A}{\partial x^{2}}+D_{B}\frac{\partial^{2}B}{\partial x^{2}}. \end{array}$$

The spatial dependence of the outflow rate constant is explicitly incorporated. For the case under consideration, $k_{db}$ is constant but localized in a particular sub-domain (i.e. it has a value zero outside the sub-domain). Now just as before, we assume steady state and spatially average the equation between 0 and *L*. Again the diffusion terms cancel out. However, the averaging of the outflow term simply results in $k_{db}\langle B\rangle_{c}L(c)/L$, where $\langle B\rangle_{C}$ denotes the spatial averaging across the compartment, and $L_{c}$ denotes the length of the compartment. This then results in the equation

$$\begin{array}{rcl} \langle B\rangle_{c} & = & k_{fa}L/k_{db}L_{c}. \end{array}$$

This indicates that in this instance, it is the compartmental average of the outflow variable that is fixed independent of interconversion reaction parameters.

#### B.1.2. Other two-node and three-node systems

In the text, we examined two different feedback scenarios for two-node systems with variables A and B. We considered the case of irreversible interconversion between A and B, where in one instance, the rate (and the rate constant) can be affected by A, whereas in another instance, it can be affected by B. The former scenario results in relatively simple steady-state behaviour, and this is owing to the fact that the equation for A is decoupled from B, and the behaviour of such scalar PDEs is generally well understood (and simpler than when there are multiple coupled variables). In the second instance, we have computationally observed different spatial behaviours such as Turing patterns.

We now comment on the structural insights above, in the context of feedback. We focus on a single outflow variable, though, just as noted above, this generalizes to multiple outflow variables. The first point to note is that with a single outflow variable, at steady state, the spatial average of the outflow variable B is independent of the interconversion (wherein lies the feedback) and any localization. In particular, this means that for any steady-state Turing pattern, the same condition holds good. A similar insight holds good for two outflow variables, where the condition obtained above continues to hold good.

We now extend this analysis to results which may have both spatial and temporal variations, for example, spatial patterns which may have periodic time variations. We now repeat the steps above. Firstly, by adding the equations for A and B, we have

$$\begin{array}{rcl} \frac{\partial (A+B)}{\partial t} & = & k_{fa}-k_{db}B+D_{A}\frac{\partial^{2}A}{\partial x^{2}}+D_{B}\frac{\partial^{2}B}{\partial x^{2}}. \end{array}$$

Now, we spatial average both sides of this equation, noting that the system is not at steady state. This results in the following equation:

$$\begin{array}{rcl} \frac{\partial (\langle A\rangle +\langle B\rangle }{\partial t} & = & k_{fa}-k_{db}\langle B\rangle . \end{array}$$

In the left-hand side above, we have interchanged the spatial average and the time derivative. Now suppose we have a pattern which varies periodically in time (for every location) with a time period *T*. Time averaging both sides of the equation (integrating from *t* to *t* + *T* and dividing the result by *T*) results in the left-hand side being zero (for a time-periodic pattern, noting that the integral of the derivative is the difference in variable evaluated at the beginning and end of the cycle). This results in a simple equation:

$$\begin{array}{rcl} \langle B\rangle_{T} & = & k_{fa}/k_{db}. \end{array}$$

In the above equation, $\langle B\rangle_{T}$ denotes the time average of the spatial average and is defined as

(B 1) $$\begin{array}{rcl} \langle B\rangle_{T} & = & (1/T)\int_{t=t}^{t=t+T}\left[1/L\int_{x=0}^{x=L}B(x,t)\;dx\right]\;dt. \end{array}$$

In other words, the temporal average of the spatial average of the outflow variable is fixed (this also represents the spatial average of the temporal average). In a similar way, it is possible to examine systems with more than one outflow variables, and analogous to the derivations above, we find that a linear combination of temporal averages of the spatial averages of the outflow variables is fixed independent of interconversion parameters, and localization therein. Again this result generalizes to systems with more inflows, nodes and outflow variables in a simple way.

There is a simple corollary of the results above. If the system exhibits time-periodic behaviour which is spatially homogeneous, then the above result continues to hold good. The one simplification is that the spatial average of the variable is simply the variable value itself. It then follows, in the case of the one-outflow case that

$$\begin{array}{rcl} (1/T)\int_{t=t}^{t+T}B(t)\;dt & = & k_{fa}/k_{db}. \end{array}$$

In other words, the time average of the outflow variable is fixed, depending only on the inflow and outflow parameters.

A general comment related to all the above results is worth pointing out. All the models above involve linear outflow rates. If the outflow depends nonlinearly on the outflow variables (e.g. via Michaelis–Menten dependence), then the above results will have to be modified. In those cases, the above results apply not for the average of the variables but for the averages of the associated (outflow) fluxes.

We now turn to a discussion of two results shown in §2.1. The first result pertains to an unintuitive observation: in a two-node reversible motif with one inflow (to A) and one outflow (B). The enzyme involved in the A to B reaction is localized. When changing the rate constant of the B to A reaction, the compartment average of B was found to increase. Here, we establish the point that this arises from the localization, by showing that this cannot arise in a well-mixed system. Indeed, by following the derivation above for the two-node case with one inflow and one outflow, we find that by adding the equations, we have

$$\begin{array}{rcl} \frac{\partial (A+B)}{\partial t} & = & k_{fa}-k_{db}B=0, \end{array}$$

from which it follows that $B=k_{fa}/k_{db}$ and is independent of the interconversion reaction rate constants. Thus, both the dependence on rate constants and the unintuitive behaviour arise from the spatial localization. Note that with spatial localization, the spatial average of B is fixed at $k_{fa}/k_{db}$, but this means that the average inside the compartment can be either below or above this average, the spatial average outside the compartment will behave in exactly the opposite way. We have shown how the average inside the compartment can actually increase, and shown how this can occur in bigger motifs as well.

Another unintuitive behaviour in the same motif is observed by localizing the B to A conversion, and varying the rate constant of the A to B reaction. Here, it is found that the average A concentration in the compartment can actually increase. Such behaviour cannot occur in well-mixed systems. Indeed, as observed above, in a well-mixed scenario, B reaches a steady state independent of the interconversion rate constants. Furthermore, the steady state of A then becomes

$$\begin{array}{r} A=k_{fa}(1+k_{1r}/k_{db})/k_{1f}. \end{array}$$

Increasing the A to B conversion rate constant ($k_{1f}$) only decreased the concentration of A at steady state.

The second result involves a three-node completely reversible motif with one inflow node (A) and one outflow node (C). The A to B conversion reaction is localized, and it is found that varying the A to C reaction rate constant elicits a biphasic effect on the compartmental average of C. Again, we establish that this arises from the localization by showing that for identical kinetics, the well-mixed system cannot give rise to such behaviour. In fact by adding all the equations, we have just as above

$$\begin{array}{rcl} \frac{\partial (A+B+C)}{\partial t} & = & k_{fa}-k_{dc}C=0, \end{array}$$

from which it follows that at steady state $C=k_{fa}/k_{dc}$. In other words, any dependence, including the biphasic dependence of any measure of C (in this instance the compartmental average) arises as a consequence of localization, and is not simply inherited from the kinetics.

Again, in the very same motif, a biphasic effect is seen, even if there are two outflow nodes (B and C). The fact that this cannot occur in a well-mixed system is demonstrated as follows.

First by adding all the equations, at steady state $k_{fa}=k_{db}B+k_{dc}C$, as also noted earlier. Now suppose there is a biphasic dependence of C on the A to C reaction rate constant $k_{ac}$. This means $dC/dk_{ac}=0$ at the biphasic extremum. Now from the equation above it also follows, by differentiating with respect to $k_{ac}$ that $dB/dk_{ac}=0$ at the biphasic extremum. Now by considering the steady state of B and A, we have

$$\begin{array}{rcl} k_{ab}A+k_{cb}C & = & (k_{ba}+k_{bc}+k_{db})B,\\ k_{fa}+k_{ba}B+k_{ca}C & = & (k_{ac}+k_{ab})A. \end{array}$$

By differentiating the first of the equations with respect to $k_{ac}$, we find that $dA/dk_{ac}=0$ at the biphasic extremum. If we differentiate the second of the two equations at the biphasic peak, we find using the results above that A=0, which is impossible (every other derivative has been shown above to be zero, and the only surviving term is what is obtained by differentiating kac with respect to itself). A=0 is not possible for any feasible state since it has positive production rates (including in particular through the inflow) and removal/conversion rates away from it linear in A.

Thus, the assumption of a biphasic dependence of C on $k_{ac}$ in well-mixed systems leads to a contradiction. In other words, this specific biphasic response cannot occur in well-mixed systems.

#### B.1.3. Comments on control coefficients

Our results in §§2.3 and 2.4 employ different control coefficients, some of which are analogues of standard kinetic control coefficients, and others are spatial control coefficients. We comment on each of these.

#### B.1.4. Kinetic control coefficients

Our kinetic control coefficients focus on key measures of the output: spatial averaged concentrations or fluxes, averaged either over the full spatial domain, or over a compartment. As mentioned above, there are different instances (e.g. single outflow cases) where the average concentration or flux (averaged over the full domain) is independent of interconversion enzyme levels/rate constants. In this instance, the control coefficients associated with enzymes involved in interconversion (for this measure of output) will be zero, even though interconversion enzymes do affect the local concentration of the species under consideration. In a similar way, the control coefficient of this species with respect to the inflow rate constant (or associated enzyme) will take the value 1. When there are two outflows (in a two-node or three-node motif, for instance), the control coefficients of the two outflow species with respect to any enzyme involved in interconversion reactions between species will necessarily have opposite signs. Another point to emphasize is that control coefficients (with respect to the same enzyme) of different output measures of the same species (e.g. domain average and compartment average) can be very different and even have different signs: computational results shown in the text indicate how these different measures can vary in quantitatively and qualitatively different ways.

#### B.1.5. Spatial control coefficients

We comment on the spatial control coefficients. For the control coefficient with respect to compartment length, this is defined in an analogous way to other control coefficients. We note in passing that there are other ways of defining this control coefficient, for example, $S/L_{tot}dS/dL$. Here, *L* is the size of the compartment under consideration, and $L_{tot}$ is the total domain size.

In our analysis of simple motifs, we show why certain compartment length control coefficients take the value −1. In particular, we consider the case in table 1 (in figure 6), for a two-node motif (with irreversible interconversion between nodes) with one outflow, in the situation where the outflow enzyme is localized. Here, a direct analysis along the lines of what was described above indicates that $k_{fa}L_{tot}=k_{db}\langle B\rangle_{c}L$, where $\langle B\rangle_{c}$ denotes the compartment average of B, *L* is the compartment size and $L_{tot}$ is the total domain size. Differentiating with respect to *L* results in $0=L\partial \langle B\rangle_{c}\partial L+\langle B\rangle_{c}$ which when reorganized gives $L/\langle B\rangle_{c}\partial \langle B\rangle_{c}\partial L=-1$. A similar conclusion can be made for the coefficients associated with the compartment average of the flux. Incidentally, in this scenario, the various spatial control coefficients for species A are zero because they are unaffected by the length of the compartment. In particular, by writing the steady-state equations for A, we see that they are completely independent of the degrading enzyme of B, as well as the size of the compartment in which it is localized. This aspect changes when A and B reversibly interconvert (see tables of control coefficients in supplementary material, figure S6).

With regard to sensitivity (of a target S) to compartment location, as mentioned elsewhere, we define this control coefficient as ($L_{tot}/S)dS/dx_{c}$, where $x_{c}$ is the location of the centre of the compartment. Note that we do not have an $x_{c}$ outside the derivative, as we wish the control coefficient to be independent of the coordinate system: translational shifts in the coordinate system should leave the control coefficient unchanged.

Finally, we comment on how these coefficients may be used when more than one factor is varied at the same time. Two examples of such variations are (i) variation of the compartment sizes of two compartments simultaneously and (ii) variation of size keeping total amount of enzymes fixed, implying that the enzyme concentration is also varied. We consider these in turn, and in each case, the starting point is the same.

In the first case, suppose two compartment sizes are varied simultaneously. Let us assume that the sizes are $L_{1}$ and $L_{2}$. Using the definition of the differential, we have $dS=\partial S/\partial L_{1}dL_{1}+\partial S/\partial L_{2}d_{2}$. Suppose $L_{1}=L_{2}=L$ and the size is varied equally. Then $dS/dL=\partial S/\partial L_{1}+\partial S/\partial L_{2}$. Multiplying by L/S, we have $L/SdS/dL=L/SdS/dL_{1}+L/SdS/dL_{2}$. In other words, the total control coefficient is the sum of individual control coefficients, evaluated at the basal compartment size.

In a similar way, we can evaluate the effect of varying the length keeping the total enzyme amount fixed. dS=(∂S/∂L)dL+(∂S/∂E)dE. Now since $E.L=E_{tot}$, differentiating with respect to *L* yields dS/dL=∂S/∂L+∂S/∂E(−E/L). Multiplying by *L*/*S*, we get L/SdS/dL=(L/S)(∂S/∂L)−E/S∂S/∂E.

The last two equations provide multiple insights. To start with, the key difference between dS/dL and ∂S/∂L is the last term. We see how these two terms could have different signs as seen in the tables in figure 6. We can use this information to also show how this implies an intermediate extremum as the size of the compartment is varied. We have already observed numerically that there are cases where the signs of these derivatives are different (which correspond to different signs of the size control coefficient when the enzyme amount is fixed and when the enzyme concentration is fixed) for some intermediate localization. We will analyse this situation further.

Now suppose the signs of these derivatives are the same for no localization (i.e. length of compartment equals the length of the domain), as has been observed numerically. Then as a consequence of changing the degree of compartmentalization (from 100% downwards), one of the derivatives has, of necessity, got to change sign (since the product of the two derivatives changes sign). Thus, from continuity arguments, one of the derivatives has to equal zero, for some intermediate compartment size. Incidentally, we have observed this when the total enzyme amount is fixed.

Now suppose both these derivatives have opposite signs for the case of no localization (i.e. 100% localization). This case can be argued as follows. Suppose under basal conditions, the enzyme concentration is $E_{0}$ and the length is $L_{0}$.

The relationship between the derivatives using the chain rule is $dS/dL=\partial S/\partial L-(E_{0}/L_{0})\partial S/\partial E$.

Note that all derivatives are evaluated to start with, at $E_{0},L_{0}$ and ∂S/∂E is determined completely by the kinetics. From above, we also see that (for fixed $E_{0}$) if make $L_{0}$ large enough (by increasing the size of the domain), the two derivatives have the same sign. We examine the effect of increasing the domain length starting from the basal domain length.

We first note that for a fixed domain length, the sign of any derivative with respect to compartment size is also the same as the sign of the derivative with respect to compartment fraction. We will use derivatives with respect to compartment fraction below.

First consider the basal case. By continuity arguments, we find a neighbourhood in the compartmentalization fraction of the 100% compartmentalization fraction, where the sign of each derivative is the same as that of 100% compartmentalization in the initial instance. Likewise in the final instance, we find another neighbourhood of the 100% compartmentalization fraction where the sign of each derivative is the same as that of 100% compartmentalization. By focusing on the intersection of these neighbourhoods and picking an arbitrary compartmentalization fraction, we find that one of the derivatives with respect to compartment fraction changes sign, as the domain length is changed, and so is consequently zero at some intermediate length. Thus at that length, one of the derivatives with respect to compartmental fraction is zero, and the derivative with respect to the size of compartment is also zero (since the length is fixed).

#### B.1.6. Structural invariants in higher dimensions

We showed different structural invariants: for instance, in a two-node (or higher) motif with only one outflow, the spatial average of the outflow variable is fixed, and depends only on the inflow and outflow rate constants. We were also able to show that for two outflows, a linear combination of spatial averages of the two outflow variables (each weighted by their respective outflow rate constants) was fixed, and equal to the inflow rate constant. We also showed that when the outflow in a single outflow motif was localized, then the compartment average of outflow variable was fixed (and depended again only on the inflow and efflux rate constants).

All these results were obtained in 1D by simply adding all the variables. All interconversions cancelled out leaving inflow, outflow and diffusion. Averaging over the spatial domain readily provided the result since the spatial average of diffusion terms cancelled out, owing to the no-flux boundary conditions.

The same approach carries through in higher dimensions, for the basic reason that the diffusion terms again cancel out when averaged over the spatial domain. As an example, in a two-node motif, adding the equations for A and B results in

$$\begin{array}{rcl} \frac{\partial (A+B)}{\partial t} & = & k_{fa}-k_{db}B+\nabla .(D_{A}\nabla A)+\nabla .(D_{B}\nabla B). \end{array}$$

Now at steady state, the left-hand side is zero, and averaging over the spatial domain results in $k_{fa}=k_{db}\langle B\rangle$ just as before. This is because the integral of the diffusion terms over the domain continues to be zero. This in turn is owing to the application of the Gauss divergence theorem so that ${∭}_{V}\nabla .(D_{A}\nabla A)\;dV=\iint_{S}D_{A}\nabla A.n\;dS$ (*n* represents the normal vector). This second term is zero since it is the surface integral of the normal flux, and with no-flux boundary conditions is zero. The same argument applies for the integral of the diffusion term for B. Thus, we see that the structural results obtained earlier all carry through in higher dimensions. In a similar way if the outflow of B is localized, again the same kind of result is obtained, since we again have steady state and the spatial integral of the diffusion terms is zero just as before. Structural results involving two outflows are again obtained in analogous fashion.

When we consider motifs with feedback, we showed that at steady state, the spatial average of the outflow variable is fixed, and when spatio-temporal oscillations are observed, the time average of the spatial average is fixed (in both instances only depending on the inflow rate constant and the outflow rate constant). The way this is obtained is again by adding the equations, and then averaging spatially (and subsequently temporally over a periodic cycle in the case of spatio-temporal oscillations). Again the spatial averaging leads to the diffusion terms dropping out for the same reason, as demonstrated above.

*Note.* Note that in the analysis above, the spatial average of the diffusion terms being zero continues to hold good even if the diffusivity is not constant. This is seen directly above: in the use of Gauss divergence theorem, we did not need to make the assumption of constant diffusivity. Consequently, the results above hold good even in this instance (in both one and higher dimensions).

### B.2. Parameters

#### B.2.1. Biochemical building blocks

Figure 2

(a) $k_{0a}=2,E_{ab}=1,k_{1b}=0,E_{ba}=0,k_{da}=0,E_{da}=0,k_{0b}=0,E_{b}=0,k_{db}=5,E_{db}=1,D_{A}=9,D_{B}=5,k_{1f}=1,2,5,7,10,20,50,100,IC:A_{0}=0,B_{0}=0,E_{a}=1$ at 20% of the middle of the domain.

(b) $k_{0a}=2,E_{a}=1,E_{ab}=1,k_{1b}=10,E_{ba}=1,k_{da}=0,E_{da}=0,k_{0b}=0,E_{b}=0,k_{db}=5,D_{A}=9,D_{B}=5,IC:A_{0}=0,B_{0}=0,k_{1f}=1,2,5,7,$$10,20,50,100,E_{db}=1$ at 20% of the middle of the domain.

(c) $k_{0a}=2,E_{a}=1,k_{1f}=10,E_{ba}=1,k_{da}=0,E_{da}=0,k_{0b}=0,E_{b}=0,k_{db}=5,E_{db}=1,D_{A}=9,D_{B}=5,k_{1b}=1,2,5,7,10,20,50,100,IC:A_{0}=0,B_{0}=0,E_{ab}=1$ at 20% of the middle of the domain.

(d) $k_{0a}=2,E_{a}=1,k_{1f}=10,k_{1b}=10,E_{ba}=1,k_{da}=7,E_{da}=1,k_{0b}=0,E_{b}=0,k_{db}=5,E_{db}=1,(D_{A}=9,D_{B}=1,2,5,7,10,20,50,100)\&$$(D_{B}=5,D_{A}=1,2,5,7,10,20,50,100),IC:A_{0}=0,B_{0}=0,E_{ab}=1$ at 20% of the middle of the domain.

(e) $k_{0a}=2,k_{1f}=10,k_{1b}=10,k_{da}=7,k_{0b}=0,E_{b}=0,k_{db}=5,E_{db}=1,(D_{A}=9,D_{B}=1,2,5,7,10,20,50,100)and(D_{B}=5,D_{A}=1,2,5,7,$$10,20,50,100),IC:A_{0}=0,B_{0}=0,(E_{a}=1,E_{ab}=1,E_{ba}=1,E_{da}=1)$ are at 20% of the middle of the domain.

(f) $k_{0a}=2,E_{a}=1,k_{1f}=10,k_{1b}=10,E_{ba}=1,k_{da}=0,E_{da}=0,k_{0b}=0,E_{b}=0,k_{db}=0,E_{db}=0,k_{0C}=0,E_{c}=0,k_{2f}=10,E_{bc}=1,k_{2b}=10,E_{cb}=1,k_{3f}=10,$$,E_{ac}=1,E_{ca}=1,k_{dc}=5,E_{dc}=1,D_{A}=9,D_{B}=5,D_{C}=3,k_{3b}=1,2,5,7,10,20,50,100,IC:A_{0}=0,B_{0}=0.C_{0}=0.E_{ab}=1$ at 20% of the middle of the domain.

#### B.2.2. Nonlinear systems

Figure 3

(a) and (b) $k_{0a}=1.5,E_{a}=1,E_{ab}=1,k_{1f}=0.8,E_{ab}=1,k_{db}=1.5,E_{db}=1,k_{fb}=1,D_{A}=D_{B}=1.IC:A_{0}=0,B_{0}=0.$

All subsequent cases use the same parameters and enzymes unless stated otherwise.

(c) and (d) $D_{A}=100,D_{B}=1$.

(e) and (f) $D_{A}=100,D_{B}=1,k_{fb}=10$.

(g) and (h) $D_{A}=D_{B}=100,E_{ab}=1$ at 60% of the middle of the domain.

(i) and (j) $D_{A}=D_{B}=1,E_{ab}=1$ at 60% of the middle of the domain.

Figure 4

Same model as before, using the same parameters except when stated otherwise.

(a–d) $D_{A}=100,D_{B}=1,k_{fb}=10,E_{ab}=1$ at 80% of the middle of the domain.

(e–g) $D_{A}=5,E_{ab}=1$ at 60% of the middle of the domain.

(h) $k_{0a}=0.5,E_{a}=1,E_{ab}=1,k_{1f}=0.4,E_{ab}=1,k_{da}=0.05,k_{db}=0.5,E_{db}=1,k_{fb}=1/3,D_{A}=1,D_{B}=1,2,5,7,10,20,50\;100,IC:A_{0}=0,B_{0}=0.$

(i) same as (h) but different: $IC:A_{0}=10,B_{0}=0$.

#### B.2.3. Metabolic control coefficients

Figure 5

(a–d) $k_{0a}=2,E_{a}=1,k_{1f}=10,k_{1b}=0,E_{ba}=0,k_{da}=7,E_{da}=1,k_{0b}=0,E_{b}=0,k_{db}=5,E_{db}=1,D_{A}=9,D_{B}=5,IC:A_{0}=0,B_{0}=0.E_{ab}$=1 at 20%, 40%, 60% and 80% of the middle of the domain and lastly without localization.

(e–f) $k_{0a}=2,E_{a}=1,E_{ab}=1,k_{1f}=10,k_{1b}=0,E_{ba}=0,k_{da}=0,E_{da}=0,k_{0b}=0,E_{b}=0,k_{db}=0,E_{db}=0,k_{0C}=0,E_{c}=0,k_{2f}=10,E_{bc}$=1,$k_{2b}=0,$$E_{cb}=0,k_{3b}=0,E_{ac}=0,E_{ca}=1,k_{3f}=10,k_{dc}=5,D_{A}=9,D_{B}=5,D_{C}=3,IC:A_{0}=0,B_{0}=0.C_{0}=0.E_{dc}=1$ at 20%, 40%, 60% and 80% of the middle of the domain and lastly without localization.

Figure 6

Tables focus on two two-node motifs studied with same nominal parameters as in figure 5a–d: table 2 and with certain arrows removed (rate constant zero) for table 1. Here, the efflux is localized.

#### B.2.4. Engineering perspective

Figure S7

(a) $k_{0a}=2,E_{a}=1,k_{1f}=10,E_{ba}=0,k_{da}=0,E_{da}=0,k_{0b}=0,E_{b}=0,k_{db}=5,E_{db}=1,D_{A}=9,D_{B}=5,IC:A_{0}=0,B_{0}=0.$

For this case, four different enzymatic conditions were applied:

1. $E_{ab}=1$ at 20% of the middle of the domain.
2. $E_{ab}=0.5$ in two compartments each 20% of the full domain: compartment 1 occupies 21–40% of the domain, while compartment 2 occupies 61–80% of the domain.
3. $E_{ab}=1$ in two compartments each 10% of the full domain: compartment 1 occupies 31–40% of the domain, while compartment 2 occupies 61–70% of the domain.
4. $E_{ab}=0.75$ in compartment 1; on the other hand, $E_{ab}=0.25$ in compartment 2. The two compartments are each 20% of the full domain: compartment 1 occupies 21–40% of the domain, while compartment 2 occupies 61–80% of the domain.

(b) $k_{0a}=2,E_{a}=1,k_{1f}=10,20,50,100,E_{ba}=0,k_{1b}=0,k_{da}=0,E_{da}=0,k_{0b}=0,E_{b}=0,k_{db}=5,D_{A}=9,D_{B}=5,IC:A_{0}=0,B_{0}=0.$$E_{ab}$$=E_{db}$=1 both at 20% of the middle of the domain.

(c) $k_{0a}=2,E_{a}=1,k_{1f}=10,E_{ba}=1,k_{da}=0,E_{da}=0,k_{0b}=0,E_{b}=0,k_{db}=5,D_{A}=9,D_{B}=5,k_{1b}=1,2,5,7,10,20,50,100,IC:A_{0}=0,$$B_{0}$=0.

This case has two enzymatic localization at different locations both are 20% of the size of the full domain.

1. $E_{ab}=1$ at compartment 1 occupying 21–40% of the domain.
2. $E_{db}=1$ at compartment 1 occupying 61–80% of the domain.

Tables S3– S9 in the supplementary material involve three motifs (with two outflows) which are studied previously; nominal motif parameters: A two-node reversible motif studied in figure 5a–d, a three-node irreversible motif studied in figure 5e,f, but with an efflux for B added (same rate constant as efflux of C), and a three-node reversible motif studied: parameters same as the irreversible three-node motif, but with forward and backward interconversion rate constants equal.
