# Supplementary material for: Elucidating the effect of spatial localization on metabolic systems
Source: J R Soc Interface. 2026 Oct 28;23(243):20251328. doi: 10.1098/rsif.2025.1328 (PMC13714402; doi:10.1098/rsif.2025.1328)
Supplement: Supplementary file 1 [file rsif.2025.1328.zip › rsif.2025.1328/rsif20251328_si_001.pdf]

Supplementary Material

Basic Network Motifs

Here, we evaluate a more general case of the 3-node motif, in which all reactions are present in the system. Imposing different localization schemes highlights the recurrence of most of the findings discussed under the results of the basic motifs in the main text. Furthermore, the spatial behaviour is presented to showcase the spatially heterogeneous steady state is present, to spatial organization. Figure S1, illustrates all of these findings with the different localizations imposed. Figure S2 examines the effect of varying parameters on the presence of biphasic behaviour studied in Fig. 2(f) of the main text.

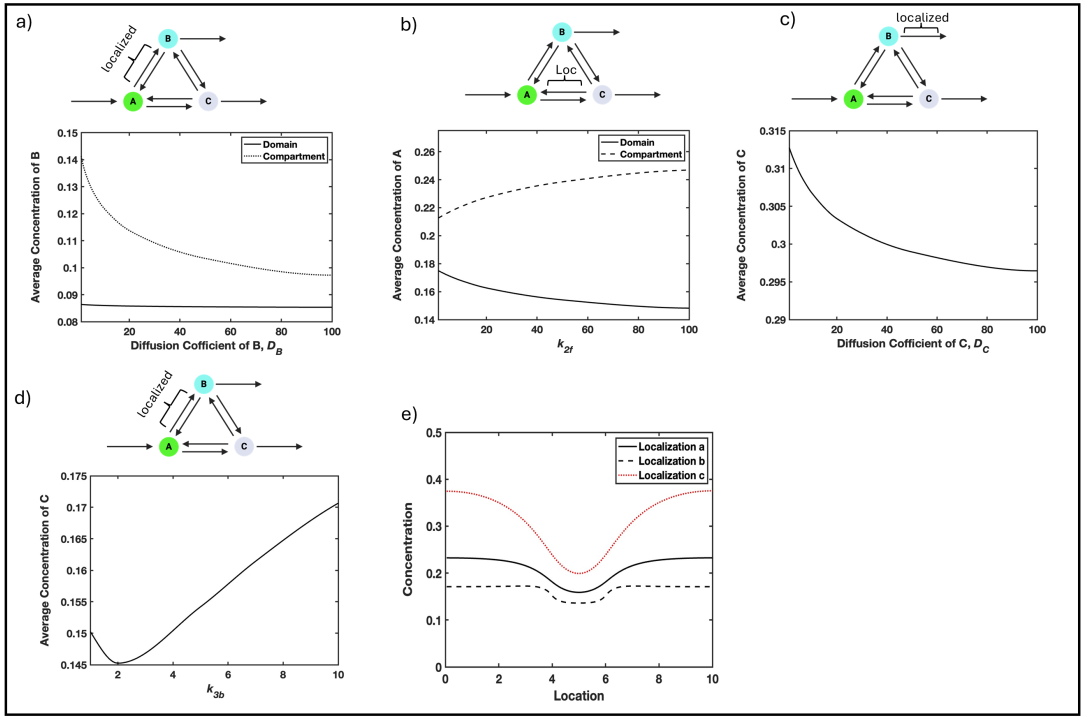

Figure S1: Behaviour of a basic 3-node motif with localization of enzymes In all cases, the relevant localization being investigated is depicted alongside the relevant plot (Created with Biorender). a) The average concentration of B over the full domain and over the compartment is plotted for different diffusivities of B when localizing the enzyme of the A to B reaction. b)A similar case to the previous, but the localized species is the enzyme of the C to A reaction. The average concentration of A over the full domain and over the compartment is plotted different values of the kinetic parameter for the B to C reaction. c) The imposed localization for this case is on the enzyme mediating the efflux of B. The average concentration of C over the domain is plotted for different values of the diffusion coefficient of C. d) Similar to a) but the compartmental average concentration of C is plotted for different values of the rate constant of the C to A reaction. e) evaluates the steady state spatial profile of the basal case for the three models depicted in a),b) & c)

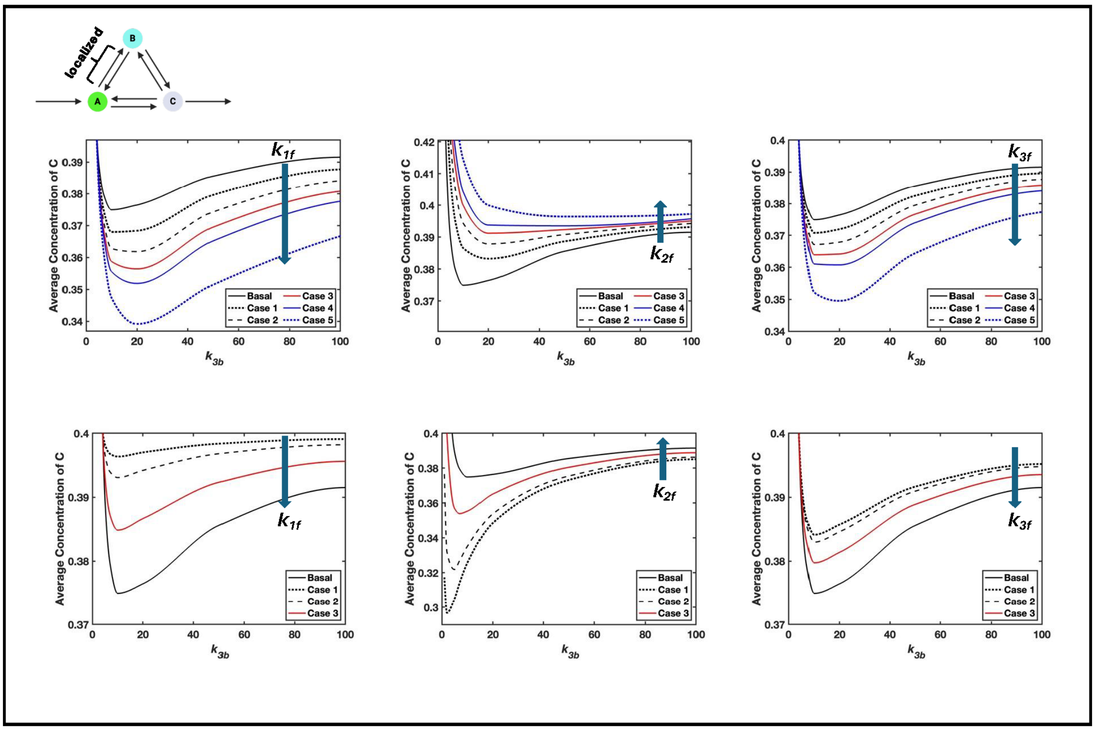

Figure S2: Sensitivity of biphasic behaviour to kinetic parameters. We consider the biphasic behaviour seen in Fig. 2(f) of the main text, and examines the effecct of variation of parameters. In the top row, left to right, we consider the effect of an increase of three different parameters: k_{1f} (rate constant associated with A to B conversion), k_{2f} (rate constant associated with B to C conversion), k_{3f} (rate constant associated with C to A conversion): case 1, case 2, case 3, case 4 and case 5 correspond to 1.5, 2, 2.5, 3 and 5 fold increase of the basal parameter, respectively. In the bottom row, Case 1, Case 2 and Case 3 correspond to a 10 fold, 5 fold and 2 fold decrease relative to basal values. As is seen in the figure, biphasic responses continue to be observed.

Metabolic Control Coefficients

Here, we present the remaining standard control coefficients for the 2-node 1 inflow 2 outflow case. Due to the large number of coefficients generated, in the main text we presented a selection, The remaining coefficients are shown in figure S3. Furthermore, for both the 2-node and the 3-node, we selected one control coefficient and dissected the 3D plot into five plots, each plot representing the control coefficient at a compartment size, see figures S4&S5. The plots are organized progressively increasing in size starting at 20% compartment size to a 100% compartment size.
The spatial control coefficients were also generated for two different 2-node cases that could be seen as the intermediate cases of the two motifs presented in the main text (Fig. 6 Table 1 and Table 2). The tables are highlighted in S6.

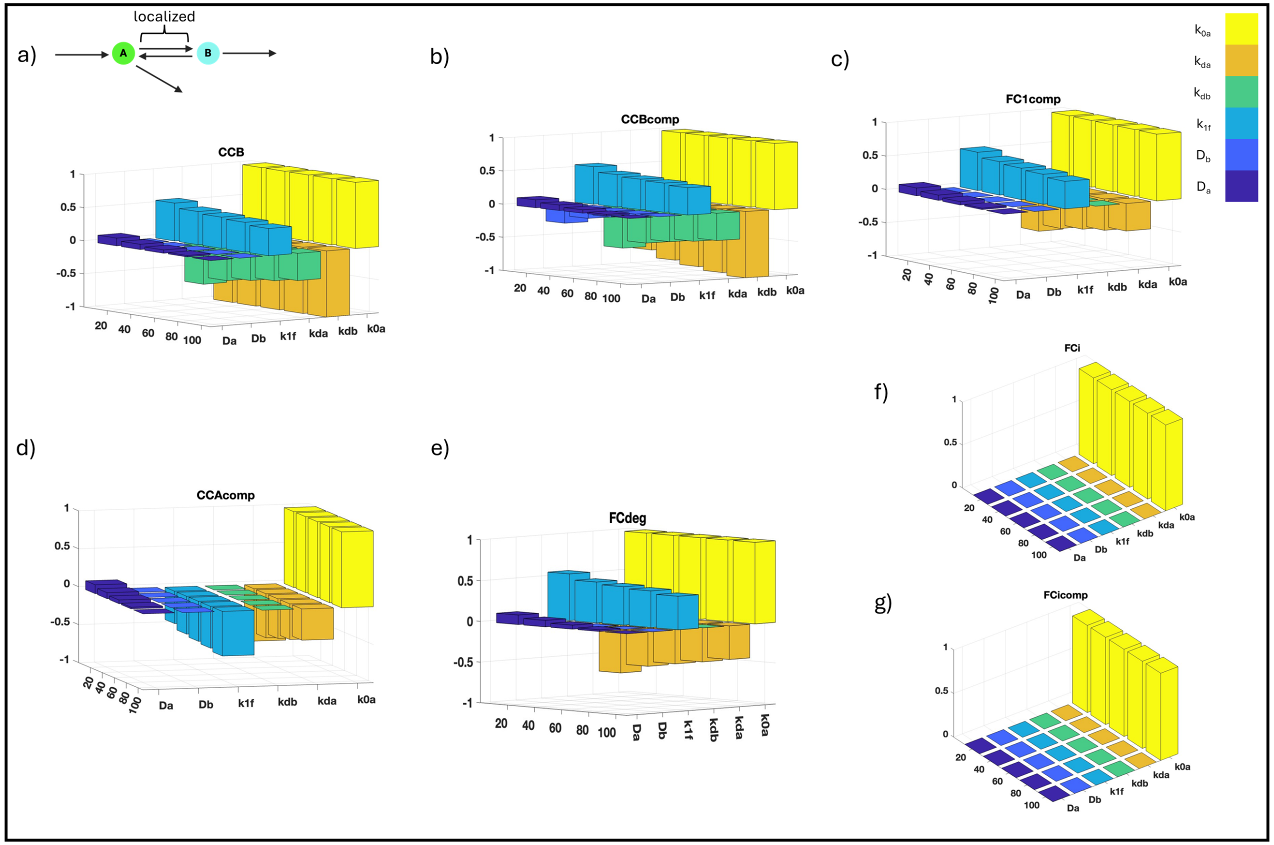

Figure S3: Additional control coefficients for fluxes and concentrations in the 2-node 1 inflow 2 outflow reversible system with localized interconversion(Created with Biorender). All plots contain headings to identify the type of control coefficient it represents

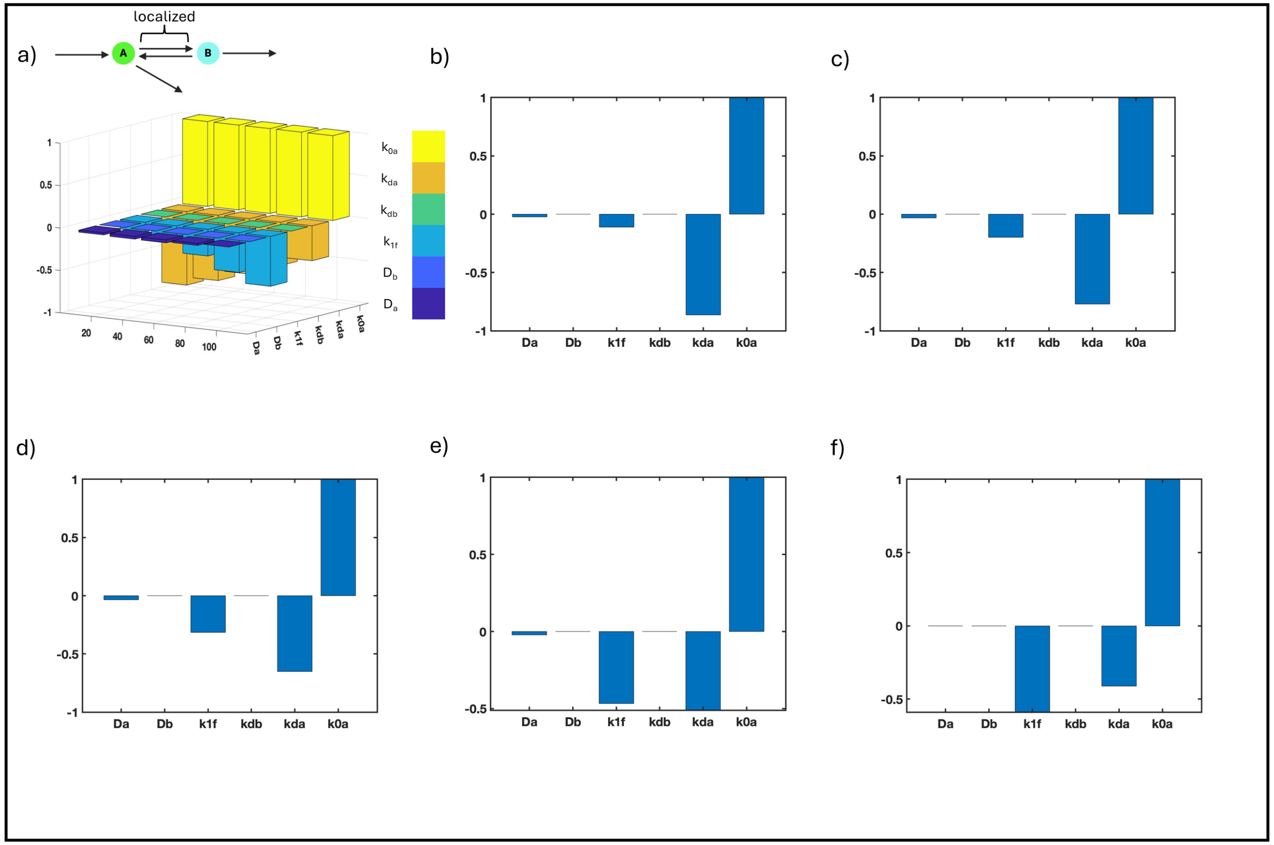

Figure S4: 2D cross sections of the control coefficient of domain averaged concentration of species A, for the 2-node 1 inflow 2 outflow system with localized interconversion(Created with Biorender). The plots are organized progressively increasing in compartment size (b-f) starting at 20% compartment size and progressing to a 100% compartment size.

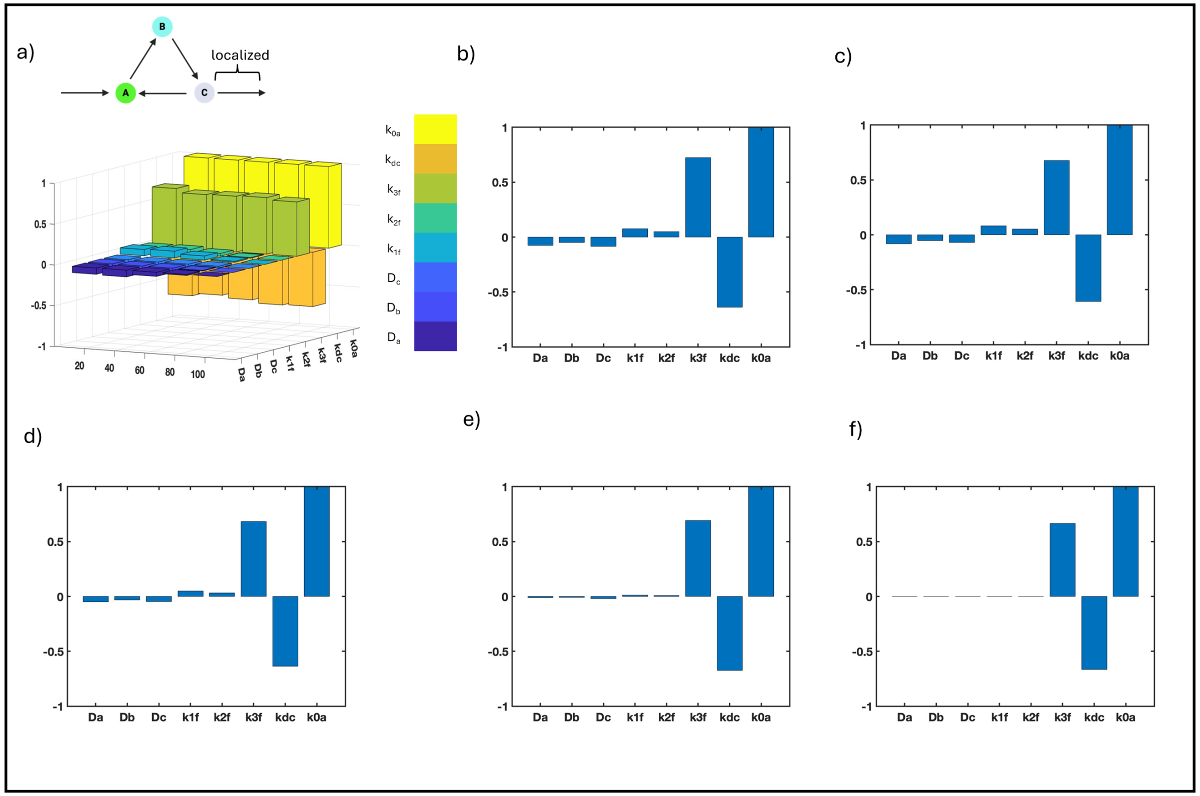

Figure S5: 2D cross sections of the domain averaged flux control coefficient (over the full domain) for the B to C reaction of the 3-node system(Created with Biorender). The plots (b-f) are organized progressively increasing in size of localization, starting at 20% compartment size and progressing to a 100% compartment size.

Table S1: Spatial control coefficients for the following system: 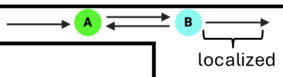

Spatial Control Coefficient (CC) for either the full domain or the compartment	CC_A	CC_B	FC_i	FC_1	FC_2	FC_{deg}
Scenario 1: Location CC for a compartment of size 20% of the domain						
Domain CC	-0.00882	-0.00941	0.0	-0.00882	-0.00941	-0.00941
Compartment CC	0.0	0.0	0.0	0.0	0.0	0.0
Scenario 2: Location CC for a compartment of size 10% of the domain						
Domain CC	-0.00516	-0.00535	0.00000	-0.00516	-0.00535	-0.00535
Compartment CC	0.0	0.0	0.0	0.0	0.0	0.0
Scenario 3: Size CC for a compartment of size 20% localized in the middle of the domain						
Domain CC	-0.82	-0.87	0.00	-0.82	-0.87	-0.87
Compartment CC	-0.91	-1.00	0.00	-0.91	-1.00	-1.00
Scenario 4: same as scenario 3 but localized on the side of the domain						
Domain CC	-0.72	-0.75	0.00	-0.72	-0.75	-0.75
Compartment CC	-0.95	-1.00	0.00	-0.95	-1.00	-1.00
Scenario 5: Same as scenario 3 but the enzymes are adjusted with the size impacts						
Domain CC	-0.18	-0.19	0.00	-0.18	-0.19	-0.19
Compartment CC	-0.11	0.01	0.00	-0.11	0.01	0.01

Table S2: Spatial control coefficients for the following system: 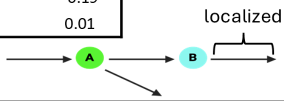

Spatial Control Coefficient (CC) for either the full domain or the compartment	CC_A	CC_B	FC_i	FC_1	FC_{degA}	FC_{degB}
Scenario 1: Location CC for a compartment of size 20% of the domain						
Domain CC	0	-0.0185	0	0	0	-0.0185
Compartment CC	0	0	0	0	0	0
Scenario 2: Location CC for a compartment of size 10% of the domain						
Domain CC	0	-0.0118	0	0	0	-0.0118
Compartment CC	0	0	0	0	0	0
Scenario 3: Size CC for a compartment of size 20% localized in the middle of the domain						
Domain CC	0	-0.76	0	0	0	-0.76
Compartment CC	0	-1.00	0	0	0	-1.00
Scenario 4: same as scenario 3 but localized on the side of the domain						
Domain CC	0	-0.68	0	0	0	-0.68
Compartment CC	0	-1.00	0	0	0	-1.00
Scenario 5: Same as scenario 3 but the enzymes are adjusted with the size impacts						
Domain CC	0	-0.27	0	0	0	-0.27
Compartment CC	0	0.01	0	0	0	0.01

Figure S6: Tables S1& S2 in the figure present the spatial control coefficients for the location and the size of compartment for a 2-node 1 inflow 1 outflow reversible motif and a 2-node 1 inflow 2 outflow irreversible motif, respectively (Created with Biorender). In each table, 5 different scenarios are presented. Two of them (1,2) show the location control coefficient for different sizes of the compartment. The subsequent three (3-5) depict different size control coefficients. (3) is based on a compartment centred in the middle of the spatial domain. (4) is based on a compartment asymmetrically placed with respect to the centre of the domain. (5) evaluates the control coefficient for the case of scaling the concentration of enzymes inversely with the size of the compartment to keep total amount fixed (see text). For each scenario, two rows depict the cases where the output measure (whether concentration or flux) is averaged either over the full spatial domain, or over the compartment. The different columns denote the specific output (concentrations of specific species or fluxes of particular reactions). CC_A, CC_B denote the control coeffcients for concentrations of A and B, while FC_i, FC_1, FC_{deg} denotes coefficients associated with the influx to A, the A to B conversion, and the efflux of B respectively. Additionally $FC2, FC_{degA}, FC_{degB}$ in Table 2 denote coefficients associated with flux from B to A, efflux of A and efflux of B respectively

Table S3: Evaluating the changes in the average concentration over the full domain and the compartment after localizing specific enzymes for a 2 node 1 input 2 output system

Localization	A	B	A_{comp}	B_{comp}
$E_{Deg\ B}$	6%	45%	-6%	0%
$E_{A\ to\ B}$	9%	-84%	-11%	-42%
$E_{B\ to\ A}$	-44%	31%	-25%	-5%
$E_{Deg\ A}$	62%	43%	19%	12%

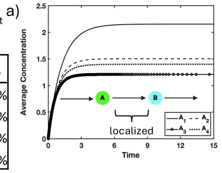

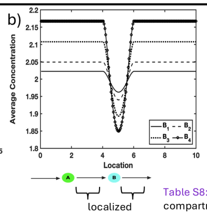

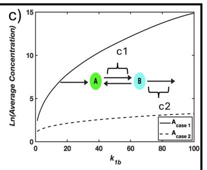

Table S4: Evaluating the changes in the average concentration over the full domain and the compartment after localizing specific enzymes for a 3 node 1 input 2 output cyclic system

Localization	A	B	C	A_{comp}	B_{comp}	C_{comp}
$E_{Deg\ B}$	80%	252%	252%	60%	160%	173%
$E_{A\ to\ B}$	2540%	71%	71%	511%	495%	427%
$E_{B\ to\ c}$	-19%	161%	-61%	12%	96%	52%
$E_{C\ to\ A}$	-20%	12%	162%	15%	49%	82%
$E_{Deg\ C}$	81%	153%	254%	55%	125%	152%

Table S5: Evaluating the changes in the average concentration over the full domain and the compartment after localizing specific enzymes for a 3 node 1 input 2 output cyclic reversible system

Localization	A	B	C	A_{comp}	B_{comp}	C_{comp}
$E_{Deg\ B}$	88%	193%	124%	41%	70%	57%
$E_{A\ to\ B}$	92%	-27%	51%	24%	9%	23%
$E_{B\ to\ c}$	-5%	16%	-30%	0%	4%	-5%
$E_{C\ to\ A}$	-27%	-10%	18%	-4%	-2%	3%
$E_{B\ to\ A}$	-25%	7%	-14%	-4%	1%	-4%
$E_{C\ to\ B}$	8%	-25%	46%	5%	0%	13%
$E_{A\ to\ C}$	63%	23%	-43%	16%	12%	2%
$E_{Deg\ C}$	28%	40%	59%	16%	22%	24%

Table S6: Impacts of changing the location of the compartment on the average concentrations for different enzyme localizations of a 2 node 1 input 2 output system

Localization	A	B	A_{comp}	B_{comp}
$E_{Deg\ A}$	5.86%	5.86%	-18.94%	-20.16%
$E_{Deg\ B}$	1.46%	2.65%	-9.10%	-14.13%
$E_{A\ to\ B}$	0.60%	-7.09%	-7.24%	9.06%
$E_{B\ to\ A}$	-0.89%	0.76%	4.90%	-6.59%

Table S7: Impacts of splitting a compartment into two equally sized on the average concentrations for different enzyme localizations of a 2 node 1 input 2 output system

Localization	A	B	A_{comp}	B_{comp}
$E_{Deg\ A}$	-4.56%	-4.59%	14.87%	13.48%
$E_{Deg\ B}$	-1.19%	-2.16%	3.90%	11.46%
$E_{A\ to\ B}$	-0.38%	5.07%	4.95%	-23.81%
$E_{B\ to\ A}$	-2.07%	-1.14%	-9.63%	9.35%

Table S8: Impacts of changing the location of the compartment on the average concentrations for different enzyme localizations of a 3 node 1 input 2 output cyclic reversible system

Concentration	$E_{Deg\ C}$	$E_{C\ to\ A}$	B&C
A	1.16%	0.05%	135.62%
B	1.42%	0.00%	
C	1.71%	-0.05%	
A_{comp}	-7.55%	3.62%	0.73%
B_{comp}	-9.86%	2.74%	0.74%
C_{comp}	-10.67%	0.11%	0.73%

Table S9: Impacts of splitting a compartment into two equally sized on the average concentrations for different enzyme localizations of a 3 node 1 input 2 output cyclic reversible system

Concentration	$E_{Deg\ C}$	$E_{C\ to\ A}$	B&C
A	-0.95%	-0.05%	-27.01%
B	-1.19%	0.00%	
C	-1.41%	0.00%	
A_{comp}	4.67%	-7.83%	0.55%
B_{comp}	6.40%	-5.28%	0.71%
C_{comp}	8.76%	-0.63%	0.70%

∞

Figure S7: Tables S(3-5) show the percentage change in the spatial average concentration over both the full domain and the compartment for the various species when compared to a non-localized system, for three different motifs (all with one inflow and two outflows): Table 3: 2 node motif, Table 4: 3 node cyclic irreversible motif, and Table 5: 3 node reversible cyclic motif. Tables (6-9) show the percentage change in the spatial average concentration over both the full domain and the compartment for different spatial alterations of compartments compared to a basal case of localization (compartment size 20% of the full domain, located in the middle). Tables 6 and 7 consider the two node motif presented in Table 3 and Tables 8 and 9 consider the three node motif presented in Table 5. In both sets of cases, the effect of altering the location of the compartment and the effect of splitting a compartment into two and symmetrically placing the resultant compartments about the midpoint is considered. Plots: (a) The average concentration of A in a 2 node 1 inflow 1 outflow system with localized interconversion enzyme plotted for four different enzymatic cases. A_1 is the basal case, A_2 is for two compartments half the size of the basal A_3 is for two compartments each the same size as the basal but with the same total amount of enzyme and A_4 is similar to A_3 but the enzyme is split 75% in one compartment and the rest in the second compartment. (b) The spatial profile of B in a 2 node 1 input 1 output system with two enzymes localized (conversion of A to B and the efflux of B) in the same compartment is plotted for different values of the interconversion rate constant ($k_1 f$). (c) The natural logarithm of the average concentration of B over the full domain plotted against $k_1 b$, the B to A reaction rate constant for a 2 node 1 outflow motif where the enzyme mediating conversion of A to B, and the enzyme mediating the efflux of B are localized in separate compartments, symmetrically about the middle of the domain.(Created with Biorender)

Supplementary Parameters

Biochemical Building Blocks

Figure S1
a)$k_{0a} = 2, E_a = 1, k_{1f} = 10, k_{1b} = 10, E_{ba} = 1, k_{da} = 0, E_{da} = 0, k_{0b} = 0, E_b = 0, k_{db} = 7, E_{db} = 1, k_{0C} = 0, E_c = 0, k_{2f} = 10, E_{bc} = 1, k_{2b} = 10, E_{cb} = 1, k_{3f} = 10, k_{3b} = 10, E_{ac} = 1, E_{ca} = 1, k_{dc} = 5, E_{dc} = 1, D_A = 9, D_C = 3, D_B = 1, 2, 5, 7, 10, 20, 50, 100, IC : A_0 = 0, B_0 = 0.C_0 = 0.E_{ab} = 1$at 20% of the middle of the domain.
b)$k_{0a} = 2, E_a = 1, k_{1f} = 10, k_{1b} = 10, E_{ba} = 1, k_{da} = 0, E_{da} = 0, k_{0b} = 0, E_b = 0, k_{db} = 7, E_{db} = 1, k_{0C} = 0, E_c = 0, E_{bc} = 1, k_{2b} = 10, E_{cb} = 1, k_{3f} = 10, k_{3b} = 10, E_{ac} = 1, E_{ab} = 1, k_{dc} = 5, E_{dc} = 1, D_A = 9, D_B = 5, D_C = 3, k_{2f} = 1, 2, 5, 7, 10, 20, 50, 100, IC : A_0 = 0, B_0 = 0.C_0 = 0.E_{ca} = 1$at 20% of the middle of the domain.
c)$k_{0a} = 2, E_a = 1, k_{1f} = 10, k_{1b} = 10, E_{ba} = 1, k_{da} = 0, E_{da} = 0, k_{0b} = 0, E_b = 0, k_{db} = 7, E_{ab} = 1, k_{0C} = 0, E_c = 0, k_{2f} = 10, E_{bc} = 1, k_{2b} = 10, E_{cb} = 1, k_{3f} = 10, k_{3b} = 10, E_{ac} = 1, E_{ca} = 1, k_{dc} = 5, E_{dc} = 1, D_A = 9, D_B = 5, D_C = 1, 2, 5, 7, 10, 20, 50, 100, IC : A_0 = 0, B_0 = 0.C_0 = 0.E_{db} = 1$at 20% of the middle of the domain.
d)$k_{0a} = 2, E_a = 1, k_{1f} = 10, k_{1b} = 10, E_{ba} = 1, k_{da} = 0, E_{da} = 0, k_{0b} = 0, E_b = 0, k_{db} = 7, E_{db} = 1, k_{0C} = 0, E_c = 0, k_{2f} = 10, E_{bc} = 1, k_{2b} = 10, E_{cb} = 1, k_{3f} = 10, E_{ac} = 1, E_{ca} = 1, k_{dc} = 5, E_{dc} = 1, D_A = 9, D_B = 5, D_C = 3, k_{3b} = 1, 2, 5, 7, 10, 20, 50, 100, IC : A_0 = 0, B_0 = 0.C_0 = 0.E_{ab} = 1$at 20% of the middle of the domain.
e) The case from (a) with $D_B = 5$, the case from (b) with $k_{2f} = 10$ and the case from (c) with $D_C = 3$

Metabolic Control Coefficients

Figure S3-S6
Same parameters used in Figure 5 (a-d).

Engineering Perspective

Figure S7
a) $k_{0a} = 2, E_a = 1, k_{1f} = 10, E_{ba} = 0, k_{da} = 0, E_{da} = 0, k_{0b} = 0, E_b = 0, k_{db} = 5, E_{db} = 1, D_A = 9, D_B = 5, IC : A_0 = 0, B_0 = 0.$
For this case, 4 different enzymatic conditions were applied

1. $E_{ab} = 1$at 20% of the middle of the domain.
2. $E_{ab} = 0.5$, in two compartments each 20% of the full domain, Compartment 1 occupies $21\% - 40\%$ of the domain, while compartment 2 occupies $61\% - 80\%$ of the domain.
3. $E_{ab} = 1$in two compartments each 10% of the full domain, Compartment 1 occupies $31\% - 40\%$ of the domain, while compartment 2 occupies $61\% - 70\%$ of the domain.
4. $E_{ab} = 0.75$ in compartment 1, on the other hand $E_{ab} = 0.25$ in compartment 2, the two compartments are each 20% of the full domain, Compartment 1 occupies $21\% - 40\%$ of the domain, while compartment 2 occupies $61\% - 80\%$ of the domain.

b)$k_{0a} = 2, E_a = 1, k_{1f} = 10, 20, 50, 100, E_{ba} = 0, k_{1b} = 0, k_{da} = 0, E_{da} = 0, k_{0b} = 0, E_b = 0, k_{db} = 5, D_A = 9, D_B = 5, IC : A_0 = 0, B_0 = 0.E_{ab} = E_{db} = 1$ both at 20% of the middle of the domain.
c)$k_{0a} = 2, E_a = 1, k_{1f} = 10, E_{ba} = 1, k_{da} = 0, E_{da} = 0, k_{0b} = 0, E_b = 0, k_{db} = 5, D_A = 9, D_B = 5, k_{1b} = 1, 2, 5, 7, 10, 20, 50, 100, IC : A_0 = 0, B_0 = 0.$
This case has two enzymatic localization at different locations both are 20% of the size of the full domain

1. $E_{ab} = 1$ at compartment 1 occupying $21\% - 40\%$ of the domain.
2. $E_{db} = 1$ at compartment 1 occupying $61\% - 80\%$ of the domain.

Tables S3-S9 involve three motifs (with two outflows) which are studied previously; nominal motif parameters: A 2-node reversible motif studied in Fig. 5 (a-d), a three node irreversible motif studied in Fig. 5(e,f), but with an efflux for B added (same rate constant as efflux of C), and a three node reversible motif studied: parameters same as the irreversible three node motif, but with forward and backward interconversion rate constants equal.

Methodology Figures

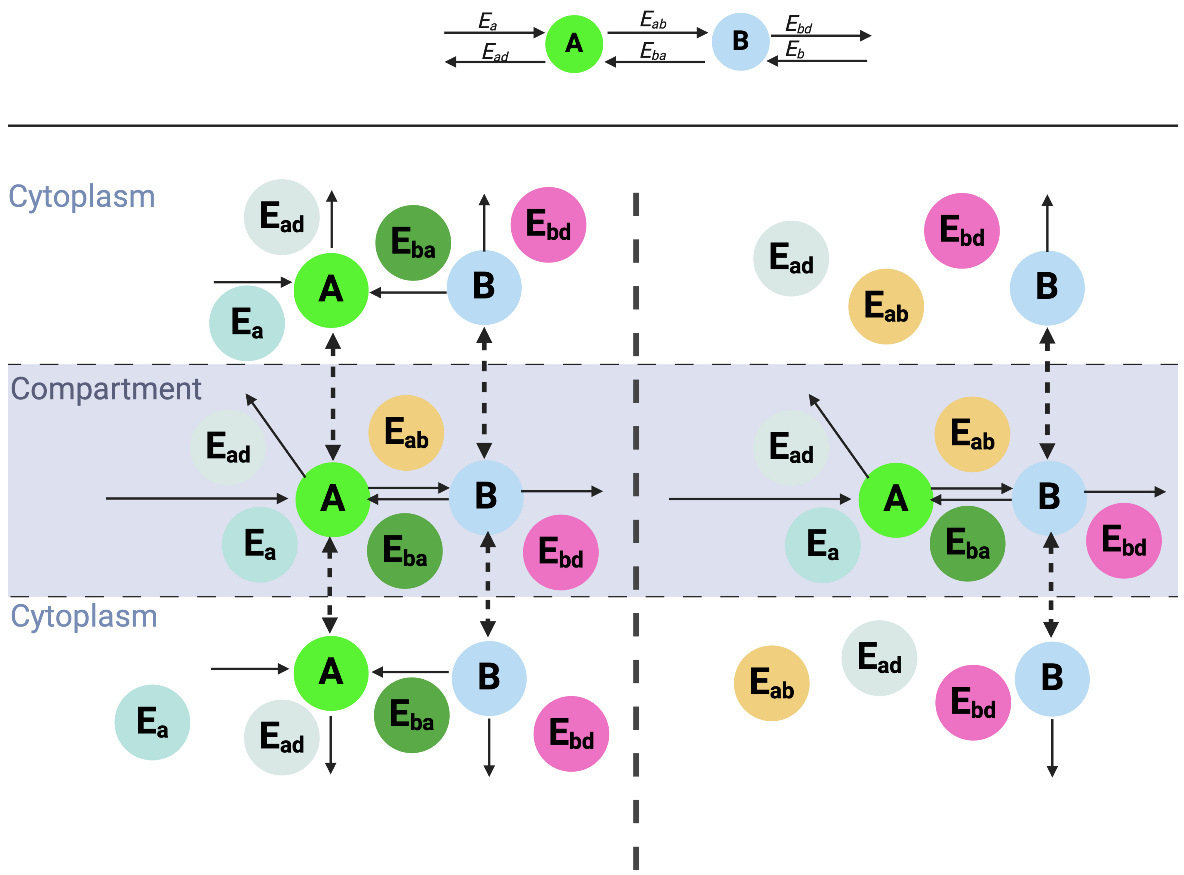

Figure S8: Schematic of a typical 2-node motif with all possible reactions and designated enzymes (Created with Biorender).This is also shown in the spatial context. The left panel highlights the enzyme localized scenario (the enzyme being localized is the enzyme mediating the conversion from B to A). The right panel shows the substrate localization scenario. A is the substrate being localized. Note that localization of a substrate requires localization of enzymes which produce it.

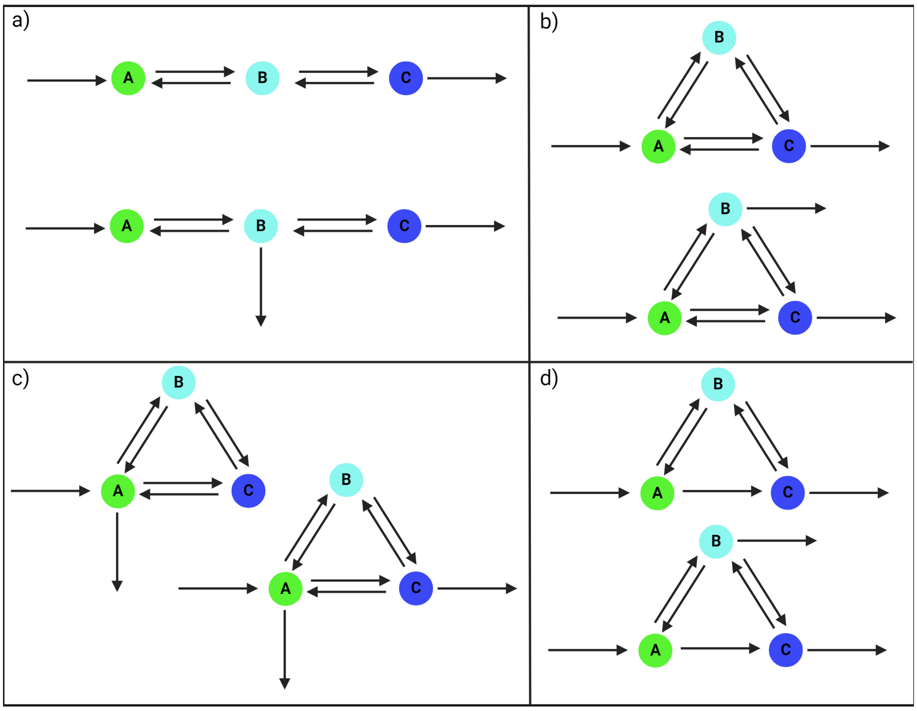

Figure S9: Schematics of different types of 3-node motifs evaluated for either a 1 inflow 1 outflow or 1 inflow 2 outflow schemes (Created with Biorender). a)Linear structures with one and 2 outflows. b)The fully reversible cyclic motif with one and two outflows. c) A specific case of reversible cyclic motifs with one and two outflows, where A is one of the outflow nodes, d) Partly reversible cyclic motifs with one and two outflows.

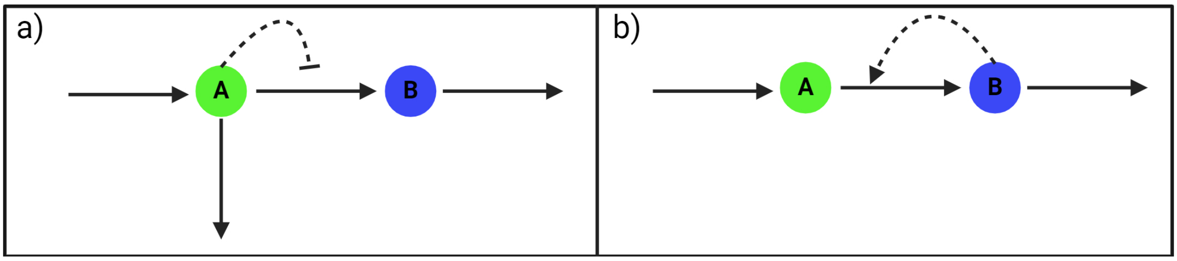

Figure S10: Schematic of the biochemical reaction description for the motifs with feedback(Created with Biorender). a) A 2-node system that generates bistability, where A inhibits its conversion to B. b)A 2-node system that generates oscillations. This system was found to generate oscilaltions under two different mathematical descriptions (see Models). Here B activates its own conversion for A.

Exemplar Study: Glycolysis Model

Smallbone et al. modelled and studied the yeast glycolysis pathway using information from the literature Their model is calibrated against experimental data. The model has a fixed import flux of glucose and that of Ethanol. The reaction rates did not simply follow a Michaelis Menten kinetics. For example, TPI had shown inhibition utilizing a hill type function with coefficient of 4 [?]. The model contains multiple interactions in which feedback occurs. Additionally, the model considered the different enzymatic forms with their evaluated concentrations. The schematic of the model only sketches out broad aspects of the underlying network without indicating many underlying details he details of this model and assumptions are presented in the associated publications.

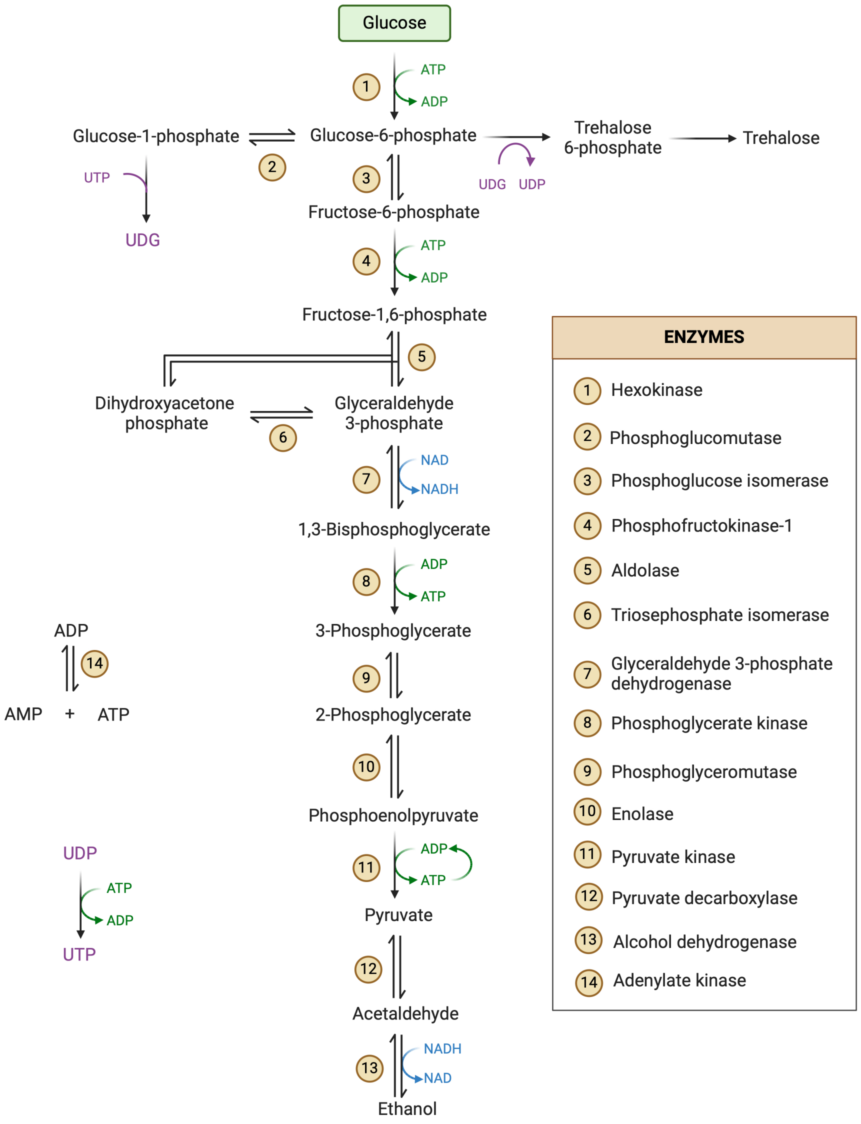

Figure S11: Glycolysis Pathway model Created with Biorender

Exemplar model of Yeast Glycolysis

1 Rate Laws

$$v_{\text{acetate branch}} = k_{\text{acetate branch}} \cdot [\text{acetaldehyde}] \cdot [\text{NAD}]$$

$$v_{\text{ADH1}} = [\text{ADH1}] \cdot k_{\text{cat}}(\text{ADH1}) \cdot \left(\frac{[\text{acetaldehyde}] \cdot [\text{NADH}]}{K_{\text{acald}}(\text{ADH1}) \cdot K_{\text{nadh}}(\text{ADH1})} - \frac{[\text{ethanol}] \cdot [\text{NAD}]}{K_{\text{etoh}}(\text{ADH1}) \cdot K_{\text{nad}}(\text{ADH1}) \cdot K_{\text{eq ADH}}} \right)$$
$$\cdot \frac{1}{1 + \frac{[\text{acetaldehyde}]}{K_{\text{acald}}(\text{ADH1})} + \frac{[\text{NADH}]}{K_{\text{nadh}}(\text{ADH1})} + \frac{[\text{acetaldehyde}] \cdot [\text{NADH}]}{K_{\text{acald}}(\text{ADH1}) \cdot K_{\text{nadh}}(\text{ADH1})}}$$
$$+ \frac{[\text{ethanol}]}{K_{\text{etoh}}(\text{ADH1})} + \frac{[\text{NAD}]}{K_{\text{nad}}(\text{ADH1})} + \frac{[\text{ethanol}] \cdot [\text{NAD}]}{K_{\text{etoh}}(\text{ADH1}) \cdot K_{\text{nad}}(\text{ADH1})}$$
$$+ \frac{[\text{acetaldehyde}] \cdot [\text{NAD}]}{K_{\text{acald}}(\text{ADH1}) \cdot K_{\text{nad}}(\text{ADH1})} + \frac{[\text{ethanol}] \cdot [\text{NADH}]}{K_{\text{etoh}}(\text{ADH1}) \cdot K_{\text{nadh}}(\text{ADH1})} + \frac{[\text{acetaldehyde}] \cdot [\text{ethanol}]}{K_{\text{acald}}(\text{ADH1}) \cdot K_{\text{etoh}}(\text{ADH1})}$$
$$+ \frac{[\text{acetaldehyde}] \cdot [\text{ethanol}] \cdot [\text{NAD}]}{K_{\text{acald}}(\text{ADH1}) \cdot K_{\text{etoh}}(\text{ADH1}) \cdot K_{\text{nad}}(\text{ADH1})}$$

$$v_{\text{ADH5}} = [\text{ADH5}] \cdot k_{\text{cat}}(\text{ADH5}) \cdot \left(\frac{[\text{acetaldehyde}] \cdot [\text{NADH}]}{K_{\text{acald}}(\text{ADH5}) \cdot K_{\text{nadh}}(\text{ADH5})} - \frac{[\text{ethanol}] \cdot [\text{NAD}]}{K_{\text{etoh}}(\text{ADH5}) \cdot K_{\text{nad}}(\text{ADH5}) \cdot K_{\text{eq ADH}}} \right)$$
$$\cdot \frac{1}{1 + \frac{[\text{acetaldehyde}]}{K_{\text{acald}}(\text{ADH5})} + \frac{[\text{NADH}]}{K_{\text{nadh}}(\text{ADH5})} + \frac{[\text{acetaldehyde}] \cdot [\text{NADH}]}{K_{\text{acald}}(\text{ADH5}) \cdot K_{\text{nadh}}(\text{ADH5})}}$$
$$+ \frac{[\text{ethanol}]}{K_{\text{etoh}}(\text{ADH5})} + \frac{[\text{NAD}]}{K_{\text{nad}}(\text{ADH5})} + \frac{[\text{ethanol}] \cdot [\text{NAD}]}{K_{\text{etoh}}(\text{ADH5}) \cdot K_{\text{nad}}(\text{ADH5})}$$
$$+ \frac{[\text{acetaldehyde}] \cdot [\text{NAD}]}{K_{\text{acald}}(\text{ADH5}) \cdot K_{\text{nad}}(\text{ADH5})} + \frac{[\text{ethanol}] \cdot [\text{NADH}]}{K_{\text{etoh}}(\text{ADH5}) \cdot K_{\text{nadh}}(\text{ADH5})} + \frac{[\text{acetaldehyde}] \cdot [\text{ethanol}]}{K_{\text{acald}}(\text{ADH5}) \cdot K_{\text{etoh}}(\text{ADH5})}$$
$$+ \frac{[\text{acetaldehyde}] \cdot [\text{ethanol}] \cdot [\text{NAD}]}{K_{\text{acald}}(\text{ADH5}) \cdot K_{\text{etoh}}(\text{ADH5}) \cdot K_{\text{nad}}(\text{ADH5})}$$

$$v_{\text{AK}} = k_{\text{AK}} \cdot \left(\frac{[\text{ADP}] \cdot [\text{ADP}] - [\text{AMP}] \cdot [\text{ATP}]}{K_{\text{eq}}(\text{AK})} \right)$$

$$v_{\text{ATPase}} = V_{\text{max}}(\text{ATPase}) \cdot \frac{[\text{ATP}]}{K_{\text{atp}}(\text{ATPase}) \left(1 + \frac{[\text{ATP}]}{K_{\text{atp}}(\text{ATPase})} \right)}$$

$$v_{\text{CDC19}} = [\text{CDC19}] \cdot k_{\text{cat}}(\text{CDC19}) \cdot \left(\frac{[\text{phosphoenolpyruvate}] \cdot [\text{ADP}] - \frac{[\text{pyruvate}] \cdot [\text{ATP}]}{K_{\text{eq PYK}}}}{K_{\text{pep}}(\text{CDC19}) \cdot K_{\text{adp}}(\text{CDC19})} \right)$$
$$\cdot \frac{1}{\left(1 + \frac{[\text{phosphoenolpyruvate}]}{K_{\text{pep}}(\text{CDC19})} + \frac{[\text{pyruvate}]}{K_{\text{pyr}}(\text{CDC19})} + L_0(\text{CDC19}) \cdot \frac{\frac{[\text{ATP}]}{K_{\text{iatp}}(\text{CDC19})} + 1}{1 + \frac{[\text{fructose 1,6-bisphosphate}]}{K_{\text{f16p}}(\text{CDC19})}} \right)}$$
$$\cdot \left(1 + \frac{[\text{ADP}]}{K_{\text{adp}}(\text{CDC19})} + \frac{[\text{ATP}]}{K_{\text{atp}}(\text{CDC19})} \right)$$

$$v_{\text{ENO1}} = [\text{ENO1}] \cdot k_{\text{cat}}(\text{ENO1}) \cdot \left(\frac{[\text{2-phosphoglycerate}]}{K_{\text{p2g}}(\text{ENO1})} - \frac{[\text{phosphoenolpyruvate}]}{K_{\text{p2g}}(\text{ENO1}) \cdot K_{\text{eq ENO}}} \right)$$
$$\cdot \left(\frac{1}{1 + \frac{[\text{2-phosphoglycerate}]}{K_{\text{p2g}}(\text{ENO1})} + \frac{[\text{phosphoenolpyruvate}]}{K_{\text{pep}}(\text{ENO1})}} \right)$$

$$v_{\text{ENO2}} = [\text{ENO2}] \cdot k_{\text{cat}}(\text{ENO2}) \cdot \left(\frac{[\text{2-phosphoglycerate}]}{K_{\text{p2g}}(\text{ENO2})} - \frac{[\text{phosphoenolpyruvate}]}{K_{\text{p2g}}(\text{ENO2}) \cdot K_{\text{eq ENO}}} \right)$$
$$\cdot \left(\frac{1}{1 + \frac{[\text{2-phosphoglycerate}]}{K_{\text{p2g}}(\text{ENO2})} + \frac{[\text{phosphoenolpyruvate}]}{K_{\text{pep}}(\text{ENO2})}} \right)$$

$$v_{\text{FBA}} = [\text{FBA1}] \cdot k_{\text{cat}}(\text{FBA}) \cdot \left(\frac{[\text{fructose 1,6-bisphosphate}]}{K_{\text{f16bp}}(\text{FBA})} - \frac{[\text{dihydroxyacetone phosphate}] \cdot [\text{glyceraldehyde 3-phosphate}]}{K_{\text{f16bp}}(\text{FBA}) \cdot K_{\text{eq FBA}}} \right)$$
$$\cdot \frac{1}{1 + \frac{[\text{fructose 1,6-bisphosphate}]}{K_{\text{f16bp}}(\text{FBA})} + \frac{[\text{dihydroxyacetone phosphate}]}{K_{\text{dhap}}(\text{FBA})} + \frac{[\text{glyceraldehyde 3-phosphate}]}{K_{\text{gap}}(\text{FBA})} + \frac{[\text{dihydroxyacetone phosphate}] \cdot [\text{glyceraldehyde 3-phosphate}]}{K_{\text{dhap}}(\text{FBA}) \cdot K_{\text{gap}}(\text{FBA})} + \frac{[\text{fructose 1,6-bisphosphate}] \cdot [\text{dihydroxyacetone phosphate}]}{K_{\text{f16bp}}(\text{FBA}) \cdot K_{\text{dhap}}(\text{FBA})}}$$

$$v_{\text{GLK1}} = [\text{GLK1}] \cdot k_{\text{cat}}(\text{GLK1}) \cdot \left(\frac{[\text{glucosecell}] \cdot [\text{ATP}]}{K_{\text{glc}}(\text{GLK1}) \cdot K_{\text{atp}}(\text{GLK1})} - \frac{[\text{glucose 6-phosphate}] \cdot [\text{ADP}]}{K_{\text{g6p}}(\text{GLK1}) \cdot K_{\text{adp}}(\text{GLK1}) \cdot K_{\text{eq HXK}}} \right)$$
$$\cdot \frac{1}{\left(1 + \frac{[\text{glucosecell}]}{K_{\text{glc}}(\text{GLK1})} + \frac{[\text{glucose 6-phosphate}]}{K_{\text{g6p}}(\text{GLK1})} \right) \cdot \left(1 + \frac{[\text{ATP}]}{K_{\text{atp}}(\text{GLK1})} + \frac{[\text{ADP}]}{K_{\text{adp}}(\text{GLK1})} \right)}$$

$$v_{\text{Glycerol3P}} = V_{\text{max}}(\text{Glycerol3P}) \cdot \frac{[\text{glycerol 3-phosphate}]}{K_{\text{g3p}}(\text{Glycerol3P}) \left(1 + \frac{[\text{glycerol 3-phosphate}]}{K_{\text{g3p}}(\text{Glycerol3P})} \right)}$$

$$v_{\text{Glycerol3PDH}} = \frac{V_{\text{max}}(\text{Glycerol3PDH})}{K_{\text{dhap}}(\text{Glycerol3PDH}) \cdot K_{\text{nadh}}(\text{Glycerol3PDH})} \cdot \left([\text{dihydroxyacetone phosphate}] \cdot [\text{NADH}] - \frac{[\text{glycerol 3-phosphate}] \cdot [\text{NAD}]}{K_{\text{eq}}(\text{Glycerol3PDH})} \right)$$
$$\cdot \frac{1}{\left(1 + \frac{[\text{fructose 1,6-bisphosphate}]}{K_{\text{fbp}}(\text{Glycerol3PDH})} + \frac{[\text{ATP}]}{K_{\text{atp}}(\text{Glycerol3PDH})} + \frac{[\text{ADP}]}{K_{\text{adp}}(\text{Glycerol3PDH})} \right)}$$
$$\cdot \frac{1}{\left(1 + \frac{[\text{dihydroxyacetone phosphate}]}{K_{\text{dhap}}(\text{Glycerol3PDH})} + \frac{[\text{glycerol 3-phosphate}]}{K_{\text{g3p}}(\text{Glycerol3PDH})} \right)}$$
$$\cdot \frac{1}{\left(1 + \frac{[\text{NADH}]}{K_{\text{nadh}}(\text{Glycerol3PDH})} + \frac{[\text{NAD}]}{K_{\text{nad}}(\text{Glycerol3PDH})} \right)}$$

$$v_{\text{GPM1}} = [\text{GPM1}] \cdot k_{\text{cat}}(\text{GPM1}) \cdot \left(\frac{[\text{3-phosphoglycerate}]}{K_{\text{p3g}}(\text{GPM1})} - \frac{[\text{2-phosphoglycerate}]}{K_{\text{p3g}}(\text{GPM1}) \cdot K_{\text{eq GPM1}}} \right)$$

$$\cdot\left(\frac{1}{1+\frac{[\text{3-phosphoglycerate}]}{K_{\text{p3g}}(\text{GPM1})}+\frac{[\text{2-phosphoglycerate}]}{K_{\text{p2g}}(\text{GPM1})}}\right)$$

$$v_{\text{HXK1}}=\frac{[\text{HXK1}]\cdot k_{\text{cat}}(\text{HXK1})\cdot\left(\frac{[\text{glucosecell}]\cdot[\text{ATP}]}{K_{\text{glc}}(\text{HXK1})\cdot K_{\text{atp}}(\text{HXK1})}-\frac{[\text{glucose 6-phosphate}]\cdot[\text{ADP}]}{K_{\text{glc}}(\text{HXK1})\cdot K_{\text{atp}}(\text{HXK1})\cdot K_{\text{eq HXK}}}\right)}{\left(1+\frac{[\text{glucosecell}]}{K_{\text{glc}}(\text{HXK1})}+\frac{[\text{glucose 6-phosphate}]}{K_{\text{g6p}}(\text{HXK1})}+\frac{[\text{trehalose 6-phosphate}]}{K_{\text{t6p}}(\text{HXK1})}\right)\cdot\left(1+\frac{[\text{ATP}]}{K_{\text{atp}}(\text{HXK1})}+\frac{[\text{ADP}]}{K_{\text{adp}}(\text{HXK1})}\right)}$$

$$v_{\text{HXK2}}=\frac{[\text{HXK2}]\cdot k_{\text{cat}}(\text{HXK2})\cdot\left(\frac{[\text{glucosecell}]\cdot[\text{ATP}]}{K_{\text{glc}}(\text{HXK2})\cdot K_{\text{atp}}(\text{HXK2})}-\frac{[\text{glucose 6-phosphate}]\cdot[\text{ADP}]}{K_{\text{glc}}(\text{HXK2})\cdot K_{\text{atp}}(\text{HXK2})\cdot K_{\text{eq HXK}}}\right)}{\left(1+\frac{[\text{glucosecell}]}{K_{\text{glc}}(\text{HXK2})}+\frac{[\text{glucose 6-phosphate}]}{K_{\text{g6p}}(\text{HXK2})}+\frac{[\text{trehalose 6-phosphate}]}{K_{\text{t6p}}(\text{HXK2})}\right)\cdot\left(1+\frac{[\text{ATP}]}{K_{\text{atp}}(\text{HXK2})}+\frac{[\text{ADP}]}{K_{\text{adp}}(\text{HXK2})}\right)}$$

$$v_{\text{HXT}}=V_{\max}(\text{HXT})\cdot\frac{[\text{glucoseextracellular}]-[\text{glucosecell}]}{K_{\text{glc}}(\text{HXT})\left(1+\frac{[\text{glucoseextracellular}]}{K_{\text{glc}}(\text{HXT})}+\frac{[\text{glucosecell}]}{K_{\text{glc}}(\text{HXT})}+K_i(\text{HXT})\cdot\frac{[\text{glucoseextracellular}]}{K_{\text{glc}}(\text{HXT})}\cdot\frac{[\text{glucosecell}]}{K_{\text{glc}}(\text{HXT})}\right)}$$

$$v_{\text{PDC1}}=[\text{PDC1}]\cdot k_{\text{cat}}(\text{PDC1})\cdot\frac{[\text{pyruvate}]}{K_{\text{pyr}}(\text{PDC1})\left(1+\frac{[\text{pyruvate}]}{K_{\text{pyr}}(\text{PDC1})}\right)}$$

$$v_{\text{PDC5}}=[\text{PDC5}]\cdot k_{\text{cat}}(\text{PDC5})\cdot\frac{[\text{pyruvate}]}{K_{\text{pyr}}(\text{PDC5})\left(1+\frac{[\text{pyruvate}]}{K_{\text{pyr}}(\text{PDC5})}\right)}$$

$$v_{\text{PDC6}}=[\text{PDC6}]\cdot k_{\text{cat}}(\text{PDC6})\cdot\frac{[\text{pyruvate}]}{K_{\text{pyr}}(\text{PDC6})\left(1+\frac{[\text{pyruvate}]}{K_{\text{pyr}}(\text{PDC6})}\right)}$$

$$v_{\text{PFK}}=\frac{\min([\text{PFK1}],[\text{PFK2}])\cdot k_{\text{cat}}(\text{PFK})\cdot g_R(\text{PFK})\cdot\frac{[\text{fructose 6-phosphate}]\cdot[\text{ATP}]}{K_{\text{f6p}}(\text{PFK})\cdot K_{\text{atp}}(\text{PFK})}\cdot\left(1-\frac{[\text{fructose 1,6-bisphosphate}]\cdot[\text{ADP}]}{[\text{fructose 6-phosphate}]\cdot[\text{ATP}]\cdot K_{\text{eq PFK}}}\right)\cdot\left(1+\frac{[\text{fructose 6-phosphate}]}{K_{\text{f6p}}(\text{PFK})}+\frac{g_R(\text{PFK})\cdot[\text{fructose 6-phosphate}]\cdot[\text{ATP}]}{K_{\text{f6p}}(\text{PFK})\cdot K_{\text{atp}}(\text{PFK})}+\frac{[\text{fructose 1,6-bisphosphate}]}{K_{\text{f16}}(\text{PFK})}+\frac{[\text{ADP}]}{K_{\text{adp}}(\text{PFK})}+\frac{[\text{ATP}]}{K_{\text{atp}}(\text{PFK})}+g_R(\text{PFK})\cdot\frac{[\text{fructose 1,6-bisphosphate}]\cdot[\text{ADP}]}{K_{\text{f16}}(\text{PFK})\cdot K_{\text{adp}}(\text{PFK})}\right)}{\left(1+\frac{[\text{fructose 6-phosphate}]}{K_{\text{f6p}}(\text{PFK})}+\frac{g_R(\text{PFK})\cdot[\text{fructose 6-phosphate}]\cdot[\text{ATP}]}{K_{\text{f6p}}(\text{PFK})\cdot K_{\text{atp}}(\text{PFK})}+\frac{[\text{fructose 1,6-bisphosphate}]}{K_{\text{f16}}(\text{PFK})}+\frac{[\text{ADP}]}{K_{\text{adp}}(\text{PFK})}+\frac{[\text{ATP}]}{K_{\text{atp}}(\text{PFK})}+g_R(\text{PFK})\cdot\frac{[\text{fructose 1,6-bisphosphate}]\cdot[\text{ADP}]}{K_{\text{f16}}(\text{PFK})\cdot K_{\text{adp}}(\text{PFK})}\right)^2+L0(\text{PFK})\cdot\left(\frac{\left(1+\frac{\text{Ciatp}(\text{PFK})\cdot[\text{ATP}]}{K_{\text{iatp}}(\text{PFK})}\right)}{1+\frac{[\text{ATP}]}{K_{\text{iatp}}(\text{PFK})}}\right)^2\cdot\left(\frac{\left(1+\frac{\text{Camp}(\text{PFK})\cdot[\text{AMP}]}{K_{\text{amp}}(\text{PFK})}\right)}{1+\frac{[\text{AMP}]}{K_{\text{amp}}(\text{PFK})}}\right)^2\cdot\left(\frac{\left(1+\frac{\text{Cf26}(\text{PFK})\cdot[\text{fructose 2,6-bisphosphate}]}{K_{\text{f26}}(\text{PFK})}+\frac{\text{Cf16}(\text{PFK})\cdot[\text{fructose 1,6-bisphosphate}]}{K_{\text{f16}}(\text{PFK})}\right)}{1+\frac{[\text{fructose 2,6-bisphosphate}]}{K_{\text{f26}}(\text{PFK})}+\frac{[\text{fructose 1,6-bisphosphate}]}{K_{\text{f16}}(\text{PFK})}}\right)^2\cdot\left(1+\frac{\text{Catp}(\text{PFK})\cdot[\text{ATP}]}{K_{\text{atp}}(\text{PFK})}\right)^2}$$

$$v_{\text{PGI}}=[\text{PGI1}]\cdot k_{\text{cat}}(\text{PGI})\cdot\left(\frac{[\text{glucose 6-phosphate}]}{K_{\text{g6p}}(\text{PGI})}-\frac{[\text{fructose 6-phosphate}]}{K_{\text{g6p}}(\text{PGI})\cdot K_{\text{eq PGI}}}\right)$$
$$\cdot\left(\frac{1}{1+\frac{[\text{glucose 6-phosphate}]}{K_{\text{g6p}}(\text{PGI})}+\frac{[\text{fructose 6-phosphate}]}{K_{\text{f6p}}(\text{PGI})}}\right)$$

$$v_{\text{PGK1}}=[\text{PGK1}]\cdot k_{\text{cat}}(\text{PGK1})\cdot\left(\frac{[\text{ADP}]}{K_{\text{adp}}(\text{PGK1})}\right)^{n_{\text{Hadp}}(\text{PGK1})-1}\cdot$$
$$\left(\frac{[\text{1,3-bisphosphoglycerate}]\cdot[\text{ADP}]}{K_{\text{bpg}}(\text{PGK1})\cdot K_{\text{adp}}(\text{PGK1})}-\frac{[\text{3-phosphoglycerate}]\cdot[\text{ATP}]}{K_{\text{bpg}}(\text{PGK1})\cdot K_{\text{adp}}(\text{PGK1})\cdot K_{\text{eq}}(\text{PGK1})}\right)\cdot$$
$$\frac{1}{\left(1+\frac{[\text{1,3-bisphosphoglycerate}]}{K_{\text{bpg}}(\text{PGK1})}+\frac{[\text{3-phosphoglycerate}]}{K_{\text{p3g}}(\text{PGK1})}\right)\cdot\left(1+\left(\frac{[\text{ADP}]}{K_{\text{adp}}(\text{PGK1})}\right)^{n_{\text{Hadp}}(\text{PGK1})}+\frac{[\text{ATP}]}{K_{\text{atp}}(\text{PGK1})}\right)})$$

$$v_{\text{PGM}}=V_{\max}(\text{PGM})\cdot\left(\frac{[\text{glucose 6-phosphate}]}{K_{\text{g6p}}(\text{PGM})}-\frac{[\text{glucose 1-phosphate}]}{K_{\text{g6p}}(\text{PGM})\cdot K_{\text{eq PGM}}}\right)$$
$$\cdot\left(\frac{1}{1+\frac{[\text{glucose 6-phosphate}]}{K_{\text{g6p}}(\text{PGM})}+\frac{[\text{glucose 1-phosphate}]}{K_{\text{g1p}}(\text{PGM})}}\right)$$

$$v_{\text{PYK2}}=\frac{\frac{[\text{PYK2}]\cdot k_{\text{cat}}(\text{PYK2})\cdot\left([\text{phosphoenolpyruvate}]\cdot[\text{ADP}]-\frac{[\text{pyruvate}]\cdot[\text{ATP}]}{K_{\text{eq PYK}}}\right)}{K_{\text{pep}}(\text{PYK2})\cdot K_{\text{adp}}(\text{PYK2})}}{\left(1+\frac{[\text{phosphoenolpyruvate}]}{K_{\text{pep}}(\text{PYK2})}+\frac{[\text{pyruvate}]}{K_{\text{pyr}}(\text{PYK2})}+L_0(\text{PYK2})\cdot\frac{\frac{[\text{ATP}]}{K_{\text{iATP}}(\text{PYK2})}+1}{\frac{[\text{fructose 1,6-bisphosphate}]}{K_{\text{f16p}}(\text{PYK2})}+1}\right)\cdot\left(1+\frac{[\text{ADP}]}{K_{\text{adp}}(\text{PYK2})}+\frac{[\text{ATP}]}{K_{\text{atp}}(\text{PYK2})}\right)}$$

$$v_{\text{succinate branch}}=k_{\text{succinate branch}}\cdot[\text{pyruvate}]\cdot[\text{NAD}]$$

$$v_{\text{T6P synthase}}=\frac{V_{\max}(\text{T6P synthase})\cdot[\text{glucose 6-phosphate}]\cdot[\text{UDP glucose}]}{K_{\text{g6p}}(\text{T6P synthase})\cdot K_{\text{udg}}(\text{T6P synthase})}\cdot\frac{1}{\left(1+\frac{[\text{glucose 6-phosphate}]}{K_{\text{g6p}}(\text{T6P synthase})}\right)\cdot\left(1+\frac{[\text{UDP glucose}]}{K_{\text{udg}}(\text{T6P synthase})}\right)}$$

$$v_{\text{TDH1}}=\frac{[\text{TDH1}]\cdot k_{\text{cat}}(\text{TDH1})\cdot\left(\frac{[\text{glyceraldehyde 3-phosphate}]\cdot[\text{NAD}]}{K_{\text{gap}}(\text{TDH1})\cdot K_{\text{nad}}(\text{TDH1})}-\frac{[\text{1,3-bisphosphoglycerate}]\cdot[\text{NADH}]}{K_{\text{gap}}(\text{TDH1})\cdot K_{\text{nad}}(\text{TDH1})\cdot K_{\text{eq-TDH}}}\right)}{\left(1+\frac{[\text{glyceraldehyde 3-phosphate}]}{K_{\text{gap}}(\text{TDH1})}+\frac{[\text{1,3-bisphosphoglycerate}]}{K_{\text{bpg}}(\text{TDH1})}\right)\cdot\left(1+\frac{[\text{NAD}]}{K_{\text{nad}}(\text{TDH1})}+\frac{[\text{NADH}]}{K_{\text{nadh}}(\text{TDH1})}\right)}$$

$$v_{\text{TDH2}}=\frac{[\text{TDH2}]\cdot k_{\text{cat}}(\text{TDH2})\cdot\left(\frac{[\text{glyceraldehyde 3-phosphate}]\cdot[\text{NAD}]}{K_{\text{gap}}(\text{TDH2})\cdot K_{\text{nad}}(\text{TDH2})}-\frac{[\text{1,3-bisphosphoglycerate}]\cdot[\text{NADH}]}{K_{\text{gap}}(\text{TDH2})\cdot K_{\text{nad}}(\text{TDH2})\cdot K_{\text{eq-TDH}}}\right)}{\left(1+\frac{[\text{glyceraldehyde 3-phosphate}]}{K_{\text{gap}}(\text{TDH2})}+\frac{[\text{1,3-bisphosphoglycerate}]}{K_{\text{bpg}}(\text{TDH2})}\right)\cdot\left(1+\frac{[\text{NAD}]}{K_{\text{nad}}(\text{TDH2})}+\frac{[\text{NADH}]}{K_{\text{nadh}}(\text{TDH2})}\right)}$$

$$v_{\text{TDH3}}=\frac{[\text{TDH3}]\cdot k_{\text{cat}}(\text{TDH3})\cdot\left(\frac{[\text{glyceraldehyde 3-phosphate}]\cdot[\text{NAD}]}{K_{\text{gap}}(\text{TDH3})\cdot K_{\text{nad}}(\text{TDH3})}-\frac{[\text{1,3-bisphosphoglycerate}]\cdot[\text{NADH}]}{K_{\text{gap}}(\text{TDH3})\cdot K_{\text{nad}}(\text{TDH3})\cdot K_{\text{eq-TDH}}}\right)}{\left(1+\frac{[\text{glyceraldehyde 3-phosphate}]}{K_{\text{gap}}(\text{TDH3})}+\frac{[\text{1,3-bisphosphoglycerate}]}{K_{\text{bpg}}(\text{TDH3})}\right)\cdot\left(1+\frac{[\text{NAD}]}{K_{\text{nad}}(\text{TDH3})}+\frac{[\text{NADH}]}{K_{\text{nadh}}(\text{TDH3})}\right)}$$

$$v_{\text{TPI}}=\frac{\frac{[\text{TPI1}]\cdot k_{\text{cat}}(\text{TPI})}{K_{\text{dhap}}(\text{TPI})}\cdot\left([\text{dihydroxyacetone phosphate}]-\frac{[\text{glyceraldehyde 3-phosphate}]}{K_{\text{eq}}(\text{TPI})}\right)}{\left(1+\frac{[\text{dihydroxyacetone phosphate}]}{K_{\text{dhap}}(\text{TPI})}+\frac{[\text{glyceraldehyde 3-phosphate}]}{K_{\text{gap}}(\text{TPI})}\right)\cdot\left(1+\frac{[\text{glyceraldehyde 3-phosphate}]^4}{K_{\text{igap}}(\text{TPI})^4}\right)}.$$

$$v_{\text{UDP to UTP pseudoreaction}}=k(\text{UDP to UTP pseudoreaction})\cdot[\text{UDP}]\cdot[\text{ATP}]$$

$$v_{\text{UDP-glucose phosphorylase}}=\frac{V_{\max}(\text{UDP-glucose phosphorylase})\cdot[\text{UTP}]\cdot[\text{glucose 1-phosphate}]}{K_{\text{utp}}(\text{UDP-glucose phosphorylase})\cdot K_{\text{g1p}}(\text{UDP-glucose phosphorylase})}\cdot$$
$$\frac{1}{\left(\frac{K_{\text{iutp}}(\text{UDP-glucose phosphorylase})}{K_{\text{utp}}(\text{UDP-glucose phosphorylase})}+\frac{[\text{UTP}]}{K_{\text{utp}}(\text{UDP-glucose phosphorylase})}+\frac{[\text{glucose 1-phosphate}]}{K_{\text{g1p}}(\text{UDP-glucose phosphorylase})}+\frac{[\text{UTP}]\cdot[\text{glucose 1-phosphate}]}{K_{\text{g1p}}(\text{UDP-glucose phosphorylase})\cdot K_{\text{utp}}(\text{UDP-glucose phosphorylase})}+\frac{K_{\text{iutp}}(\text{UDP-glucose phosphorylase})\cdot[\text{UDP-glucose}]}{K_{\text{utp}}(\text{UDP-glucose phosphorylase})\cdot K_{\text{iudg}}(\text{UDP-glucose phosphorylase})}+\frac{[\text{glucose 1-phosphate}]\cdot[\text{UDP-glucose}]}{K_{\text{g1p}}(\text{UDP-glucose phosphorylase})\cdot K_{\text{udg}}(\text{UDP-glucose phosphorylase})}\right)}$$

2 Differential Equations

$$
\begin{aligned}
\frac{d[\text{ADP}]}{dt} &= +v_{\text{UDP to UTP pseudoreaction}} - v_{\text{PYK2}} - v_{\text{CDC19}} - v_{\text{PGK1}} + v_{\text{PFK}} + v_{\text{HXK2}} + v_{\text{HXK1}} + v_{\text{GLK1}} + v_{\text{ATPase}} - 2 \cdot v_{\text{AK}} \\
\frac{d[\text{ATP}]}{dt} &= -v_{\text{UDP to UTP pseudoreaction}} + v_{\text{PYK2}} + v_{\text{CDC19}} + v_{\text{PGK1}} - v_{\text{PFK}} - v_{\text{HXK2}} - v_{\text{HXK1}} - v_{\text{GLK1}} - v_{\text{ATPase}} + v_{\text{AK}} \\
\frac{d[\text{acetaldehyde}]}{dt} &= +v_{\text{PDC6}} + v_{\text{PDC5}} + v_{\text{PDC1}} - v_{\text{acetate branch}} - v_{\text{ADH5}} - v_{\text{ADH1}} \\
\frac{d[\text{1,3-bisphosphoglycerate}]}{dt} &= +v_{\text{TDH1}} - v_{\text{PGK1}} + v_{\text{TDH2}} + v_{\text{TDH3}} \\
\frac{d[\text{dihydroxyacetone phosphate}]}{dt} &= +v_{\text{FBA}} - v_{\text{TPI}} - v_{\text{Glycerol3PDH}} \\
\frac{d[\text{fructose 1,6-bisphosphate}]}{dt} &= +v_{\text{PFK}} - v_{\text{FBA}} \\
\frac{d[\text{fructose 6-phosphate}]}{dt} &= +v_{\text{PGI}} - v_{\text{PFK}} \\
\frac{d[\text{glucose 1-phosphate}]}{dt} &= +v_{\text{PGM}} - v_{\text{UDP-glucose phosphorylase}} \\
\frac{d[\text{glycerol 3-phosphate}]}{dt} &= -v_{\text{Glycerol3P}} + v_{\text{Glycerol3PDH}} \\
\frac{d[\text{glucose 6-phosphate}]}{dt} &= -v_{\text{PGM}} - v_{\text{PGI}} + v_{\text{HXK2}} + v_{\text{HXK1}} + v_{\text{GLK1}} - v_{\text{T6Psynth}} \\
\frac{d[\text{glyceraldehyde 3-phosphate}]}{dt} &= -v_{\text{TDH1}} + v_{\text{FBA}} + v_{\text{TPI}} - v_{\text{TDH3}} - v_{\text{TDH2}} \\
\frac{d[\text{glucose cell}]}{dt} &= +v_{\text{HXT}} - v_{\text{HXK2}} - v_{\text{HXK1}} - v_{\text{GLK1}} \\
\frac{d[\text{NAD}]}{dt} &= -v_{\text{TDH1}} - 3 \cdot v_{\text{succinate branch}} + v_{\text{Glycerol3PDH}} - v_{\text{acetate branch}} + v_{\text{ADH5}} + v_{\text{ADH1}} - v_{\text{TDH3}} - v_{\text{TDH2}} \\
\frac{d[\text{2-phosphoglycerate}]}{dt} &= +v_{\text{GPM1}} - v_{\text{ENO1}} - v_{\text{ENO2}} \\
\frac{d[\text{3-phosphoglycerate}]}{dt} &= +v_{\text{PGK1}} - v_{\text{GPM1}} \\
\frac{d[\text{phosphoenolpyruvate}]}{dt} &= -v_{\text{PYK2}} - v_{\text{CDC19}} + v_{\text{ENO1}} + v_{\text{ENO2}} \\
\frac{d[\text{pyruvate}]}{dt} &= +v_{\text{PYK2}} + v_{\text{CDC19}} - v_{\text{PDC6}} - v_{\text{PDC5}} - v_{\text{PDC1}} - v_{\text{succinate branch}} \\
\frac{d[\text{trehalose 6-phosphate}]}{dt} &= +v_{\text{T6Psynth}} - v_{\text{T6P phosphatase}} \\
\frac{d[\text{UDP}]}{dt} &= -v_{\text{UDP to UTP pseudoreaction}} + v_{\text{T6Psynth}} \\
\frac{d[\text{UTP}]}{dt} &= +v_{\text{UDP to UTP pseudoreaction}} - v_{\text{UDP-glucose phosphorylase}}
\end{aligned}
$$

3 Mass Conservation

$$
\begin{aligned}
[\text{AMP}] &= \sum \text{AXP} - [\text{ATP}] - [\text{ADP}] \\
[\text{NADH}] &= \sum \text{NAD} - [\text{NAD}] \\
[\text{UDP-glucose}] &= \sum \text{UXP} - [\text{UTP}] - [\text{UDP}] \\
\text{energy charge} &= \frac{[\text{ATP}] + \frac{[\text{ADP}]}{2}}{\sum \text{AXP}}
\end{aligned}
$$

$$k_{\mathrm{acetate}} = 0.0055434$$
$$k_{\mathrm{cat\_adh1}} = 176$$
$$k_{\mathrm{acald\_adh1}} = 0.4622$$
$$k_{\mathrm{inadh\_adh1}} = 0.031$$
$$k_{\mathrm{nadh\_adh1}} = 0.11$$
$$k_{\mathrm{etoh\_adh1}} = 17$$
$$k_{\mathrm{inad\_adh1}} = 0.920$$
$$k_{\mathrm{nad\_adh1}} = 0.17$$
$$k_{\mathrm{ietoh\_adh1}} = 90$$
$$k_{\mathrm{iacald\_adh1}} = 1.1$$
$$k_{\mathrm{eq\_adh}} = 14492.8$$
$$k_{\mathrm{cat\_adh5}} = 0$$
$$k_{\mathrm{acald\_adh5}} = 1.1$$
$$k_{\mathrm{inadh\_adh5}} = 0.031$$
$$k_{\mathrm{nadh\_adh5}} = 0.11$$
$$k_{\mathrm{etoh\_adh5}} = 17$$
$$k_{\mathrm{inad\_adh5}} = 0.920$$
$$k_{\mathrm{nad\_adh5}} = 0.17$$
$$k_{\mathrm{ietoh\_adh5}} = 90$$
$$k_{\mathrm{iacald\_adh5}} = 1.1$$
$$k_{\mathrm{eq\_ak}} = 0.45$$
$$k_{\mathrm{ak}} = 0.75$$
$$k_{\mathrm{atp\_atp}} = 3$$
$$V_{\mathrm{max\_atp}} = 6.16$$
$$k_{\mathrm{cat\_cdc19}} = 20.146$$
$$k_{\mathrm{eq\_pyk}} = 6500$$
$$k_{\mathrm{pep\_cdc19}} = 0.281$$
$$k_{\mathrm{adp\_cdc19}} = 0.243$$
$$k_{\mathrm{pyr\_cdc19}} = 21$$
$$L_0^{\mathrm{cdc19}} = 100$$
$$k_{i,\mathrm{atp\_cdc19}} = 9.3$$
$$k_{f16,\mathrm{cdc19}} = \frac{1}{5}$$
$$k_{\mathrm{atp\_cdc19}} = 1.5$$
$$k_{\mathrm{cat\_eno1}} = 7.6$$
$$k_{p2g,\mathrm{eno1}} = 0.043$$

$$k_{\mathrm{eq\_eno1}} = 6.700$$
$$k_{\mathrm{pep\_eno1}} = \frac{1}{2}$$
$$k_{\mathrm{cat\_eno2}} = 19.87$$
$$k_{p2g,\mathrm{eno2}} = 0.104$$
$$k_{\mathrm{eq\_eno2}} = k_{\mathrm{eq\_eno1}}$$
$$k_{\mathrm{pep\_eno2}} = \frac{1}{2}$$
$$k_{\mathrm{cat\_fba}} = 4.139$$
$$k_{f16,\mathrm{fba}} = 0.4507$$
$$k_{\mathrm{eq\_fba}} = 0.069$$
$$k_{\mathrm{dhap\_fba}} = 2$$
$$k_{\mathrm{gap\_fba}} = 2.4$$
$$k_{i,\mathrm{gap\_fba}} = 10$$
$$k_{\mathrm{cat\_glk1}} = 0.0721$$
$$k_{\mathrm{glc\_glk1}} = 0.0106$$
$$k_{\mathrm{atp\_glk1}} = 0.865$$
$$k_{\mathrm{eq\_HXK}} = 2000$$
$$k_{\mathrm{g6p\_glk1}} = 30$$
$$k_{\mathrm{adp\_glk1}} = 0.23$$
$$V_{\mathrm{max\_glycerol3p}} = 0.8833333$$
$$k_{\mathrm{g3p\_glycerol3p}} = 3.5$$
$$V_{\mathrm{max\_glycerol3pdh}} = 0.7833333$$
$$k_{\mathrm{dhap\_glycerol3pdh}} = 0.54$$
$$k_{\mathrm{nadh\_glycerol3pdh}} = 0.023$$
$$k_{\mathrm{eq\_glycerol3pdh}} = 10000$$
$$k_{fbp,\mathrm{glycerol3pdh}} = 4.8$$
$$k_{\mathrm{atp\_glycerol3pdh}} = 0.73$$
$$k_{\mathrm{adp\_glycerol3pdh}} = 2$$
$$k_{\mathrm{g3p\_glycerol3pdh}} = 1.2$$
$$k_{\mathrm{nad\_glycerol3pdh}} = 0.93$$
$$k_{\mathrm{cat\_gpm1}} = 400$$
$$k_{\mathrm{eq\_gpm1}} = 0.19$$
$$k_{\mathrm{p3g\_gpm1}} = 1.2$$
$$k_{\mathrm{p2g\_gpm1}} = 1.41$$
$$k_{\mathrm{cat\_hxk1}} = 10.2$$
$$k_{\mathrm{eq\_hxk1}} = k_{\mathrm{eq\_HXK}}$$

$$k_{\text{glc\_hxk1}} = 0.15$$
$$k_{\text{g6p\_hxk1}} = 30$$
$$k_{i,\text{t6p\_hxk1}} = \frac{1}{5}$$
$$k_{\text{atp\_hxk1}} = 0.293$$
$$k_{\text{adp\_hxk1}} = 0.230$$
$$k_{\text{cat\_hxk2}} = 63.1$$
$$k_{\text{eq\_hxk2}} = k_{\text{eq\_HXK}}$$
$$k_{\text{glc\_hxk2}} = \frac{1}{5}$$
$$k_{\text{g6p\_hxk2}} = 30$$
$$k_{i,\text{t6p\_hxk2}} = 0.04$$
$$k_{\text{atp\_hxk2}} = 0.195$$
$$k_{\text{adp\_hxk2}} = 0.230$$
$$k_{\text{glc\_hxt}} = 0.9$$
$$V_{\text{max\_hxt}} = 3.35$$
$$k_{i,\text{hxt}} = 0.91$$
$$k_{\text{cat\_pdc1}} = 12.14$$
$$k_{\text{pyr\_pdc1}} = 8.5$$
$$k_{\text{cat\_pdc5}} = 10.32$$
$$k_{\text{pyr\_pdc5}} = 7.08$$
$$k_{\text{cat\_pdc6}} = 9.21$$
$$k_{\text{pyr\_pdc6}} = 2.92$$
$$k_{\text{cat\_pfk}} = 209.6$$
$$k_{\text{eq\_pfk}} = 800$$
$$k_{\text{f6p\_pfk}} = 0.1$$
$$g_{R,\text{pfk}} = 5.12$$
$$k_{\text{adp\_pfk}} = 1$$
$$L_0^{\text{pfk}} = 0.66$$
$$c_{i,\text{atp\_pfk}} = 100$$
$$k_{i,\text{atp\_pfk}} = 0.65$$
$$c_{\text{amp\_pfk}} = 0.0845$$
$$k_{\text{amp\_pfk}} = 0.0995$$
$$c_{f26,\text{pfk}} = 0.0174$$
$$c_{f16,\text{pfk}} = 0.397$$
$$k_{f26,\text{pfk}} = 0.000682$$
$$k_{f16,\text{pfk}} = 0.111$$

$$
\begin{aligned}
c_{\text{atp\_pfk}} &= 3 \\
k_{\text{atp\_pfk}} &= 0.71 \\
k_{\text{cat\_pgk1}} &= 58.6 \\
k_{\text{adp\_pgk1}} &= 0.2 \\
k_{\text{bpg\_pgk1}} &= 0.003 \\
k_{\text{eq\_pgk1}} &= 3200 \\
k_{\text{p3g\_pgk1}} &= 4.58 \\
k_{\text{atp\_pgk1}} &= 1.99 \\
V_{\text{max\_pgm}}(1:N) &= 0.12762 \\
k_{\text{eq\_pgm}} &= 0.1667 \\
k_{\text{g6p\_pgm}} &= 0.05 \\
k_{\text{g1p\_pgm}} &= 0.023 \\
k_{\text{cat\_pyk2}} &= 0 \\
k_{\text{pep\_pyk2}} &= 0.19 \\
k_{\text{pyr\_pyk2}} &= 21 \\
L_0^{\text{pyk2}} &= 100 \\
k_{i,\text{atp\_pyk2}} &= 9.3 \\
k_{f16p,\text{pyk2}} &= \frac{1}{5} \\
k_{\text{adp\_pyk2}} &= 0.3 \\
k_{\text{atp\_pyk2}} &= 1.5 \\
k_{\text{succ}} &= 0 \\
k_{\text{cat\_tdh1}} &= 19.12 \\
k_{\text{eq\_tdh1}} &= 0.00533413 \\
k_{\text{gap\_tdh1}} &= 0.495 \\
k_{\text{bpg\_tdh1}} &= 0.0098 \\
k_{\text{nad\_tdh1}} &= 0.09 \\
k_{\text{ndah\_tdh1}} &= 0.06 \\
k_{\text{cat\_tdh2}} &= 8.633 \\
k_{\text{eq\_tdh2}} &= k_{\text{eq\_tdh1}} \\
k_{\text{gap\_tdh2}} &= 0.77 \\
k_{\text{bpg\_tdh2}} &= 0.0098 \\
k_{\text{nad\_tdh2}} &= 0.09 \\
k_{\text{ndah\_tdh2}} &= 0.06 \\
k_{\text{cat\_tdh3}} &= 18.162 \\
k_{\text{eq\_tdh3}} &= k_{\text{eq\_tdh1}} \\
k_{\text{gap\_tdh3}} &= 0.423
\end{aligned}
$$

$$k_{\text{bpg\_tdh3}} = 0.909$$
$$k_{\text{nad\_tdh3}} = 0.09$$
$$k_{\text{ndah\_tdh3}} = 0.06$$
$$k_{\text{cat\_tpi}} = 564.38$$
$$k_{\text{eq\_tpi}} = 0.045$$
$$k_{\text{dhap\_tpi}} = 6.454$$
$$k_{\text{gap\_tpi}} = 5.25$$
$$k_{i,\text{gap\_tpi}} = 35.1$$
$$V_{\text{max\_udpglucose}} = 13.25552$$
$$k_{i,\text{utp\_glucose}} = 0.11$$
$$k_{\text{utp\_glucose}} = 0.11$$
$$k_{\text{g1p\_udpglucose}} = 0.32$$
$$k_{i,\text{udg\_udp}} = 0.0035$$
$$k_{\text{udp\_utp}} = 0.07452582941$$
$$k_{\text{cat\_pgi1}} = 487.36$$
$$k_{\text{g6p\_pgi}} = 1.0257$$
$$k_{\text{eq\_pgi}} = 0.29$$
$$k_{\text{f6p\_pgi}} = 0.307$$
$$V_{\text{max\_t6psynthase}}(1:N) = 0.49356$$
$$k_{\text{g6p\_t6psyn}} = 3.8$$
$$k_{\text{udg\_t6psyn}} = 0.886$$
$$V_{\text{max\_t6p}} = 2.34$$
$$k_{\text{t6pphos}} = \frac{1}{2}$$
$$E_{\text{adh1}}(1:N) = 0.1639085109$$
$$E_{\text{adh5}}(1:N) = 0.004249984225$$
$$E_{\text{cdc19}}(1:N) = 2.04839$$
$$E_{\text{eno1}}(1:N) = 0.686372$$
$$E_{\text{eno2}}(1:N) = 1.97445$$
$$E_{\text{fba}}(1:N) = 1.33839$$
$$E_{\text{glk1}}(1:N) = 0.04508696244$$
$$E_{\text{gpm1}}(1:N) = 0.73$$
$$E_{\text{hxk1}}(1:N) = 0.01678074571$$
$$E_{\text{hxk2}}(1:N) = 0.0613313537$$
$$E_{\text{pdc1}}(1:N) = 1.06781$$
$$E_{\text{pdc5}}(1:N) = 0.0123547$$
$$E_{\text{pdc6}}(1:N) = 0.00654086$$

$$E_{\text{pfk1}}(1:N) = 0.046785$$
$$E_{\text{pfk2}}(1:N) = 0.0391$$
$$E_{\text{F26bP}}(1:N) = 0.0030$$
$$E_{\text{pgk1}}(1:N) = 0.257657$$
$$E_{\text{pyk2}}(1:N) = 0.00606994$$
$$E_{\text{tdh1}}(1:N) = 0.350865$$
$$E_{\text{tdh2}}(1:N) = 0$$
$$E_{\text{tdh3}}(1:N) = 4.2044$$
$$E_{\text{tpi}}(1:N) = 0.294358$$
$$E_{\text{pgi}}(1:N) = 0.138291$$
$$E_{\text{ethanol}}(1:N) = 221.8903114$$
$$\text{glucose}_{\text{extracellular}}(1:N) = 74$$
$$\text{Diff}_{\text{ADP}} = 5$$
$$\text{Diff}_{\text{ATP}} = 5$$
$$\text{Diff}_{\text{acetaldehyde}} = 5$$
$$\text{Diff}_{\text{bisphosphoglycerate\_13}} = 5$$
$$\text{Diff}_{\text{dihydroxyacetone\_phosphate}} = 5$$
$$\text{Diff}_{\text{fructose\_biphosphate}} = 5$$
$$\text{Diff}_{\text{fructose\_phosphate}} = 5$$
$$\text{Diff}_{\text{glucose1\_phosphate}} = 5$$
$$\text{Diff}_{\text{glycerol\_phosphate}} = 5$$
$$\text{Diff}_{\text{glucose6\_phosphate}} = 5$$
$$\text{Diff}_{\text{glyceraldehyde\_phosphate}} = 5$$
$$\text{Diff}_{\text{glucose\_cell}} = 5$$
$$\text{Diff}_{\text{NAD}} = 5$$
$$\text{Diff}_{\text{phosphoglycerate\_2}} = 5$$
$$\text{Diff}_{\text{phosphoglycerate\_3}} = 5$$
$$\text{Diff}_{\text{phosphoenolpyruvate}} = 5$$
$$\text{Diff}_{\text{pyruvate}} = 5$$
$$\text{Diff}_{\text{trehalose\_phosphate}} = 5$$
$$\text{Diff}_{\text{UDP}} = 5$$
$$\text{Diff}_{\text{NADH}} = 5$$
$$\text{Diff}_{\text{UTP}} = 5$$
$$\text{Diff}_{\text{AMP}} = 5$$
$$\text{Diff}_{\text{UDG}} = 5$$
